# Electron Donor–Acceptor Chromophore Assembly as an Enabling Process for the Overall Light-Driven Reduction of Phosphine Oxides

**DOI:** 10.1021/acs.orglett.5c05235

**Published:** 2026-01-21

**Authors:** Thuan T. Tran, Anna I. Arkhypchuk, Andreas Orthaber, Sascha Ott

**Affiliations:** Department of Chemistry - Ångström Laboratory, 8097 Uppsala University, Box 523, 751 20 Uppsala, Sweden

## Abstract

Phosphines make up one of the largest classes of organophosphorus compounds, with applications in academic research and industry. Their preparation from phosphine oxides is mechanistically and thermodynamically highly challenging but opens the possibility for phosphorus recycling. We show that phosphine oxides can be converted into their corresponding phosphonium salts and subsequently reduced in a light-driven reaction to obtain the desired phosphines in 40–80% overall yields in a one-pot procedure. The reported methodology is free of exogenously added photocatalysts and utilizes the *in situ* formation of a photoactive donor–acceptor complex for light absorption and charge separation. Overall, the presented photochemical approach to reducing phosphonium salts that arise from phosphine oxides represents a valuable alternative to classical thermochemical methods for phosphorus recycling.

## Graphical abstract

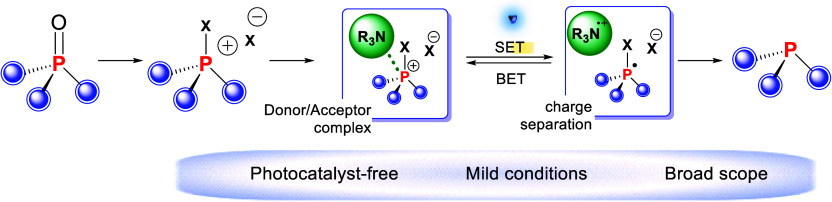

Recent advances in photochemistry have brought forward various nontraditional radical-based transformations under very mild reaction conditions.^1^ As many organic molecules do not absorb in the visible part of the solar spectrum themselves, direct photochemical syntheses have inherent limitations. This shortcoming can be remedied by the addition of an external chromophore, often based on an organic dye or a Ru- or Ir-based coordination compound, that triggers the organic redox transformation.^2^ The initiating step of the process is the absorption of a photon by the photocatalyst (PC) to generate a high-energy excited state (PC*), from which single-electron transfer (SET) processes with organic substrates occur that drive chemical redox transformations (Figure 1A).

**Figure 1:**
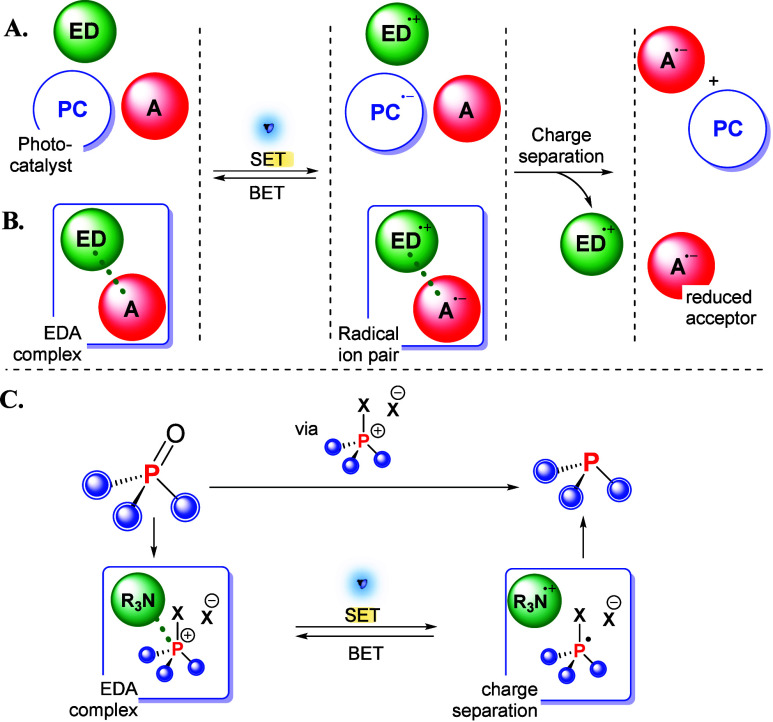
General representations of photoinduced electron transfers mediated by (A) a photocatalyst (PC) or (B) an electron donor–acceptor (EDA) complex. (C) Photoreduction of phosphine oxides to phosphines via phosphonium salts (this work). Abbreviations: ED, electron donor; A, acceptor; SET, single-electron transfer; BET, back electron transfer.

An external PC is, however, not an inevitable necessity to engage organic compounds that do not absorb visible light themselves in photochemical transformations. As exemplified for a reductive process in Figure 1B, an inherently electron-rich electron donor (ED) may electrostatically interact with electron-deficient organic substrates to form an electron donor–acceptor (EDA) complex, sometimes also termed a charge transfer complex. As the name suggests, the electronic absorption profiles of such EDA complexes differ from those of the individual components and may allow for visible light absorption without external PCs.^3^ Interestingly, the electronically excited state has a charge transfer nature and thus exhibits a directionality that aligns with that of the desired SET step, with the ED and acceptor carrying partial positive and negative charges, respectively, in the excited state. If the photogenerated radical ion pair escapes the solvent cage, charges are fully separated, and the organic substrate is reduced to its radical anion A^•–^, which can engage in further downstream chemistry.

Photochemical processes involving EDA complexes have been demonstrated as environmentally friendly alternatives to photoredox catalysis, omitting the use of external PCs. The strategy has been used mainly in an organic chemistry context but, to the best of our knowledge, is unexplored for the chemistry of main group compounds such as organophosphorus species. Apart from being valuable ligands for transition metal complexes^4^ or within organocatalysis,^5^ trivalent phosphines are used to mediate various important classical transformations in both academia and industry.^6^ In terms of photochemistry, a variety of phosphines were recently synthesized by radical cross-coupling of chlorophosphines with redox-active esters^7^ or by the activation of white phosphorus by aryl radicals.^8^ In all of these cases, the radicals were produced photocatalytically. We have reported the light-induced activation of halophosphines to prepare dimeric and cyclic oligophosphines, together with secondary phosphines.^9^ All of these recent reports have in common the fact that Ir-based PCs were employed to initiate the formation of organic radical anions.

In the context of sustainability, recycling of trivalent phosphines from their corresponding phosphine oxides is an important research topic,^10^ and two general strategies to achieve this transformation have been reported. The first is the direct reduction of the P=O bond with different silanes or lithium hydride-based reagents.^10b,10c^ An alternative to the direct reduction is a two-step process, in which phosphine oxides are first converted into more reactive phosphonium salts by treatment with oxalyl chloride. The thereby produced phosphonium salts can then be reduced by a variety of chemical reductants such as NaBH_4_,^11^ LiAlH_4_,^12^ and Si_2_Cl_6_.^13^ In addition, several protocols based on electrochemical reduction^14^ or reduction with hydrogen gas (80 bar, 130 °C) have been reported.^15^ In the present work, we hypothesized that the cationic charge of the halophosphonium salts would make them potential candidates to act as acceptor sites in EDA complexes with suitable electron donors, thereby offering opportunities for the recycling of trivalent phosphines under mild photochemical conditions. The benefit of the reaction would be that it is free of metals, in the form of metal-based chemical reductants or exogenous PCs. Herein, we describe our results on EDA formation between N-containing electron donors and halophosphonium salts and the use of these adducts in the light-driven preparation of phosphines (Figure 1C). Our method is a promising approach for sustainable transformations of phosphine oxides that overcome the disadvantages of conventional metal-mediated P=O bond reductions.^16^ We also provide mechanistic details that highlight the role of the electron donor beyond EDA formation, as it is shown that the donor may be chemically noninnocent and influence product speciation.

The validity of the approach to engage halophosphonium salts with amines in EDA formation was tested on [Ph_3_PCl]^+^Cl^–^ (**2a-Cl**), which was generated from Ph_3_PO (**1a**) by treatment with readily available oxalyl chloride.^17^ The first indications of the formation of an EDA complex were obtained from ^31^P NMR spectroscopy, which showed that the addition of (*i*Pr)_2_NEt (DIPEA) to a solution of **2a-Cl** in ACN-*d*_3_ induces an upfield shift in the ^31^P NMR spectrum from δ = 59.7 to 54.7 ppm (Figure 2A,B). Interestingly, when the experiment was conducted in THF-*d*_8_ instead, no such change was observed, and the signal at δ = 45.4 ppm remained unchanged. This difference in reactivity is attributed to the fact that **2a-Cl** in THF exists in its neutral pentavalent form, which seems to be not sufficiently electron-deficient to promote EDA complex formation. This negative experiment highlights the necessity of a charged electron acceptor for EDA formation and to drive the chemistry reported below.

**Figure 2:**
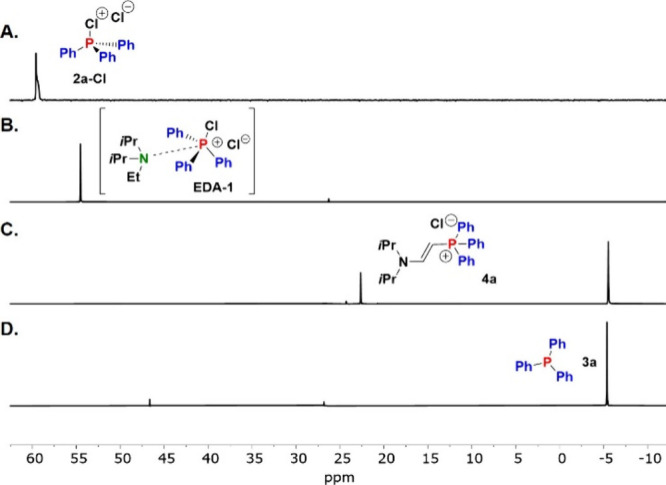
^31^P NMR spectra of **2a-Cl** in the (A) absence and (B) presence of DIPEA. ^31^P NMR spectra after irradiation of mixtures of **2a-Br** with (C) DIPEA and (D) (*n*Pr)_3_N.

The addition of DIPEA to **2a-Cl** also induces changes that are visible to the naked eye. While both individual components are colorless, their co-presence in one solution gives rise to a yellow color. Accordingly, a new band can be observed in the visible part of the UV/vis absorption spectrum of a mixture of **2a-Cl** and DIPEA at around 390 nm, tailing well beyond 600 nm. Notably, the new absorption features in the visible increase over time (see the Supporting Information for details), pointing toward a dynamic situation in which the absorption profile is convoluted by a concomitant chemical reaction.

Density functional theory (DFT) calculations at the GD3-U3LYP/6-311++G**/IEF-PCM­(ACN) level were carried out to identify potential modes of interaction between the preferred points of **2a-Cl** and NMe_3_. While gas phase calculations suggest that bipyramidal geometries with two tightly bound chlorides are dominant, inclusion of solvent effects (ACN) through a continuum model approach clearly suggests that one chloride interaction is purely electrostatic in nature, and **2a-Cl** is best described as a phoshonium salt with a chloride counterion. The calculations thus correlate well with the experimental findings described above. Including solvent effects, several local minimum structures for the EDA complex could be identified with tetrahedral phosphonium environments and different amine locations, being associated with electrostatic interactions. No strongly stabilized geometry with a directed interaction such as a recently claimed σ hole interaction could be found computationally,^18^ and the experimental solution structure of the **R**_**3**_ **N**···**2a-Cl** complex is thus most likely a distribution of different microscopic states, all of which continuously interconvert. As exemplified for one model structure in Figure 3, all local minimum descriptions of the **R**_**3**_ **N**···**2a-Cl** complex reproduce the experimental absorption spectrum, with an absorption maximum (λ_max_) at 410 nm tailing into the visible range. The low-energy absorption is composed of several transitions involving, among others, charge transfer states with the amine as a donor (orbital 100) and virtual orbital 104, which is antibonding with respect to the P–Cl σ-bond.

**Figure 3:**
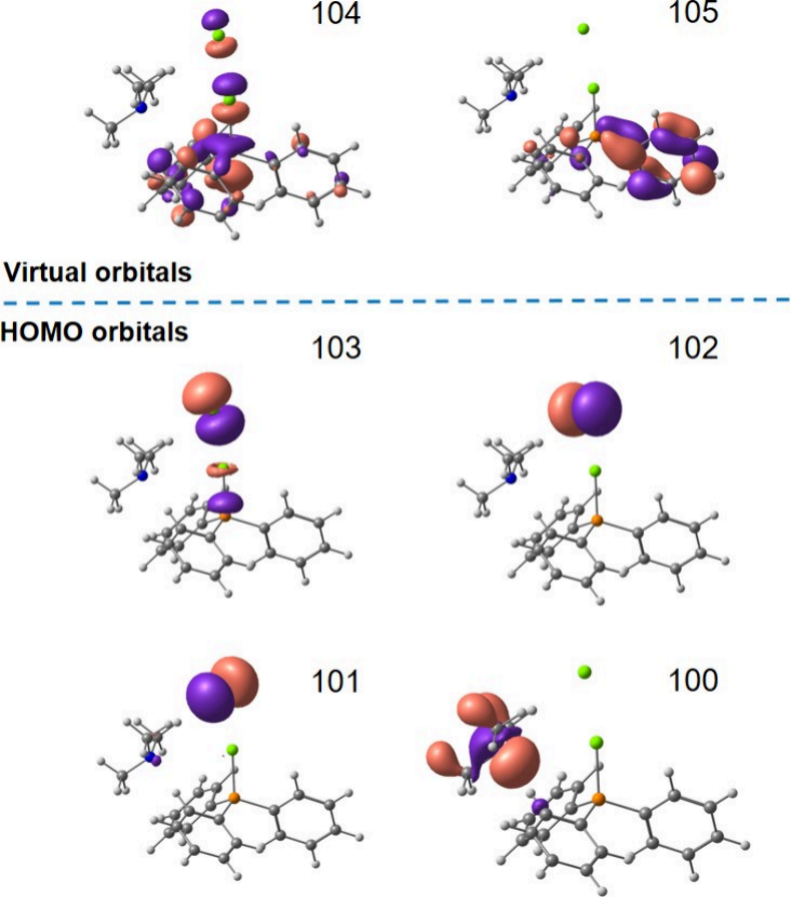
Selected molecular occupied (100–103) and virtual (104–105) orbitals of a local energy minimum structure of **R**_**3**_ **N**···**2a-Cl**, including solvent effects by a continuum model approach.

Having confirmed the donor–acceptor interaction, we investigated the photochemistry of different EDA complexes in detail by ^31^P and ^1^H NMR spectroscopy. In an initial experiment, a solution of **2a-Cl** and DIPEA in ACN-*d*_3_ was illuminated with a blue light. The reaction furnished triphenyl phosphine **3a** in 22% yield together with **4a**, which was repeatedly observed as a side product. The ^31^P NMR spectrum of **4a** features one singlet at δ = 22.8 ppm, well separated from that of product **3a** at δ = −5.4 ppm (Figure 2C).^17,19^ The ^1^H NMR spectrum of **4a** showed characteristic peaks that largely resembled those of the DIPEA donor, with additional resonances at δ = 6.44 and 4.61 ppm that are assigned to the protons of a double bond (see Figure S11 for more details).^19^ Together with mass spectrometric results, compound **4a** was identified as a product that arises from an irreversible reaction between species that originate from both **2a-Cl** and DIPEA. Compound **4a** was previously suggested by Li et al. but not characterized spectroscopically.^20^ Encouraged by this preliminary result, we altered reaction parameters to increase the product yield and to suppress the amount of **4a**. First, little variation in product distribution was found by changing the solvent from ACN-*d*_3_ to other polar solvents (Table S1, entry 2). In lower-polarity solvents, only trace amounts of the desired product were detected (Table S1, entry 3), consistent with the observation that **2a-Cl** prevails in the pentavalent Ph_3_PCl_2_ form,^21^ which does not engage in EDA complex formation, as described above. Without EDA formation, the system lacks the chromophore function and therefore does not react. Changing the donor from DIPEA to Et_3_N has little effect, while 2,6-lutidine gives only trace amounts of the product, presumably due to the low accessibility of the sterically congested N-donor atom that prevents EDA complex formation. Interestingly, the use of DABCO and (*n*Pr)_3_N results in 38% and 23% yields of the desired phosphine, respectively, but, more importantly, without the formation of any side product **4a** (Table S1, entries 6 and 7, respectively). These donors thus seem to suppress the reaction pathway that leads to the formation of the side product. A significant leap forward when it comes to yields of phosphine **3a** formation was made when the halogen substituent at the phosphonium salt was changed from chloro to bromo, and [Ph_3_PBr]^+^Br^+^ (**2a-Br**) produced triphenylphosphine **3a** in 62% yield, along with a 32% yield of **4a**. This observation is not completely unexpected as P–Br bonds are longer than P–Cl bonds, and compounds with P–Br bonds are thus more reactive. Consequently, the reaction is significantly accelerated, and the illumination times to achieve complete consumption of the phosphonium salts decrease considerably in the case of **2a-Br**. Shortened reaction times are generally associated with higher yields, as the hydrolysis of phosphonium salts to the initial phosphine oxides is diminished. Interestingly, even the nature of the outer-sphere counterion impacts product distribution, albeit to a lesser extent than the P substituent. Mixed halogen complex [Ph_3_PCl]^+^Br^–^ exhibits a reactivity between those of [Ph_3_PCl]^+^Cl^–^ and [Ph_3_PBr]^+^Br^–^.

While [Ph_3_POTMS]^+^OTf^–^ fails to give the desired product, [Ph_3_PCl]^+^OTf^–^ and [Ph_3_PBr]^+^OTf^–^ afford the product in moderate yields. The transformations are driven by light, as its absence completely stifles the reaction even upon the mixture being heated to 80 °C (Table S1, entries 14 and 15, respectively). Combining the results from the donor screening study with the observation that **2a-Br** is the more reactive substrate, we irradiated a solution of [Ph_3_PBr]^+^Br^–^ and (*n*Pr)_3_N. Under these conditions, phosphine **3a** was obtained in 70% yield without the formation of any side product **4a** (Figure 2D).

A mechanistic proposal that accounts for all of the observations described above is presented in Scheme 1. Upon excitation, SET occurs between the two components of the **R**_**3**_ **N**···**2a-Cl/Br** EDA complex to produce P^V^ radical **5** and the oxidized donor. The latter species is deprotonated to form radical species **6**, in analogy to recent reports on the decomposition of oxidized electron donors.^22^ Radicals **5** and **6** are both high-energy species and can engage in a hydrogen atom transfer to produce vinylamine **7**, together with pentavalent species **9** that can easily lose 1 equiv of HCl to afford product **3** (top pathway). Alternatively, the halogen atom of radical **5** may leave the compound earlier and abstract the H atom of **6**, thereby directly furnishing product **3** (pathway not explicitly shown).

**Scheme 1:**
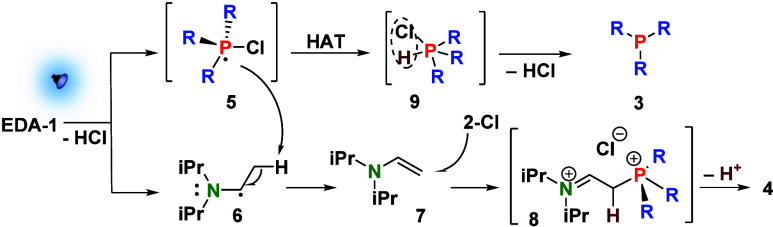
Potential Pathways for the Reaction of **2a-Cl** with DIPEA under Visible Light Illumination, Rationalizing the Formation of Phosphine **3** and Side Product **4** That Is Observed When the Amine Donor Contains *N*-Ethyl Groups

It is also important to state that it is unclear whether any of the postulated species will separately be solvated or whether the entire chemistry occurs within one and the same solvent cage. Regardless, the high reactivity of the radicals precludes their spectroscopic characterization and the acquisition of meaningful electrochemical data (see the Supporting Information for details). Vinylamine **7** is a highly plausible intermediate en route to observed side product **4**, the formation of which can be rationalized by a nucleophilic attack of the former at **2-Cl** to produce species **8**, which, upon deprotonation, gives rise to **4**. The proposed mechanism takes into consideration the fact that compounds like **4** are not experimentally observed when electron donors are used that do not contain *N*-ethyl groups. In their absence, the decomposition product of the oxidized donor does not contain a terminal vinyl group and is thus considerably less nucleophilic toward **2-Cl**. The mechanism suggests photogenerated radical intermediates after the light-induced charge separation and ionic, thermochemical steps further downstream to afford the observed products.

With a detailed mechanistic understanding of the reaction and optimized conditions in hand, the scope of the metal-free methodology for the light-driven reduction of phosphine oxides was explored (Scheme 2). All bromophosphonium bromides were generated *in situ* from the corresponding phosphine oxides by treatment with oxalyl bromide. Upon removal of residual oxalyl bromide under vacuum, the salts were used in the photoreaction without further purification. Compound **1b** with electron-withdrawing fluoride substituents is consumed after irradiation for only 3 h, and product **3b** obtained in 80% overall yield without any trace of side product **4b**, regardless of the nature of the electron donor. Phosphine oxides with electron-donating groups (EDGs) (**1c**–**e**) require longer reaction times under otherwise identical conditions and afford the corresponding phosphines in moderate yields. In these cases, the nature of the donor is highly important, as the use of DIPEA gives rise to large amounts of side products **4c**–**e**.

**Scheme 2:**
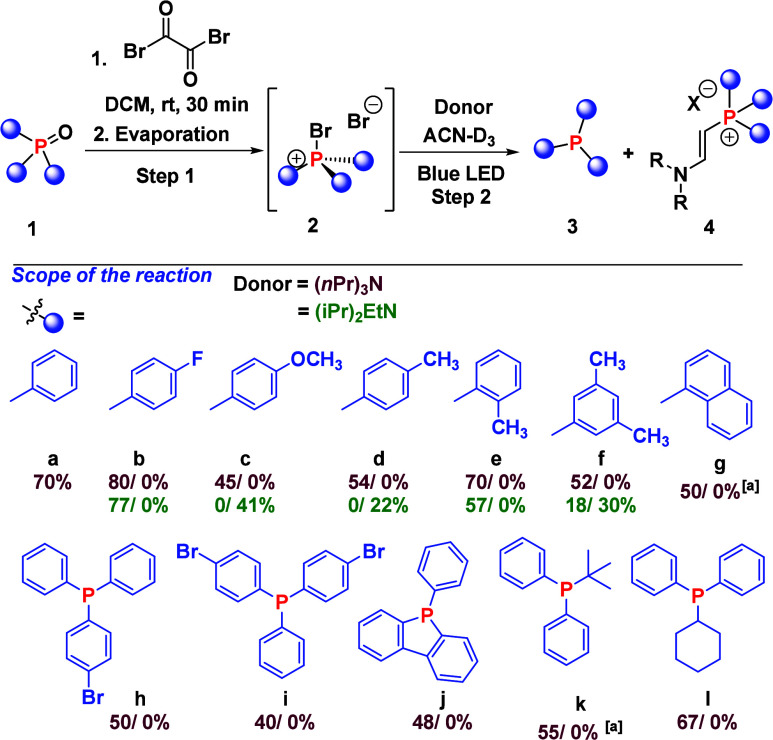
Substrate Scope for the One-Pot Activation/Reduction of Phosphine Oxides to Phosphines via Phosphonium Salts^a^

a The reaction was performed with chlorophosphonim salt [R_3_PCl]^+^Cl^–^.
b Standard conditions: donor (5 equiv), solvent (0.5 mL), blue light. Yields (**3**/**4**, in percent) are relative to internal standards in the NMR experiments.

Despite the steric impact of the tolyl substituents, tris­(*o*-tolyl)­phosphine oxide **1e** is reduced to the corresponding phosphine in 70% yield, and no **4e** is observed irrespective of the donor used (Table S2, entries 14–17). This is remarkable and may be ascribed to the steric bulk of the P radical species that hinders the reaction with the vinylamine. Moderate yields were obtained for sterically encumbered naphthalene phosphine **1g** and phosphole derivative **1j**. The strategy could also be applied to synthesize unsymmetric phosphine **3h** and **3i** in moderate yields. Further exploration of the reaction scope indicated that phosphine oxides **1k** and **1l** with alkyl substituents were tolerated, and products were afforded in 55% and 67% yields, respectively. However, under the basic conditions employed herein, [Ph_2_MePCl]^+^Cl^–^ (**2m-Cl**) is deprotonated at the methyl group and the transient ylide directly reacts with its phosphonium salt precursor to give undesired products (see Figure S52 for more details).^23^

In conclusion, a one-pot activation/photoreduction procedure that converts phosphine­(V) oxides into their corresponding phosphines via reactive phosphonium salts as enabling key intermediates is presented. This method allows for the synthesis of various aryl and alkyl phosphines in good to excellent yields under mild conditions employing (*n*Pr)_3_N as the electron donor (see the Supporting Information for comparisons with existing literature procedures). Spectroscopic studies demonstrate that the interaction between phosphonium salts and electron donors gives rise to EDA complexes that are the only light-absorbing species and enable the photoreduction. In addition, the reaction selectivity is strongly dependent on the steric size of adjacent organic groups and the nature of the electron donor. Tertiary phosphines are generally the major product, but notable quantities of side products are observed under certain conditions. Our presented methodology can serve as an alternative not only for the recycling of phosphines but also in light of sustainable phosphorus chemistry.

## Supplementary Material



## Data Availability

The data underlying this study are available in the published article and its Supporting Information.

## Notes

Synthetic procedures, purification protocols, compound characterization data, and NMR spectra (PDF)
