# Supplementary material for: Electron Donor–Acceptor Chromophore Assembly as an Enabling Process for the Overall Light-Driven Reduction of Phosphine Oxides
Source: Org Lett. 2026 Jan 21;28(4):1380–5. doi: 10.1021/acs.orglett.5c05235 (PMC12865801; doi:10.1021/acs.orglett.5c05235)
Supplement: Supplementary file 1 [file ol5c05235_si_001.pdf]

Electron Donor-Acceptor Chromophore Assembly as Enabling Process for the Overall Light-Driven Reduction of Phosphine Oxides

Thuan T. Tran, Anna I. Arkhypchuk, * Andreas Orthaber, Sascha Ott*

Dr. T. T. Tran, Dr. A. I. Arkhypchuk, Dr. A. Orthaber, Prof. Dr. S. Ott
Department of Chemistry – Ångström Laboratory
Uppsala University
Box 523, 751 20 Uppsala
E-mail: Anna.Arkhypchuk@kemi.uu.se; Sascha.Ott@kemi.uu.se

Table of content

1. General experimental information 3
  1.1 Synthetic considerations 3
  1.2 General procedure for the synthesis of the phosphine oxide 4
  1.3 Preparation of Halo-phosphonium salts 7
- Preparation of chlorotriphenylphosphonium chloride 7
- Preparation of bromotriphenylphosphonium bromide 8
- Preparation of Chlorotriphenylphosphonium bromide 8
- Preparation of $[Ph_3PCl]^+OTf^-$ 9
- Preparation of $[Ph_3PBr]^+OTf^-$ 9
- Preparation of $[Ph_3POTMS]^+OTf^-$ 9

2. Reaction optimization 10
3. One-pot procedure for conversion of phosphine(V) oxides to phosphines via chloro/bromophosphonium salts 11
4. Comparison of the developed methodology with literature procedures 15
5. NMR Spectroscopic data 16
6. NMR spectra 33
7. UV-Vis studies of EDA formation 88
8. Cyclic voltammetry 89
9. EPR spectroscopy 90
10. References 91

1. General experimental information

1.1 Synthetic considerations

Glassware and reaction conditions

Unless specified otherwise, all manipulations were carried out using glovebox techniques, in order to exclude air and moisture. Young NMR tube, glassware was flame-dried.

Chemicals and reagents

Commercially available phosphines, phosphine oxides, oxalyl chloride, oxalyl bromide, amines, cyclohexene were purchased from Sigma Aldrich, TCI and used without further purification. Other phosphine oxides and phosphonium salts were prepared according to literature as described in the Supporting Information. ACN, ACN-D_3, DCM-D_2 were dried by refluxing several hours under powdered CaH_2, distilled under argon and deoxygenated using standard freeze-pump-thaw technique (3 cycles) and stored over molecular sieves (3 Å). C_6D_6 and THF-D_8 were freshly distilled from potassium and deoxygenated using standard freeze-pump-thaw technique (3 cycles) and stored over molecular sieves (3 Å). DIPEA, Et_3N, $(n\text{Pr})_3N$ was freshly distilled from potassium and deoxygenated using standard freeze-pump-thaw technique (3 cycles) and stored over molecular sieves (3 Å).

Purification

Desired phosphines were purified by filtering the reaction mixture through a small pad of silica gel using DCM.

Characterization

^{1}H, ^{13}C, ^{31}P NMR spectra were recorded on a JEOL (400YH magnet) Resonance 400 MHz spectrometer. Chemical shifts δ are reported in ppm, coupling constants *J* in Hz, multiplicity (s, singlet; br, broad; d, doublet; t, triplet; q, quartet; m, multiplet). ^{1}H NMR and ^{13}C NMR chemical shifts are referenced to the residual protic solvent signal and ^{31}P NMR spectra externally to 85% H_3PO_4 (aq).

HRMS

Due to high moisture and oxygen sensitivity of all phosphonium salts, no HRMS data could be obtained for these substances. All attempts resulted in immediate hydrolysis of the compounds, and the formation of the corresponding phosphine oxides, which were the only species that could be observed by mass spectrometry.

Irradiation source

A Kesill Tuna Blue lamp was used for irradiation (wavelength 460 nm, A160WE Tuna Blue light source, Power supply: 100-240 V AC (input), 19-24V DC (output), https://www.marine-aquatics.eu/en/kessil-a160we-tuna-blue-led-lighting-40w).

UV/Vis Spectroscopy

The absorption spectra were recorded on a Varian Cary 5000 spectrophotometer. All measurements were performed in ACN (taken from the solvent purification system) unless

indicated otherwise. Quartz cells with 1 cm optical pathlengths were used for the room temperature measurements. The amines were added to a 2 mL solution of phosphonium salts (2 mM) in ACN.

1.2 General procedure for the synthesis of the phosphine oxide

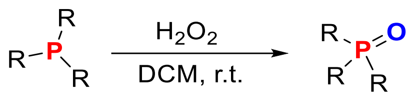

Scheme S1: *General procedure for the synthesis of the phosphine oxide*

A solution of hydrogen peroxide in water (30%, 5.0 equiv.) was added slowly to a stirred solution of phosphine (1.0 equiv., typical scale – 0.5g) in DCM. The reaction mixture was stirred at ambient temperature overnight. The resulting mixture was then extracted with DCM and $NaHCO_3$ solution to remove residual H_2O_2. The combined organic phases were dried over anhydrous $MgSO_4$ and filtered. The solvent was then evaporated under reduced pressure to afford the desired phosphine oxides.

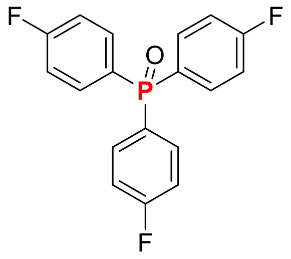

1b

Tris(4-fluorophenyl)phosphine oxide: the product was isolated as a white solid (96% yield, 504 mg). **^{1}H NMR** (400 MHz, Acetonitrile-*d*3) δ [ppm] = 7.64 (dddd, *J* = 11.5, 8.4, 5.2, 2.4 Hz, 1H), 7.23 (td, *J* = 8.8, 2.0 Hz, 1H). **^{13}C NMR** (101 MHz, Acetonitrile-*d*3) δ [ppm] = 165.2 (dd, *J* = 251.3, 3.2 Hz), 134.72 – 134.38 (m), 129.1 (dd, *J* = 107.2, 3.2 Hz), 116.0 (dd, *J* = 21.7, 13.3 Hz). **^{31}P NMR** (162 MHz, Acetonitrile-*d*3) δ [ppm] = 24.79. **^{19}F NMR** (376 MHz, Acetonitrile-*d*3) δ [ppm] = −108.59 (m). The chemical shifts match the literature values.[1]

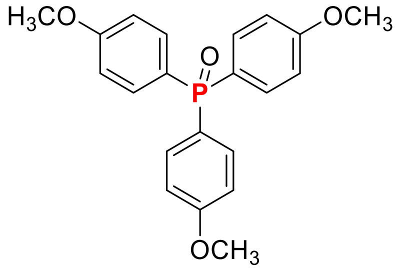

1c

Tris(4-methoxyphenyl)phosphine oxide: the product was isolated as a white solid (95% yield, 523 mg). **^{1}H NMR** (400 MHz, Acetonitrile-*d*3) δ [ppm] = 7.49 (dd, *J* = 11.4, 8.8 Hz, 2H), 6.99 (dd, *J* = 8.9, 2.2 Hz, 2H), 3.79 (s, 3H). **^{13}C NMR** (101 MHz, Acetonitrile-*d*3) δ [ppm] = 162.6 (d, *J* = 2.7 Hz), 133.6 (d, *J* = 11.2 Hz), 124.6 (d, *J* = 110.6 Hz), 114.2 (d, *J* = 12.9 Hz), 55.3. **^{31}P NMR** (162 MHz, Acetonitrile-*d*3) δ [ppm] = 27.6. The chemical shifts match the literature values.[1]

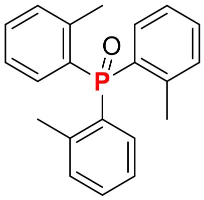

1e

Tri-*o*-tolylphosphine oxide: the product was isolated as a white solid (94% yield, 494 mg). **^{1}H NMR** (400 MHz, Acetonitrile-d_3) δ [ppm] = 7.47 (tt, J = 7.4, 1.6 Hz, 1H), 7.38 – 7.32 (m, 1H), 7.23 – 7.13 (m, 1H), 7.01 (ddd, J = 14.0, 7.7, 1.6 Hz, 1H), 2.39 (s, 3H). **^{13}C NMR** (101 MHz, Acetonitrile-d_3) δ [ppm] = 143.1 (d, J = 7.7 Hz), 132.7 (d, J = 12.6 Hz), 132.20 – 131.93 (m), 131.0 (d, J = 100.8 Hz), 125.8 (d, J = 12.6 Hz), 21.0 (d, J = 4.0 Hz). **^{31}P NMR** (162 MHz, Acetonitrile-d_3) δ [ppm] = 36.59. The chemical shifts match the literature values.[1]

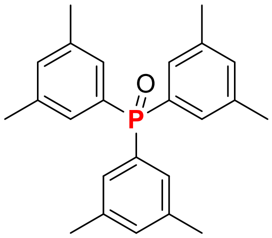

1f

Tris(3,5-dimethylphenyl)phosphine oxide: the product was isolated as a white solid (85% yield, 445 mg). **^{1}H NMR** (400 MHz, Acetonitrile-d_3) δ [ppm] = 7.23 – 7.19 (m, 3H), 2.28 (s, 6H). **^{13}C NMR** (101 MHz, Acetonitrile-d_3) δ [ppm] = 138.5 (d, J = 12.5 Hz), 133.4 (d, J = 102.1 Hz), 133.4 (d, J = 2.9 Hz), 129.2 (d, J = 9.6 Hz), 20.4. **^{31}P NMR** (162 MHz, Acetonitrile-d_3) δ [ppm] = 26.80. The chemical shifts match the literature values.[2]

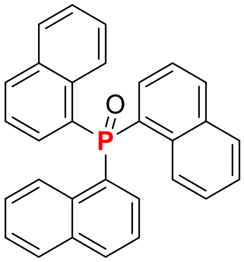

1g

Tri(naphthalen-1-yl)phosphine oxide: the product was isolated as a white solid (89% yield, 462 mg). **^{1}H NMR** (400 MHz, Acetonitrile-d_3) δ [ppm] = 8.80 (dt, J = 8.6, 1.0 Hz, 1H), 8.12 (d, J = 8.5 Hz, 1H), 8.01 (dt, J = 8.1, 1.7 Hz, 1H), 7.56 (ddd, J = 8.1, 6.9, 1.3 Hz, 1H), 7.46 (ddd, J = 8.5, 7.0, 1.5 Hz, 1H), 7.34 (ddd, J = 8.2, 7.1, 2.5 Hz, 1H), 7.22 (ddd, J = 16.0, 7.2, 1.4 Hz, 1H). **^{31}P NMR** (162 MHz, Acetonitrile-d_3) δ [ppm] = 39.11. The chemical shifts match the literature values.[3]

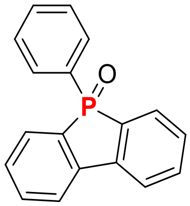

1j

5-phenylbenzo[*b*]phosphindole 5-oxide: the product was isolated as a white solid (97% yield, 514 mg). **^{1}H NMR** (400 MHz, Acetonitrile-*d*3) δ [ppm] = 7.95 (dd, *J* = 7.4, 3.0 Hz, 2H), 7.68 – 7.60 (m, 4H), 7.58 – 7.47 (m, 3H), 7.41 (m, 4H). **^{13}C NMR** (101 MHz, Acetonitrile-*d*3) δ [ppm] = 141.6 (d, *J* = 21.7 Hz), 133.7 (d, *J* = 2.2 Hz), 132.6, 132.4 (d, *J* = 2.9 Hz), 131.7 (d, *J* = 102.6 Hz), 130.7 (d, *J* = 10.6 Hz), 129.8 (d, *J* = 11.1 Hz), 129.4 (d, *J* = 9.6 Hz), 129.0 (d, *J* = 12.5 Hz), 121.8 (d, *J* = 10.1 Hz). **^{31}P NMR** (162 MHz, Acetonitrile-*d*3) δ [ppm] = 32.05. The chemical shift matches the literature values.[4]

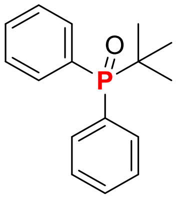

1k

Tert-butyldiphenylphosphine oxide: the product was isolated as a white solid (88% yield, 469 mg). **^{1}H NMR** (400 MHz, Acetonitrile-*d*3) δ [ppm] = 7.95 – 7.87 (m, 4H), 7.62 – 7.42 (m, 6H), 1.15 (d, *J* = 15.0 Hz, 9H). **^{13}C NMR** (101 MHz, Acetonitrile-*d*3) δ [ppm] = 132.1 (d, *J* = 8.2 Hz), 131.8 (d, *J* = 2.9 Hz), 131.2 (d, *J* = 90.4 Hz), 128.5 (d, *J* = 10.6 Hz), 33.5 (d, *J* = 71.3 Hz), 24.4. **^{31}P NMR** (162 MHz, Acetonitrile-*d*3) δ [ppm] = 38.87. The chemical shift matches the literature values.[5]

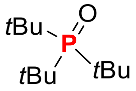

1p

Tri-*tert*-butylphosphine oxide: the product was isolated as a white solid (94% yield, 506 mg). **^{1}H NMR** (400 MHz, Acetonitrile-*d*3) δ [ppm] = 1.33 (d, *J* = 13.0 Hz, 27H). **^{31}P NMR** (162 MHz, Acetonitrile-*d*3) δ [ppm] = 65.16. The chemical shift matches the literature values.[6]

1.3 Preparation of Halo-phosphonium salts

- Preparation of chlorotriphenylphosphonium chloride

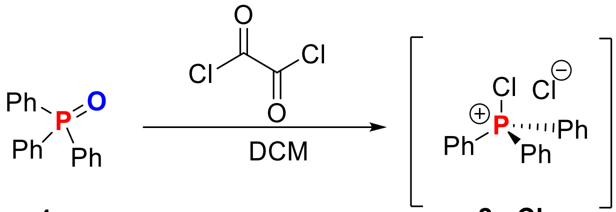

Scheme S2: *Preparation of chlorotriphenylphosphonium chloride*

In the glove box, oxalyl chloride (1.5 equiv., 4.1 mmol, 0. 35 mL) was added dropwise to the stirred solution of triphenylphosphine(V) oxide (1.0 equiv., 2.7 mmol, 0.76 g) in DCM (1 mL) at room temperature. The reaction results in rapid evolution of carbon dioxide and carbon monoxide. The reaction was allowed to stir for 1 hours. The solvent and residual oxalyl chloride was then removed under reduced pressure to afford the product as the white solid.

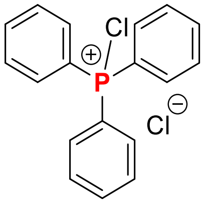

Chlorotriphenylphosphonium chloride: the product was isolated as a white solid. **^{1}H NMR** (400 MHz, Acetonitrile-d_3) δ [ppm] = 7.98 – 7.93 (m, 1H), 7.85 – 7.74 (m, 4H). **^{13}C NMR** (101 MHz, Acetonitrile-D_3) δ [ppm] = 136.9 (d, *J* = 3.4 Hz), 133.8 (d, *J* = 13.0 Hz), 130.6 (d, *J* = 14.9 Hz), 120.2 (d, *J* = 96.3 Hz). **^{31}P NMR** (162 MHz, Acetonitrile-D_3) δ [ppm] = 59.74. The chemical shift matches the literature values.[7]

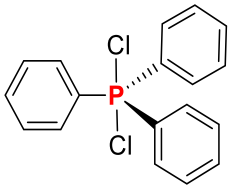

Dichlorotriphenyl-λ^5-phosphane: **^{1}H NMR** (400 MHz, Tetrahydrofuran-d_8) δ [ppm] = 8.12 – 7.85 (m, 2H), 7.58 – 7.41 (m, 3H). **^{13}C NMR** (101 MHz, Tetrahydrofuran-D_8) δ [ppm] = 141.0 (d, *J* = 145.5 Hz), 130.4 (d, *J* = 3.9 Hz), 129.8 (d, *J* = 12.5 Hz), 127.9 (d, *J* = 18.3 Hz). **^{31}P NMR** (162 MHz, Tetrahydrofuran-D_8) δ [ppm] = –45.44.

Dichlorotriphenyl-λ^5-phosphane: **^{1}H NMR** (400 MHz, Benzene-d_6) δ [ppm] = 8.04 (ddd, *J* = 18.0, 8.0, 1.7 Hz, 2H), 7.00 – 6.92 (m, 3H). **^{13}C NMR** (101 MHz, Benzene-D_6) δ [ppm] = 141.3 (d, *J* = 145.0 Hz), 130.3 (d, *J* = 4.3 Hz), 130.1 (d, *J* = 12.5 Hz), 128.0 (d, *J* = 14.4 Hz). **^{31}P NMR** (162 MHz, Benzene-D_6) δ [ppm] = –45.15.[8]

- Preparation of bromotriphenylphosphonium bromide

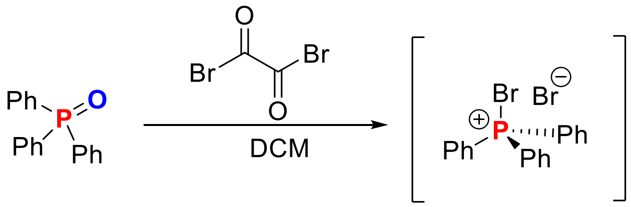

1a **2a-Br**

Scheme S3: *Preparation of bromotriphenylphosphonium bromide*

In the glove box, oxalyl bromide (1.1 equiv., 2.1 mmol, 0.3 mL) was added dropwise to the stirred solution of triphenylphosphine(V) oxide (1.0 equiv., 1.94 mmol, 0.54 g) in DCM (1 mL) at room temperature. The reaction results in rapid evolution of carbon dioxide and carbon monoxide. The reaction was allowed to stir for 1 hours. The solvent and residual oxalyl bromide was then removed under reduced pressure to afford the product as the slightly yellow solid.

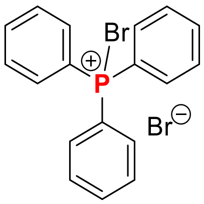

Bromotriphenylphosphonium bromide: **^{1}H NMR** (400 MHz, Acetonitrile-d_3) δ [ppm] = 7.96 – 7.91 (m, 1H), 7.78 – 7.74 (m, 4H). **^{13}C NMR** (101 MHz, Acetonitrile-d_3) δ 136.9 (d, J = 3.4 Hz), 134.1 (d, J = 13.0 Hz), 130.6 (d, J = 14.4 Hz), 119.6 (d, J = 86.2 Hz). **^{31}P NMR** (162 MHz, Acetonitrile-d_3) δ [ppm] = 50.96. The chemical shift matches the literature values.[9]

- Preparation of Chlorotriphenylphosphonium bromide

Solution of **2a-Cl** in ACN-D_3 (0.0166 g, 0.05 mmol) was added to a solution of **2a-Br** (0.0211 g, 0.05 mmol) in ACN-D_3 via a syringe.

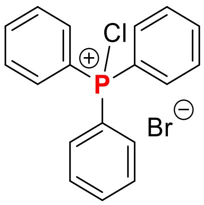

Chlorotriphenylphosphonium bromide: **^{1}H NMR** (400 MHz, Acetonitrile-d_3) δ [ppm] = 8.00 – 7.95 (m, 1H), 7.84 – 7.75 (m, 4H). **^{31}P NMR** (162 MHz, Acetonitrile-d_3) δ [ppm] = 64.63. The chemical shift matches the literature values.[9]

- Preparation of $[Ph_3PCl]^+OTf^-$

To the ACN-D_3 solution of **2a-Cl** (0.0332 g, 0.1 mmol), TMSOTf (0.0222 g, 0.1 mmol) was added. The reaction was allowed to stirred for 15 minutes.

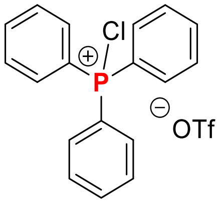

Chlorotriphenylphosphonium trifluoromethanesulfate: **^{31}P NMR** (162 MHz, Acetonitrile-d_3) δ [ppm] = 66.19. **^{19}F NMR** (376 MHz, Acetonitrile-d_3) δ [ppm] = −79.16. The chemical shift matches the literature values.[7]

- Preparation of $[Ph_3PBr]^+OTf^-$

To the ACN-D_3 solution of $[Ph_3PBr]^+Br^-$ (0.0422 g, 0.1 mmol), TMSOTf (0.0222 g, 0.1 mmol) was added. The reaction was allowed to stirred for 15 minutes.

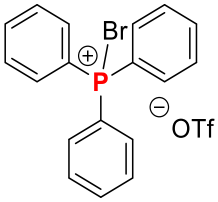

Bromotriphenylphosphonium trifluoromethanesulfate: **^{1}H NMR** (400 MHz, Acetonitrile-d_3) δ [ppm] = 7.97 – 7.89 (m, 1H), 7.80 – 7.73 (m, 4H). **^{31}P NMR** (162 MHz, Acetonitrile-d_3) δ [ppm] = 51.88.

- Preparation of $[Ph_3POTMS]^+OTf^-$

To the ACN-D_3 solution of triphenylphosphine oxide (0.0278 g, 0.1 mmol), TMSOTf (0.0222 g, 0.1 mmol) was added. The reaction was allowed to stirred for 15 minutes.

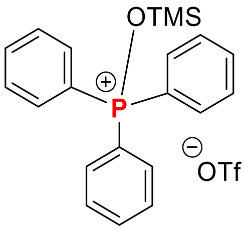

Triphenyl((trimethylsilyl)oxy)phosphonium trifluoromethanesulfonate: **^{1}H NMR** (400 MHz, Acetonitrile-d_3) δ [ppm] = 7.92 – 7.87 (m, 3H), 7.82 – 7.66 (m, 12H), 0.31 (br, 9H). **^{31}P NMR** (162 MHz, Acetonitrile-d_3) δ [ppm] = 52.71.[10]

2. Reaction optimization

Table S1. Optimization of the reaction conditions. [a]

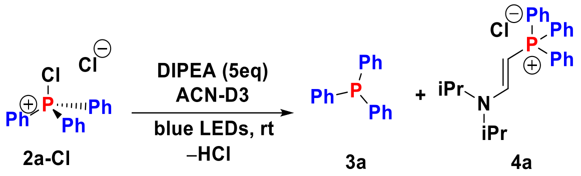

Entry	Variation from standard conditions	Time	Yields **3a**[b] (%)	Yields **4a**[b] (%)
1	None	3 days	22 (26)	30
2	DCM-D_2 instead of ACN-D_3	3 days	(29)	25
3	Other solvents (THF-D_8, C_6D_6)	3 days	trace	-
4	Et_3N instead of DIPEA	3 days	20 (19)	30[c]
5	Lutidine instead of DIPEA	3 days	n.r	n.r
6	DABCO instead of DIPEA	3 days	38 (38)	-
7	(*n*Pr)$_3$N instead of DIPEA	3 days	23	-
8	$[Ph_3PBr]^+Br^-$ instead of 2a-Cl	1 day	62 (59)	32
9	$[Ph_3PCl]^+Br^-$ instead of 2a-Cl	1 day	38 (40)	33
10	$[Ph_3PCl]^+OTf^-$ instead of 2a-Cl	1 day	29 (30)	29
11	$[Ph_3PBr]^+OTf^-$ instead of 2a-Cl	1 day	40 (42)	17
12	$[Ph_3POTMS]^+OTf^-$ instead of 2a-Cl	3 days	n.r	n.r
13	$[Ph_3PBr]^+Br^-$ and (*n*Pr)$_3$N instead of 2a-Cl and DIPEA	1 day	70	-
14	Without visible light irradiation	1 day	n.r	n.r
15	Without visible light irradiation, 80ºC	1 day	n.r	n.r

[a] Standard conditions: 0.1 mmol of **2a-Cl** (1 equiv.), 0.5 mmol of donor (5 equiv.), solvent (0.5 mL), blue light. DIPEA: N,N-di-iso-propylethylamine, lutidine: 2,6-dimethylpyridine, DABCO: 1,4-diazabicyclo[2.2.2]octane. [b] ^{1}H NMR yields of crude reaction mixtures using cyclohexene as an internal standard. The value within parentheses refers to yields of isolated product. [c] Reflecting the structure of the amine, this product contains Et instead of *i*Pr substituents at **4a**.

3. General procedure for conversion of phosphine(V) oxides to phosphines via chloro/bromophosphonium salts

In the glove box, the phosphine oxide (0.10 mmol, 1.0 equiv.) was dissolved under stirring in a small vial of DCM (0.5 mL). Oxalyl chloride/ bromide (0.15 mmol, 1.5 equiv.) was added dropwise to the stirred solution at r.t. over 30 min. The reaction results in rapid evolution of carbon dioxide and carbon monoxide. The solvent and residual oxalyl chloride/bromide were then removed under reduced pressure to afford the halophosphonium salts. The obtained halophosphonium salts were then dissolved in ACN-D_3 (0.5 ml), transferred to Young-type NMR tube (borosilicate glass), and electron donor (0.5 mmol, 5.0 equiv.) was added. After all components were added, the NMR tube was closed and taken out of the glove box. A Kesill Tuna Blue lamp was used for irradiation (wavelength 460 nm, A160WE Tuna Blue light source, Power supply: 100-240 V AC (input), 19-24V DC (output), https://www.marine-aquatics.eu/en/kessil-a160we-tuna-blue-led-lighting-40w). The NMR tubes were placed on a laboratory shaker, directly exposed to the lamp (see the picture in Fig. S1) at a distance of 5 cm. No optical filters were used at any point.

^{31}P NMR and ^{1}H NMR measurements were systematically performed to follow the reactions. NMR yields of crude reaction mixtures were determined using cyclohexene as an internal standard**.** Purification by filtering the reaction mixture through a small pad of silica gel using DCM afforded the desired products.

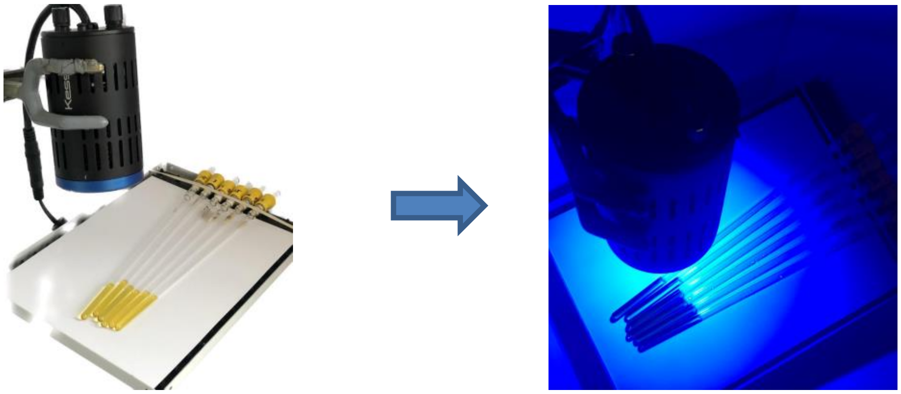

Fig. S1. *Irradiation set-up with laboratory shaker and Kessil Tuna Blue lamp.*

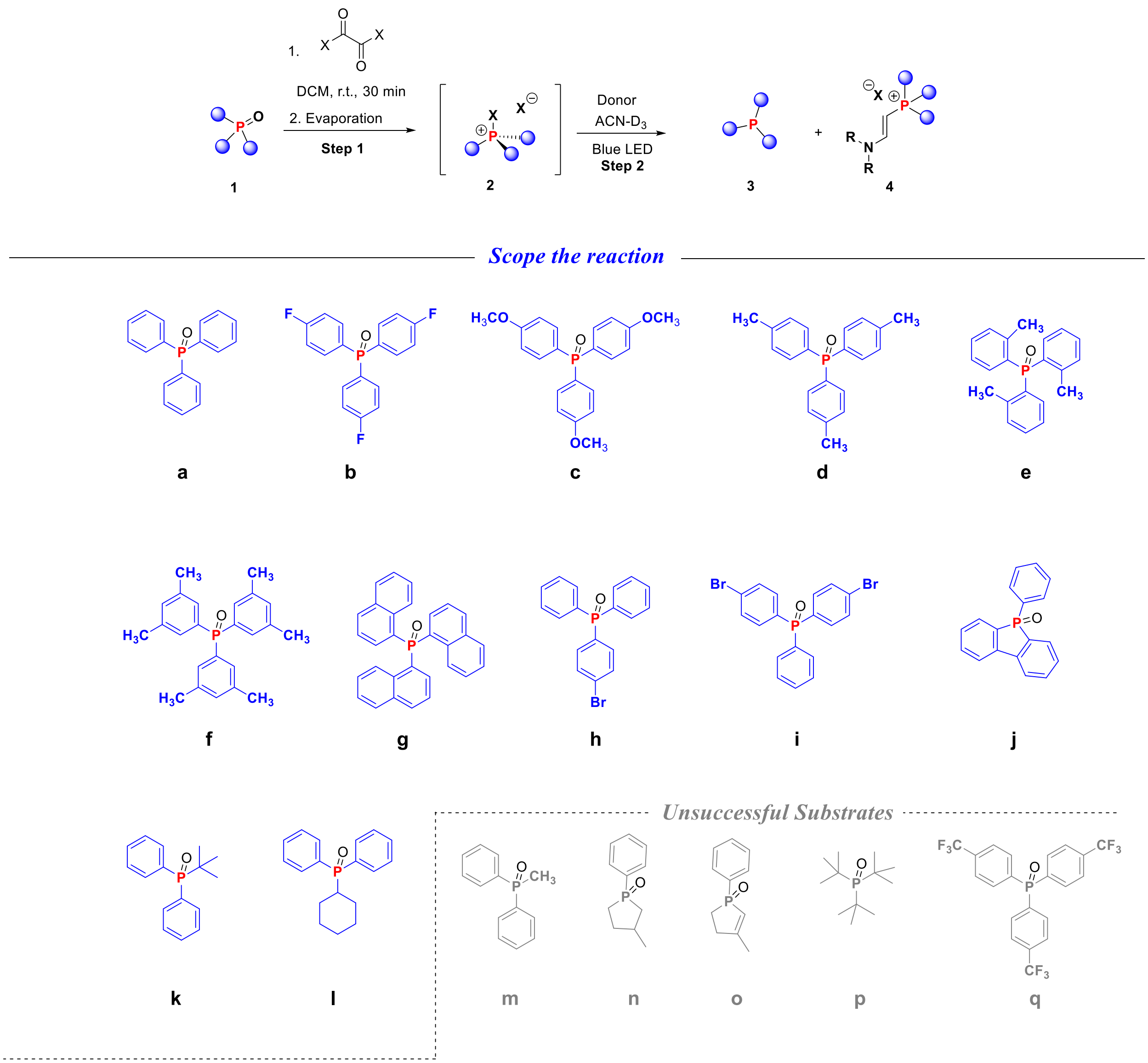

Scheme S4: *Substrate scope for the one-pot activation/reduction of phosphine oxides to phosphines via phosphonium salts. Standard conditions: substrates (1.0 equiv.), electron donors (5.0 equiv.), solvents (0.5 mL), blue light.*

Table S2: *Reaction details on one-pot reduction of phosphine oxides to phosphines*

Entry	Substrate **1**	m (mg) of **1**	Product **2** (Step 1)	^{31}P ppm	Donor	Product (Step 2)	^{31}P ppm	Time	Yield (%)	Yield (mg)
1	**1a**	27.8	**2a-Br**	51.0 (s)	**(*n*Pr)$_3$N**	**3a**	–5.4 (s)	1 days	70 (70)	18.3
2	**1b**	24.5	**2b-Cl**	48.0 (s)	**DIPEA**	**3b**<br>**4b**	–8.9 (s)<br>21.7 (s)	1 day	31	
3	**1b**	31.8	**2b-Br**	44.4 (s)	**DIPEA**	**3b**	–8.9 (s)	1.5h	77 (77)	23.3
4	**1b**	33.2	**2b-Br**	46.4 (s)	**(*n*Pr)$_3$N**	**3b**	–8.9 (s)	3h	80 (77)	24.3
5	**1c**	36.8	**2c-Cl**	64.5 (s)	**DIPEA**	**3c**<br>**4c**	–10.2 (s)<br>20.5 (s)	5 days	42 (39)<br>38 (39)	13.7<br>20.0
6	**1c**	36.8	**2c-Br**	49.6 (s)	**DIPEA**	**4c**	20.5 (s)	4 days	41	
7	**1c**	36.8	**2c-Br**	49.6 (s)	**(*n*Pr)$_3$N**	**3c**	–10.2 (s)	4 days	45 (47)	16.5
8	**1d**	36.6	**2d-Cl**	65.5 (s)	**DIPEA**	**3d**<br>**4d**	–7.89 (s)<br>21.8 (s)	6 days	30 (43)<br>(43)	15.0<br>22.9
9	**1d**	28.7	**2d-Br**	50.5 (s)	**DIPEA**	**4d**	21.8 (s)	1 day	22 (31)	14.2
10	**1d**	32.0	**2d-Br**	50.5 (s)	**(*n*Pr)$_3$N**	**3d**	7.90 (s)	4 days	54 (62)	18.9
11	**1f**	36.2	**2f-Cl**	66.1 (s)	**DIPEA**	**3f**<br>**4f**	–5.0 (s)<br>22.4 (s)	5 days	39 (36)<br>24 (39)	12.5<br>19.8
12	**1f**	36.2	**2f-Br**	52.2 (s)	**DIPEA**	**3f**<br>**4f**	–5.0 (s)<br>22.4 (s)	4 days	18 (14)<br>30 (33)	4.8<br>16.7

13	**1f**	36.2	**2f-Br**	51.6 (s)	**(*n*Pr)$_3$N**	**3f**	–5.0 (s)	3 days	52 (50)	17.3
14	**1e**	25.0	**2e-Cl**	64.7 (s)	**DIPEA**	**3e**	–30.6 (s)	5 days	56	
15	**1e**	26.2	**2e-Cl**	64.7 (s)	**DABCO**	**3e**	–30.6 (s)	5 days	59	
16	**1e**	29.8	**2e-Br**	46.0 (s)	**DIPEA**	**3e**	–30.6 (s)	1 day	57 (56)	15.9
17	**1e**	32.0	**2e-Br**	44.8 (s)	**(*n*Pr)$_3$N**	**3e**	–30.4 (s)	1 day	70 (81)	24.7
18	**1h**	35.7	**2h-Br**	51.3 (s)	**(*n*Pr)$_3$N**	**3h**	–6.4 (s)	1 days	50 (53)	18.1
19	**1i**	43.6	**2i-Br**	50.7 (s)	**(*n*Pr)$_3$N**	**3i**	–7.33 (s)	1 days	40 (45)	18.9
20	**1g**	21.4	**2g-Cl**	62.3 (s)	**(*n*Pr)$_3$N**	**3g**	–33.5 (s)	2 days	50 (51)	10.5
21	**1j**	27.6	**2j-Br**	37.8 (s)	**(*n*Pr)$_3$N**	**3j**	–10.1 (s)	1 day	48 (47)	12.2
22	**1k**	25.8	**2k-Cl**	86.8 (s)	**(*n*Pr)$_3$N**	**3k**	17.8 (s)	8 days	55 (52)	12.6
23	**1l**	28.4	**2l-Br**	68.7 (s)	**(*n*Pr)$_3$N**	**3l**	–4.2 (s)	4 days	67 (67)	18.0
24	**1m**	21.6	**2m-Cl**	61.6 (s)[a]	**DIPEA**	**-**	-	-	-	
25	**1n**	19.4	**2n-Cl**	80.6 (s)	**DIPEA**	**-**	-	-	-	
26	**1o**	19.2	**2o-Br**	79.1 (s)	**(*n*Pr)$_3$N**	**-**	-	-	-	
27	**1p**	21.8	**n.r**	-	**-**	**-**	-	-	-	
28	**1q**	48.2	**n.r**	-	**-**	**-**	-	-	-	

Yields refer to NMR yields of crude reaction mixtures using cyclohexene as internal standard. The yields in brackets are isolated yields. In these cases, the yields are given in % and mg. n.r. : no reaction. [a]: reaction performed in DCM-d_2.

4. Comparison of the developed methodology with literature procedures

The developed methodology for the overall reduction of phosphine oxides to phosphines can be compared to known literature procedures. In general, the developed methodology is the first of its kind to report a photochemical reduction. Activation of the $R_3P{=}O$ is done with $(COCl)_2$, or $(COBr)_2$, in analogy to some of the procedures below. Once the corresponding halophosphonium salt is formed, the methodology requires an amine (for EDA complex formation and as electron donor) and light for R_3P formation. Reactive reagents such as silanes or metal hydrides are omitted.

Reference	Chemistry/reaction	Yields	Comments
Nocera, *JACS* **2018**, *140*, 13711-13718	$Ph_3P{=}O$ to Ph_3P in presence of $B(OR)_3$	37%	Electrochemical, side product Ph_2PH 40%, NMR scale
Tanaka, *Synthesis* **2011** 24 4091-4098	$Ph_3P{=}O$ to Ph_3PCl_2 with $(COCl)_2$, Ph_3PCl_2 to Ph_3P in the presence of TMSCl	58-72%	Two steps, electrochemical, mmol scale
Tanaka, *Tet. Lett* **2010** *51* 698	Ph_3PHal_2 to Ph_3P in the presence of $AlHal_3$	40-84%	Two step, e-chem, mmol scale
Stephan and Grimme *Angew. Chem. Int. Ed.* **2018** 15253	$Ph_3P{=}O$ to Ph_3PCl_2 with $(COCl)_2$, Ph_3PCl_2 to TPP by H_2	93%	Two step, Chemical, 130 °C, 48h, 80 bar H_2
Org. Lett. **2001** 87	$Ph_3P{=}O$ to Ph_3POMe^+ with MeOTf, salt to Ph_3P with $LiAlH_4$	97%	Two step, chemical, mg scale
O'Brien, *Angew. Chem. Int. Ed.* **2009** 6836	$R_3P{=}O$ to R_3P by Ph_2SiH_2		Part of the catalytic cycle. Phosphine oxide used - 3-methyl-1-phenylphospholane-1-oxide
Radosevich, *JACS* **2018** *140*, 3103	$R_3P{=}O$ to R_3P by $PhSiH_3$		Part of catalytic cycle. Several phosphine oxides tested, 1,2,2,3,4,4-hexamethylphosphetane oxide gave best results.
Chem. Soc. Rev. **2015** *44,* 2508-2528	$R_3P{=}O$ to R_3P		Chemical reduction, review paper

5. NMR Spectroscopic data

Synthesis of Triphenylphosphine 3a

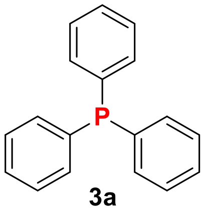

3a

Triphenyl phosphine: the product was isolated as a white solid. **^{1}H NMR** (400 MHz, Acetonitrile-d_3) δ [ppm] = 7.36 – 7.34 (m, 9H), 7.29 – 7.24 (m, 6H). **^{31}P NMR** (162 MHz, Acetonitrile-d_3) δ [ppm] = –5.33. The chemical shift matches the literature values.[11]

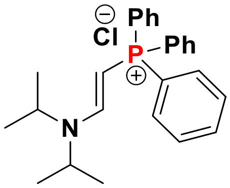

4a

(*E*)-(2-(diisopropylamino)vinyl)triphenylphosphonium chloride: the product was isolated as a white solid. **^{1}H NMR** (400 MHz, Acetonitrile-d_3) δ [ppm] = 7.80 – 7.75 (m), 7.67 – 7.59 (m), 6.44 (dd, J = 16.4, 14.3 Hz, 1H), 4.61 (t, J = 14.5 Hz, 4H), 4.15 (p, J = 6.7 Hz, 1H), 3.61 (p, J = 6.7 Hz, 1H), 1.25 (d, J = 6.7 Hz, 6H), 0.92 (d, J = 6.7 Hz, 6H). **^{13}C NMR** (101 MHz, Chloroform-d) δ [ppm] = 151.2 (d, J = 18.4 Hz), 134.5 (d, J = 2.7 Hz), 133.4 (d, J = 10.3 Hz), 130.2 (d, J = 12.6 Hz), 122.1 (d, J = 92.1 Hz), 59.3 (d, J = 116.7 Hz), 48.7 (d, J = 205.9 Hz), 23.7, 19.8. **^{31}P NMR** (162 MHz, Acetonitrile-d_3) δ [ppm] = 23.2. The chemical shift matches the literature values.[12]

Synthesis of tris(4-fluorophenyl)phosphane 3b

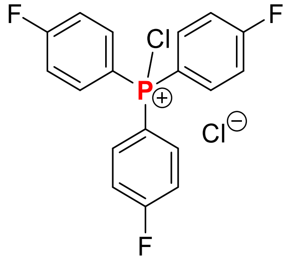

2b-Cl

Chlorotris(4-fluorophenyl)phosphonium chloride: the product was isolated as a white solid. **^{1}H NMR** (400 MHz, Acetonitrile-*d*3) δ [ppm] = 8.02 – 7.85 (m, 2H), 7.52 (td, *J* = 8.8, 3.4 Hz, 2H). **^{13}C NMR** (101 MHz, Acetonitrile-*d*3) δ [ppm] = 167.4 (dd, *J* = 259.1, 3.9 Hz), 137.0 (dd, *J* = 15.9, 10.6 Hz), 118.2 (d, *J* = 110.1, 2.9 Hz), 118.1 (dd, *J* = 23.1, 17.3 Hz). **^{31}P NMR** (162 MHz, Acetonitrile-*d*3) δ [ppm] = 48.03. **^{19}F NMR** (376 MHz, Acetonitrile-*d*3) δ [ppm] = –100.43. The chemical shift matches the literature values.[1]

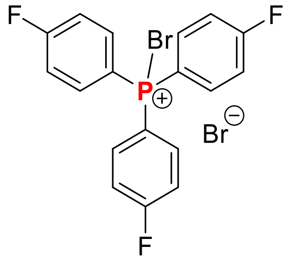

2b-Br

Bromotris(4-fluorophenyl)phosphonium bromide: the product was isolated as a pale yellow solid. **^{1}H NMR** (400 MHz, Acetonitrile-*d*3) δ [ppm] = 7.92 – 7.79 (m, 2H), 7.56 – 7.45 (m, 2H). **^{13}C NMR** (101 MHz, Acetonitrile-*d*3) δ [ppm] = 167.7 (dd, *J* = 259.6, 3.9 Hz), 137.6 (dd, *J* = 15.4, 10.6 Hz), 118.3 (dd, *J* = 22.9, 16.6 Hz), 115.7 (dd, *J* = 92.2, 3.1 Hz). **^{31}P NMR** (162 MHz, Acetonitrile-*d*3) δ [ppm] = 44.36. **^{19}F NMR** (376 MHz, Acetonitrile-*d*3) δ [ppm] = –100.05.

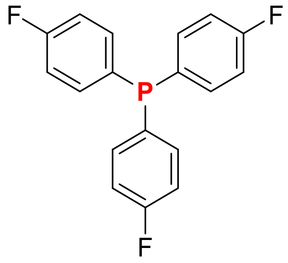

3b

Tris(4-fluorophenyl)phosphane: the product was isolated as a white solid. **^{1}H NMR** (400 MHz, Acetonitrile-*d*3) δ [ppm] = 7.30 – 7.23 (m, 2H), 7.14 – 7.07 (m, 2H). **^{13}C NMR** (101 MHz, Acetonitrile-*d*3) δ [ppm] = 163.5 (d, *J* = 247.6 Hz), 135.6 (dd, *J* = 21.7, 8.2 Hz), 132.9 (dd, *J* = 3.4, 11.1 Hz), 115.9 (dd, *J* = 21.4, 7.5 Hz). **^{31}P NMR** (162 MHz, Acetonitrile-*d*3) δ [ppm] = –8.90 (d,

J = 6.3 Hz). **^{19}F NMR** (376 MHz, Acetonitrile-d_3) δ [ppm] = −113.69 (tq, J = 10.0, 5.3 Hz). The chemical shift matches the literature values.[11]

Synthesis of tris(4-methoxyphenyl)phosphane 3c

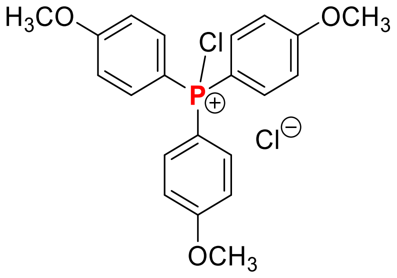

2c-Cl

Chlorotris(4-methoxyphenyl)phosphonium chloride: the product was isolated as a white solid. **^{1}H NMR** (400 MHz, Acetonitrile-d_3) δ [ppm] = 7.74 – 7.62 (m, 2H), 7.29 – 7.22 (m, 2H), 3.92 (s, 3H). **^{13}C NMR** (101 MHz, Acetonitrile-d_3) δ [ppm] = 166.4 (d, J = 2.9 Hz), 136.3 (d, J = 15.1 Hz), 116.3 (d, J = 16.3 Hz), 110.01 (d, J = 105.1 Hz), 56.2. **^{31}P NMR** (162 MHz, Acetonitrile-d_3) δ [ppm] = 64.46. The chemical shift matches the literature values.[1]

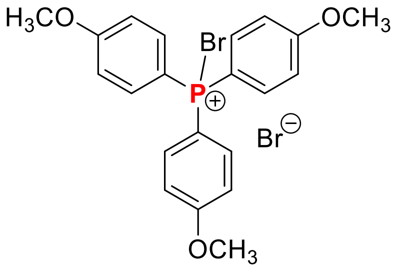

2c-Br

Bromotris(4-methoxyphenyl)phosphonium bromide: the product was isolated as a pale yellow solid. **^{1}H NMR** (400 MHz, Acetonitrile-d_3) δ [ppm] = 7.70 – 7.61 (m, 2H), 7.29 – 7.19 (m, 2H), 3.91 (s, 3H). **^{13}C NMR** (101 MHz, Acetonitrile-d_3) δ [ppm] = 166.2 (d, J = 2.9 Hz), 136.3 (d, J = 14.9 Hz), 116.2 (d, J = 16.4 Hz), 110.4 (d, J = 98.3 Hz), 56.2. **^{31}P NMR** (162 MHz, Acetonitrile-d_3) δ [ppm] = 49.59.

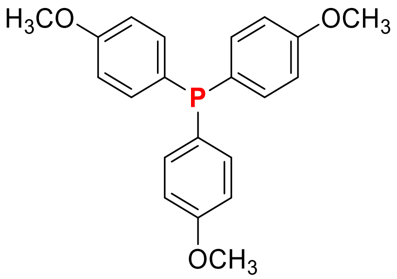

3c

Tris(4-methoxyphenyl)phosphane: the product was isolated as a white solid. **^{1}H NMR** (400 MHz, Acetonitrile-d_3) δ [ppm] = 7.18 – 7.14 (m, 6H), 6.89 (d, J = 8.9 Hz, 6H), 3.75 (s, 3H). **^{13}C NMR** (101 MHz, Acetonitrile-d_3) δ [ppm] = 160.4, 134.8 (d, J = 21.1 Hz), 129.0 (d, J = 8.5 Hz), 114.3

(d, J = 7.8 Hz), 55.0. **^{31}P NMR** (162 MHz, Acetonitrile-d_3) δ [ppm] = −10.18. The chemical shift matches the literature values.[11]

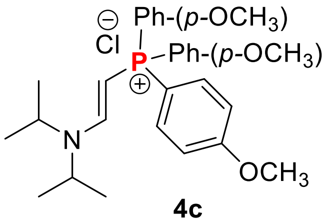

4c

(*E*)-(2-(diisopropylamino)vinyl)tris(4-methoxyphenyl)phosphonium chloride: the product was isolated as a white solid. **^{1}H NMR** (400 MHz, Acetonitrile-d_3) δ [ppm] = 7.50 (dd, J = 12.3, 8.9 Hz, 6H), 7.14 (dd, J = 9.0, 2.5 Hz, 6H), 6.40 (dd, J = 16.6, 14.3 Hz, 1H), 4.62 (t, J = 14.5 Hz, 1H), 4.14 (p, J = 6.8 Hz, 1H), 3.86 (s, 9H), 3.60 (p, J = 6.6 Hz, 1H), 1.23 (d, J = 6.4 Hz, 6H), 0.94 (d, J = 5.8 Hz, 6H). **^{13}C NMR** (101 MHz, Acetonitrile-d_3) δ [ppm] = 164.1 (d, J = 2.9 Hz), 135.4 (d, J = 11.9 Hz), 133.5 (d, J = 11.1 Hz), 115.4 (d, J = 13.8 Hz), 114.1 (d, J = 13.0 Hz), 113.8 (d, J = 99.6 Hz), 60.8 (d, J = 117.9 Hz), 55.7, 48.4 (d, J = 142.8 Hz), 22.7, 18.7. **^{31}P NMR** (162 MHz, Acetonitrile-d_3) δ [ppm] = 20.48.

Synthesis of tri-p-tolylphosphane 3d

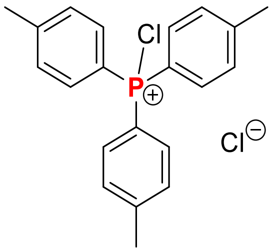

2d-Cl

Chlorotri-*p*-tolylphosphonium chloride: the product was isolated as a white solid. **^{1}H NMR** (400 MHz, Acetonitrile-d_3) δ [ppm] = 7.72 – 7.52 (m, 12H), 2.49 (s, 9H). **^{13}C NMR** (101 MHz, Acetonitrile-d_3) δ [ppm] = 149.2 (d, J = 3.4 Hz), 133.9 (d, J = 14.0 Hz), 131.3 (d, J = 15.4 Hz), 116.1 (d, J = 97.3 Hz), 21.2. **^{31}P NMR** (162 MHz, Acetonitrile-d_3) δ [ppm] = 65.54.

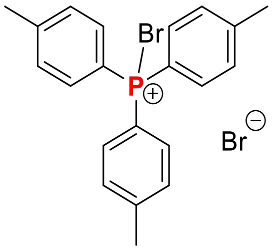

2d-Br

Bromotri-*p*-tolylphosphonium bromide: the product was isolated as a pale yellow solid. **^{1}H NMR** (400 MHz, Acetonitrile-d_3) δ [ppm] = 7.64 – 7.54 (m, 12H), 2.49 (s, 9H). **^{13}C NMR** (101 MHz, Acetonitrile-d_3) δ [ppm] = 148.9 (d, J = 3.4 Hz), 134.0 (d, J = 13.5 Hz), 131.2 (d, J = 14.9 Hz), 116.5 (d, J = 90.5 Hz), 21.1. **^{31}P NMR** (162 MHz, Acetonitrile-d_3) δ [ppm] = 50.54.

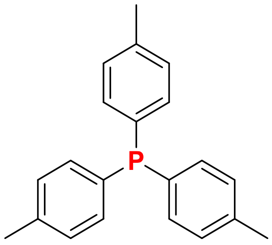

3d

Tri-*p*-tolylphosphane: the product was isolated as a white solid. **^{1}H NMR** (400 MHz, Acetonitrile-d_3) δ [ppm] = 7.17 – 7.11 (m, 12H), 2.30 (s, 9H). **^{13}C NMR** (101 MHz, Acetonitrile-d_3) δ [ppm] = 139.0, 134.3 (d, *J* = 10.1 Hz), 133.5 (d, *J* = 19.7 Hz), 129.4 (d, *J* = 6.7 Hz), 20.4. **^{31}P NMR** (162 MHz, Acetonitrile-d_3) δ [ppm] = −7.89. The chemical shift matches the literature values.[11]

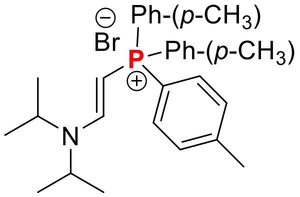

4d

(*E*)-(2-(diisopropylamino)vinyl)tri-p-tolylphosphonium bromide: the product was isolated as a white solid. **^{1}H NMR** (400 MHz, Acetonitrile-d_3) δ [ppm] = 7.50 – 7.41 (m, 12H), 6.42 (dd, *J* = 16.5, 14.3 Hz, 1H), 4.58 (t, *J* = 14.5 Hz, 1H), 4.13 (p, *J* = 6.5 Hz, 1H), 3.60 (p, *J* = 6.6 Hz, 1H), 2.43 (s, 9H), 1.24 (d, *J* = 6.7 Hz, 6H), 0.93 (d, *J* = 6.7 Hz, 6H). **^{13}C NMR** (101 MHz, Acetonitrile-d_3) δ [ppm] = 145.4, 133.4 (d, *J* = 10.6 Hz), 131.7 (d, *J* = 10.1 Hz), 130.5 (d, *J* = 13.0 Hz), 129.3 (d, *J* = 12.5 Hz), 119.6 (d, *J* = 94.4 Hz), 59.4 (d, *J* = 117.5 Hz), 48.5 (d, *J* = 131.7 Hz), 22.6, 20.7 (d, *J* = 14.0 Hz), 18.6. **^{31}P NMR** (162 MHz, Acetonitrile-d_3) δ [ppm] = 21.70.

Synthesis of tri-o-tolylphosphane 3e

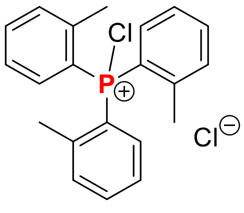

2e-Cl

Chlorotri-*o*-tolylphosphonium chloride: the product was isolated as white solid. **^{1}H NMR** (400 MHz, Acetonitrile-*d*3) δ [ppm] = 7.88 (t, *J* = 7.7 Hz, 1H), 7.68 (t, *J* = 7.2 Hz, 1H), 7.57 – 7.37 (m, 2H), 2.37 (s, 3H). **^{13}C NMR** (101 MHz, Acetonitrile-*d*3) δ [ppm] = 144.7 (d, *J* = 9.8 Hz), 137.3 (d, *J* = 3.0 Hz), 135.5 (d, *J* = 16.8 Hz), 134.5 (d, *J* = 12.4 Hz), 128.0 (d, *J* = 15.5 Hz), 116.3, 21.9 (d, *J* = 5.2 Hz). **^{31}P NMR** (162 MHz, Acetonitrile-*d*3) δ [ppm] = 64.7 ppm. The chemical shift matches the literature values.[13]

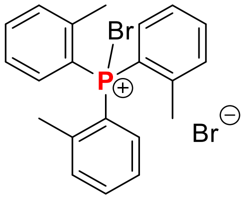

2e-Br

Bromotri-*o*-tolylphosphonium bromide: the product was isolated as a pale yellow solid. **^{1}H NMR** (400 MHz, Acetonitrile-*d*3) δ [ppm] = 7.84 (t, *J* = 7.2 Hz, 1H), 7.64 (t, *J* = 7.3 Hz, 2H), 7.54 – 7.42 (m, 3H), 2.35 (s, 5H). **^{13}C NMR** (101 MHz, Acetonitrile-*d*3) δ [ppm] = 144.6 (d, *J* = 10.1 Hz), 137.0 (d, *J* = 3.4 Hz), 135.6 (d, *J* = 15.9 Hz), 134.6 (d, *J* = 12.0 Hz), 128.1 (d, *J* = 14.9 Hz), 116.6 (d, *J* = 82.4 Hz), 22.2 (d, *J* = 5.3 Hz). **^{31}P NMR** (162 MHz, Acetonitrile-*d*3) δ [ppm] = 45.99. The chemical shift matches the literature values.[13]

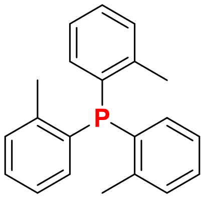

3e

Tri-*o*-tolylphosphane: the product was isolated as white solid. **^{1}H NMR** (400 MHz, Acetonitrile-*d*3) δ [ppm] = 7.32 – 7.19 (m, 6H), 7.14 – 6.98 (m, 3H), 6.65 (dd, *J* = 7.3, 3.1 Hz, 3H), 2.32 (d, *J* = 1.6 Hz, 9H). **^{13}C NMR** (101 MHz, Acetonitrile-*d*3) δ [ppm] = 142.6 (d, *J* = 26.5 Hz), 134.3 (d, *J* = 10.6 Hz), 132.8, 130.2 (d, *J* = 4.8 Hz), 129.0, 126.3, 20.3 (d, *J* = 21.7 Hz). **^{31}P NMR** (162 MHz, Acetonitrile-*d*3) δ [ppm] = –30.55. The chemical shift matches the literature values.[2]

Synthesis of tris(3,5-dimethylphenyl)phosphane 3f

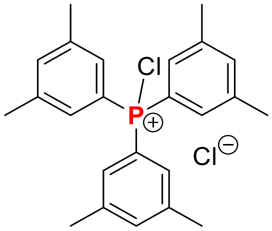

2f-Cl

Chlorotris(3,5-dimethylphenyl)phosphonium chloride: the product was isolated as white solid. **^{1}H NMR** (400 MHz, Acetonitrile-d_3) δ [ppm] = 7.58 (s, 1H), 7.38 (d, *J* = 15.9 Hz, 2H), 2.36 (s, 6H). **^{13}C NMR** (101 MHz, Acetonitrile-d_3) δ [ppm] = 141.0 (d, *J* = 15.9 Hz), 138.6, 131.1 (d, *J* = 13.0 Hz), 119.3 (d, *J* = 92.5 Hz), 20.3. **^{31}P NMR** (162 MHz, Acetonitrile-d_3) δ [ppm] = 66.09.

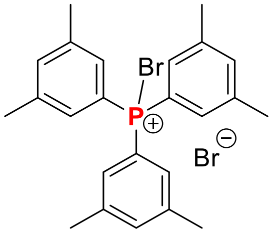

2f-Br

Bromotris(3,5-dimethylphenyl)phosphonium bromide: the product was isolated as pale yellow solid. **^{1}H NMR** (400 MHz, Acetonitrile-d_3) δ [ppm] = 7.54 (s, 1H), 7.33 (d, *J* = 16.9 Hz, 2H), 2.36 (s, 6H). **^{13}C NMR** (101 MHz, Acetonitrile-d_3) δ [ppm] = 141.0 (d, *J* = 15.4 Hz), 138.3 (d, *J* = 3.9 Hz), 131.3 (d, *J* = 12.5 Hz), 119.6 (d, *J* = 84.8 Hz), 20.3. **^{31}P NMR** (162 MHz, Acetonitrile-d_3) δ [ppm] = 52.19.

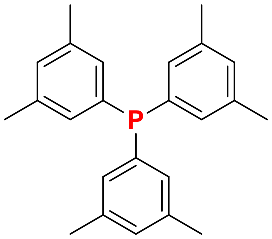

3f

Tris(3,5-dimethylphenyl)phosphane: the product was isolated as white solid. **^{1}H NMR** (400 MHz, Acetonitrile-d_3) δ [ppm] = 6.99 (s, 1H), 6.88 (d, *J* = 8.1 Hz, 2H), 2.21 (s, 6H). **^{13}C NMR** (101 MHz, Acetonitrile-d_3) δ [ppm] = 138.1 (d, *J* = 7.5 Hz), 137.3 (d, *J* = 10.6 Hz), 131.2 (d, *J* = 19.9 Hz), 130.4, 20.4. **^{31}P NMR** (162 MHz, Acetonitrile-d_3) δ [ppm] = –4.94. The chemical shift matches the literature values.[2]

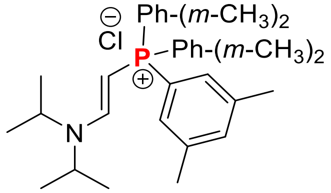

4f

(*E*)-(2-(diisopropylamino)vinyl)tris(3,5-dimethylphenyl)phosphonium chloride: the product was isolated as white solid. **^{1}H NMR** (400 MHz, Acetonitrile-*d*$_3$) δ [ppm] = 7.39 (d, *J* = 0.8, 3H), 7.19 (dd, *J* = 13.4, 0.8 Hz, 6H), 6.41 (dd, *J* = 16.2, 14.4 Hz, 1H), 4.56 (t, *J* = 14.5 Hz, 1H), 4.16 (p, *J* = 6.8 Hz, 1H), 3.61 (p, *J* = 6.2 Hz, 1H), 2.32 (s, 18H), 1.24 (d, *J* = 6.6 Hz, 6H), 0.93 (d, *J* = 6.7 Hz, 6H). **^{13}C NMR** (101 MHz, Acetonitrile-*d*$_3$) δ [ppm] = 139.9 (d, *J* = 13.3 Hz), 135.5 (d, *J* = 3.2 Hz), 133.4 (d, *J* = 2.8 Hz), 130.9 (d, *J* = 10.2 Hz), 129.2 (d, *J* = 9.5 Hz), 122.7 (d, *J* = 90.9 Hz), 59.2 (d, *J* = 116.6 Hz), 48.3 (d, *J* = 166.5 Hz), 22.7, 20.4, 18.7. **^{31}P NMR** (162 MHz, Acetonitrile-*d*$_3$) δ [ppm] = 22.34.

Synthesis of tri(naphthalen-1-yl)phosphane 3g

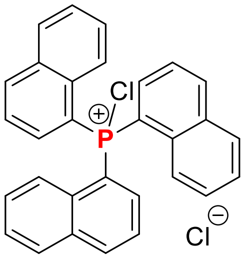

2g-Cl

Chlorotri(naphthalen-1-yl)phosphonium chloride: the product was isolated as white solid. **^{1}H NMR** (400 MHz, Acetonitrile-*d*3) δ [ppm] = 8.56 (d, *J* = 8.3 Hz, 4H), 8.25 (d, *J* = 8.7 Hz, 4H), 8.09 (br, 4H), 7.76-7.74 (m, 4H), 7.61 (br, 6H). **^{13}C NMR** (101 MHz, Acetonitrile-*d*3) δ [ppm] = 139.5 (d, *J* = 3.4 Hz), 134.8 (d, *J* = 10.6 Hz), 131.9 (d, *J* = 9.2 Hz), 130.8 (d, *J* = 2.2 Hz), 129.7, 128.3, 125.5 (d, *J* = 18.3 Hz). **^{31}P NMR** (162 MHz, Acetonitrile-*d*3) δ [ppm] = 62.28.

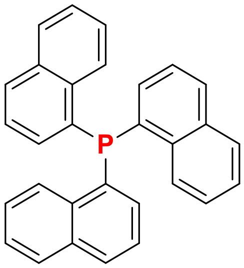

3g

Tri(naphthalen-1-yl)phosphane: the product was isolated as white solid. **^{1}H NMR** (400 MHz, Dichloromethane-*d*2) δ [ppm] = 8.50 (dd, *J* = 8.5, 4.1 Hz, 1H), 7.90 (d, *J* = 8.1 Hz, 1H), 7.85 (d, *J* = 8.2 Hz, 1H), 7.49 (t, *J* = 7.5 Hz, 1H), 7.41 (t, *J* = 7.6 Hz, 1H), 7.24 (t, *J* = 7.6 Hz, 1H), 6.93 (t, *J* = 5.8 Hz, 1H). **^{13}C NMR** (101 MHz, Dichloromethane-*d*2) δ [ppm] = 135.7 (d, *J* = 23.9 Hz), 133.7 (d, *J* = 4.9 Hz), 133.4, 132.8 (d, *J* = 11.0 Hz), 131.0, 129.7, 128.8, 126.4, 126.1, 125.8. **^{31}P NMR** (162 MHz, Dichloromethane-*d*2) δ [ppm] = −33.48. The chemical shift matches the literature values. [14]

Synthesis of (4-bromophenyl)diphenylphosphane 3h

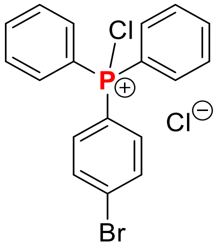

2h-Cl

(4-bromophenyl)chlorodiphenylphosphonium chloride: the product was isolated as white solid. ^{1}**H NMR** (400 MHz, Acetonitrile-d_3) δ [ppm] = 8.03 – 7.63 (m, 14H). ^{13}**C NMR** (101 MHz, Acetonitrile-d_3) δ [ppm] = 136.2, 134.8 (d, J = 14.4 Hz), 133.5 (d, J = 15.9 Hz), 133.2 (d, J = 13.5 Hz), 131.2, 130.3 (d, J = 15.9 Hz), 123.1 (d, J = 104.5 Hz), 122.8 (d, J = 106.0 Hz). ^{31}**P NMR** (162 MHz, Acetonitrile-d_3) δ [ppm] = 43.22.

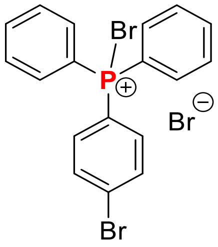

2h-Br

(4-bromophenyl)bromodiphenylphosphonium bromide: the product was isolated as pale yellow solid. ^{1}**H NMR** (400 MHz, Acetonitrile-d_3) δ [ppm] = 7.96 – 7.92 (m, 4H), 7.81 – 7.75 (m, 8H), 7.68 – 7.62 (m, 2H). ^{13}**C NMR** (101 MHz, Acetonitrile-d_3) δ [ppm] = 137.1 (d, J = 3.4 Hz), 135.7 (d, J = 14.0 Hz), 134.2 (d, J = 13.0 Hz), 133.9 (d, J = 15.4 Hz), 132.4 (d, J = 3.9 Hz), 130.7 (d, J = 14.4 Hz), 119.0 (d, J = 87.2 Hz), 118.8 (d, J = 89.6 Hz). ^{31}**P NMR** (162 MHz, Acetonitrile-d_3) δ [ppm] = 50.29.

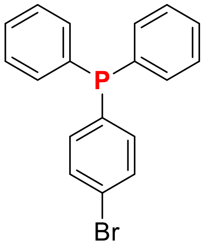

3h

(4-bromophenyl)diphenylphosphane: the product was isolated as white solid. ^{1}**H NMR** (400 MHz, Acetonitrile-d_3) δ [ppm] = 7.50 (d, J = 7.3 Hz, 2H), 7.39 – 7.33 (m, 6H), 7.30 – 7.22 (m, 4H), 7.15 (dd, J = 8.6, 6.9 Hz, 2H). ^{13}**C NMR** (101 MHz, Acetonitrile-d_3) δ [ppm] = 137.0 (d, J = 12.8 Hz), 136.7 (d, J = 11.3 Hz), 135.2 (d, J = 20.2 Hz), 133.6 (d, J = 20.0 Hz), 131.7 (d, J = 7.1 Hz), 129.3, 128.9 (d, J = 6.8 Hz), 123.0. ^{31}**P NMR** (162 MHz, Acetonitrile-d_3) δ [ppm] = –6.32. The chemical shift matches the literature values.[15]

Synthesis of (4-bromophenyl)diphenylphosphane 3i

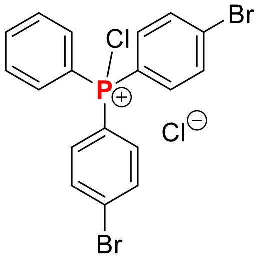

2i-Cl

Bis(4-bromophenyl)chloro(phenyl)phosphonium chloride: the product was isolated as white solid. **^{1}H NMR** (400 MHz, Acetonitrile-d_3) δ [ppm] = 7.90 – 7.67 (m, 13H). **^{13}C NMR** (101 MHz, Acetonitrile-d_3) δ [ppm] = 136.0 (d, *J* = 3.9 Hz), 134.6 (d, *J* = 14.4 Hz), 133.4 (d, *J* = 16.9 Hz), 133.0 (d, *J* = 13.5 Hz), 131.0 (d, *J* = 4.2 Hz), 130.2 (d, *J* = 16.4 Hz), 123.8 (d, *J* = 106.0 Hz), 123.4 (d, *J* = 110.3 Hz). **^{31}P NMR** (162 MHz, Acetonitrile-d_3) δ [ppm] = 27.06.

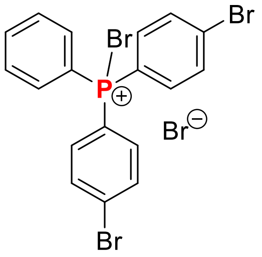

2i-Br

Bis(4-bromophenyl)bromo(phenyl)phosphonium bromide: the product was isolated as pale yellow solid. **^{1}H NMR** (400 MHz, Acetonitrile-d_3) δ [ppm] = 7.97 – 7.81 (m, 5H), 7.82 – 7.75 (m, 4H), 7.69 – 7.63 (m, 4H). **^{13}C NMR** (101 MHz, Acetonitrile-d_3) δ [ppm] = 137.3 (d, *J* = 3.9 Hz), 135.7 (d, *J* = 14.4 Hz), 134.3 (d, *J* = 13.5 Hz), 134.0 (d, *J* = 15.4 Hz), 132.6 (d, *J* = 4.3 Hz), 130.8 (d, *J* = 14.9 Hz), 118.6 (d, *J* = 87.7 Hz), 118.3 (d, *J* = 90.1 Hz). **^{31}P NMR** (162 MHz, Acetonitrile-d_3) δ [ppm] = 49.96.

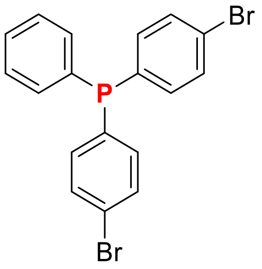

3i

Bis(4-bromophenyl)(phenyl)phosphane: the product was isolated as white solid. **^{1}H NMR** (400 MHz, Acetonitrile-d_3) δ [ppm] = 7.51 (d, *J* = 6.7 Hz, 4H), 7.37 (m, 3H), 7.27 (m, 2H), 7.15 (t, *J* = 7.6 Hz 4H). **^{13}C NMR** (101 MHz, Acetonitrile-d_3) δ [ppm] = 136.3 (d, *J* = 12.6 Hz), 136.0 (d, *J* = 10.4 Hz), 135.3 (d, *J* = 20.4 Hz), 133.6 (d, *J* = 20.5 Hz), 131.9 (d, *J* = 7.0 Hz), 129.5, 129.0 (d, *J* = 7.3 Hz), 123.3. **^{31}P NMR** (162 MHz, Acetonitrile-d_3) δ [ppm] = −7.33. The chemical shift matches the literature values.[16]

Synthesis of 5-phenyl-5H-benzo[b]phosphindole 3j

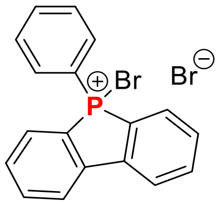

3j-Br

5-bromo-5-phenyl-5*H*-benzo[*b*]phosphindol-5-ium bromide: the product was isolated as yellow solid. **^{1}H NMR** (400 MHz, Acetonitrile-d_3) δ [ppm] = 8.17 (dd, *J* = 7.9, 4.9 Hz, 2H), 8.14 – 8.11 (d, *J* = 7.7 Hz, 2H), 8.04 – 7.86 (m, 5H), 7.79 – 7.65 (m, 4H). **^{13}C NMR** (101 MHz, Acetonitrile-d_3) δ [ppm] = 143.2 (d, *J* = 24.5 Hz), 138.4 (d, *J* = 2.6 Hz), 137.1 (d, *J* = 3.6 Hz), 132.8 (d, *J* = 14.4 Hz), 132.4 (d, *J* = 11.7 Hz), 132.0 (d, *J* = 13.2 Hz), 130.8 (d, *J* = 15.7 Hz), 124.2 (d, *J* = 11.2 Hz), 121.8 (d, *J* = 93.4 Hz), 118.2 (d, *J* = 85.4 Hz). **^{31}P NMR** (162 MHz, Acetonitrile-d_3) δ [ppm] = 37.83. The chemical shift matches the literature values.[4]

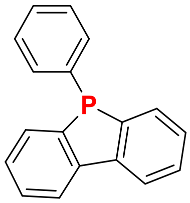

3j

5-phenyl-5*H*-benzo[*b*]phosphindole: the product was isolated as white solid. **^{1}H NMR** (400 MHz, Acetonitrile-d_3) δ [ppm] = 8.03 (d, *J* = 7.9 Hz, 2H), 7.72 (dd, *J* = 7.0, 5.4 Hz, 2H), 7.49 (td, *J* = 7.6, 1.2 Hz, 2H), 7.41 – 7.31 (m, 2H), 7.28 – 7.21 (m, 5H). **^{13}C NMR** (101 MHz, Acetonitrile-d_3) δ [ppm] = 143.6 (d, *J* = 2.6 Hz), 142.3 (d, *J* = 3.1 Hz), 132.1 (d, *J* = 19.3 Hz), 130.4 (d, *J* = 22.2 Hz), 129.3 (d, *J* = 42.4 Hz), 129.0 (s), 128.9 (d, *J* = 7.7 Hz), 127.9 (d, *J* = 7.2 Hz), 121.8. **^{31}P NMR** (162 MHz, Acetonitrile-d_3) δ [ppm] = –10.02. The chemical shift matches the literature values.[17]

Synthesis of *tert*-butyldiphenylphosphane 3k

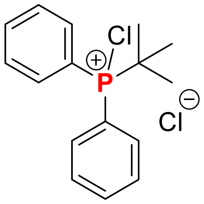

2k-Cl

Tert-butylchlorodiphenylphosphonium chloride: the product was isolated as white solid. **^{1}H NMR** (400 MHz, Acetonitrile-d_3) δ [ppm] = 8.01 – 7.92 (m, 6H), 7.79 – 7.74 (m, 4H), 1.54 (d, J = 21.0 Hz, 9H). **^{13}C NMR** (101 MHz, Acetonitrile-d_3) δ [ppm] = 136.7 (d, J = 3.4 Hz), 134.0 (d, J = 11.1 Hz), 130.5 (d, J = 13.5 Hz), 118.1, 39.1 (d, J = 36.6 Hz), 24.3. **^{31}P NMR** (162 MHz, Acetonitrile-d_3) δ [ppm] = 86.84. The chemical shift matches the literature values.[13]

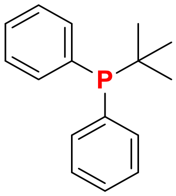

3k

Tert-butyldiphenylphosphane: the product was isolated as colorless oil. **^{1}H NMR** (400 MHz, Acetonitrile-d_3) δ [ppm] = 7.56 – 7.50 (m, 4H), 7.34 (m, 6H), 1.11 (d, J = 12.5 Hz, 9H). **^{13}C NMR** (101 MHz, Acetonitrile-d_3) δ [ppm] = 137.1 (d, J = 19.3 Hz), 134.6 (d, J = 19.7 Hz), 128.7, 128.2 (d, J = 7.2 Hz), 30.2 (d, J = 14.4 Hz), 28.0 (d, J = 14.9 Hz). **^{31}P NMR** (162 MHz, Acetonitrile-d_3) δ [ppm] = 17.84. The chemical shift matches the literature values.[18]

Synthesis of cyclohexyldiphenylphosphane 3l

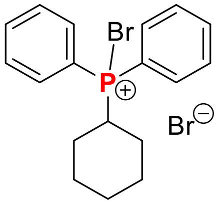

2l-Br

Bromo(cyclohexyl)diphenylphosphonium bromide: the product was isolated as amorphous white solid. ^{1}**H NMR** (400 MHz, Acetonitrile-*d*$_3$) δ [ppm] = 7.94 – 7.82 (m, 6H), 7.80 – 7.70 (m, 6H), 3.59 (tdt, *J* = 11.9, 4.1, 3.0 Hz, 1H), 2.05 – 1.11 (m, 10H). ^{13}**C NMR** (101 MHz, Acetonitrile-*d*$_3$) δ [ppm] = 136.5 (d, *J* = 3.4 Hz), 133.5 (d, *J* = 11.6 Hz), 130.6 (d, *J* = 13.5 Hz), 118.2 (overlap with solvent), 37.3 (d, *J* = 36.6 Hz), 26.0 (d, *J* = 3.9 Hz), 25.2 (d, *J* = 16.4 Hz), 24.6 (d, *J* = 2.4 Hz).^{31}**P NMR** (162 MHz, Acetonitrile-*d*$_3$) δ [ppm] = 68.70.

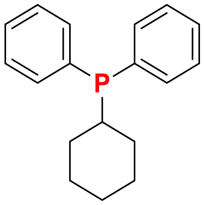

3l

Cyclohexyldiphenylphosphane: the product was isolated as colorless oil. ^{1}**H NMR** (400 MHz, Acetonitrile-*d*$_3$) δ [ppm] = 7.49 – 7.45 (m, 4H), 7.34 – 7.30 (m, 6H), 2.34 – 2.25 (m, 1H), 1.71 – 1.57 (m, 5H), 1.39 – 1.09 (m, 5H). ^{13}**C NMR** (101 MHz, Acetonitrile-*d*$_3$) δ [ppm] = 137.6 (d, *J* = 14.5 Hz), 133.5 (d, *J* = 19.3 Hz), 128.8, 128.5 (d, *J* = 6.7 Hz), 34.7 (d, *J* = 8.2 Hz), 29.5 (d, *J* = 15.9 Hz), 26.4 (d, *J* = 11.6 Hz), 26.2. ^{31}**P NMR** (162 MHz, Acetonitrile-*d*$_3$) δ [ppm] = –4.26. The chemical shift matches the literature values.[11]

Synthesis of 2m-Cl

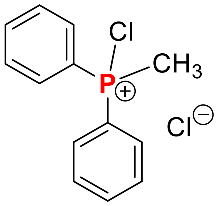

2m-Cl

Chloro(methyl)diphenylphosphonium chloride: the product was isolated as white solid. **^{1}H NMR** (400 MHz, Dichloromethane-d_2) δ [ppm] = 8.25 – 8.13 (m, 4H), 7.87 – 7.73 (m, 2H), 7.72 – 7.59 (m, 4H), 3.75 (d, J = 13.4 Hz, 3H). **^{13}C NMR** (400 MHz, Dichloromethane-d_2) δ [ppm] = 135.9 (d, J = 3.4 Hz), 132.3 (d, J = 13.5 Hz), 130.1 (d, J = 15.4 Hz), 122.8 (d, J = 95.4 Hz), 19.2 (d, J = 55.9 Hz). **^{31}P NMR** (400 MHz, Dichloromethane-d_2) δ [ppm] = 61.61. The chemical shift matches the literature values.[1]

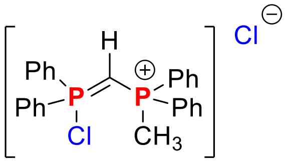

^{31}P NMR (400 MHz, Dichloromethane-d_2) δ [ppm] = 60.34 (d, J = 8.7 Hz), 17.89 (d, J = 8.7 Hz). The chemical shift matches the literature values.[19]

Synthesis of 2n-Cl

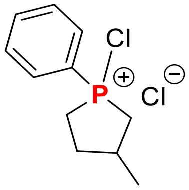

1-chloro-3-methyl-1-phenylphospholan-1-ium chloride: **^{31}P NMR** (162 MHz, Acetonitrile-d_3) δ [ppm] = 80.6.

Synthesis of 2o-Br

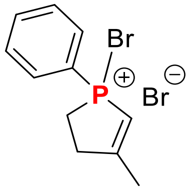

2o-Br

1-bromo-4-methyl-1-phenyl-2,3-dihydro-1H-phosphol-1-ium bromide: the product was isolated as pale yellow solid. **^{1}H NMR** (400 MHz, Acetonitrile-d_3) δ [ppm] = 8.01 (dd, *J* = 16.3, 7.5 Hz, 2H), 7.87 – 7.78 (m, 2H), 7.76 – 7.64 (m, 2H), 6.57 (d, *J* = 32.8 Hz, 1H), 3.44 – 3.36 (m, 2H), 3.32 – 3.21 (m, 2H), 2.28 (s, 3H). **^{13}C NMR** (101 MHz, Acetonitrile-d_3) δ [ppm] = 182.2 (d, *J* = 29.1 Hz), 136.0 (d, *J* = 3.4 Hz), 132.2 (d, *J* = 13.7 Hz), 130.1 (d, *J* = 15.0 Hz), 122.9 (d, *J* = 84.1 Hz), 109.2 (d, *J* = 80.4 Hz), 37.1 (d, *J* = 8.0 Hz), 29.6 (d, *J* = 48.8 Hz), 21.3 (d, *J* = 19.5 Hz). **^{31}P NMR** (162 MHz, Acetonitrile-d_3) δ [ppm] = 79.08. The chemical shift matches the literature values.[4]

6. NMR spectra

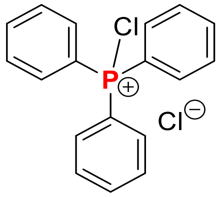

2a-Cl

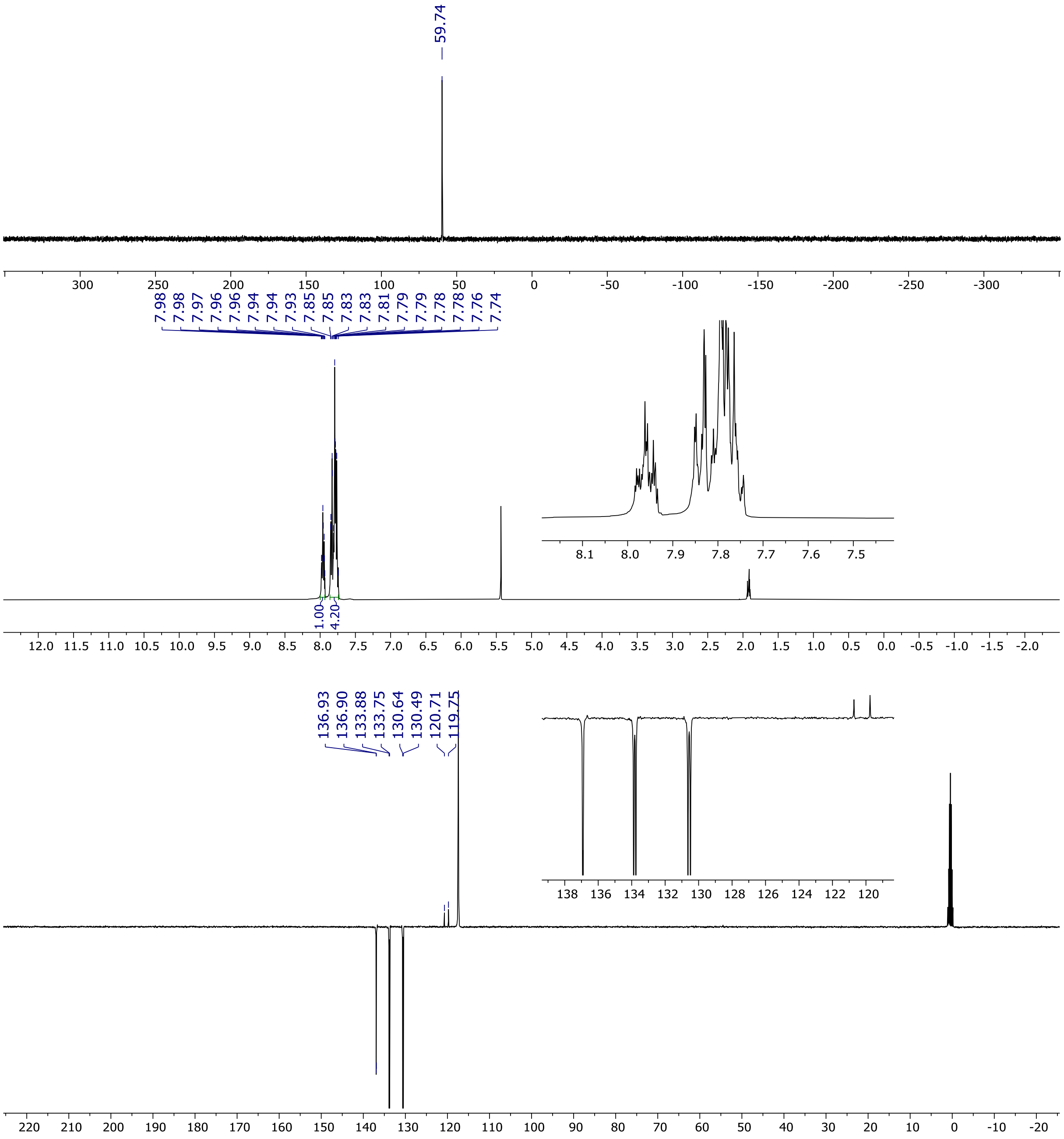

Fig. S2. *^{31}P (162 MHz), ^{1}H (400 MHz) and ^{13}C NMR (101 MHz) of* ***2a-Cl*** *in ACN-d_3*

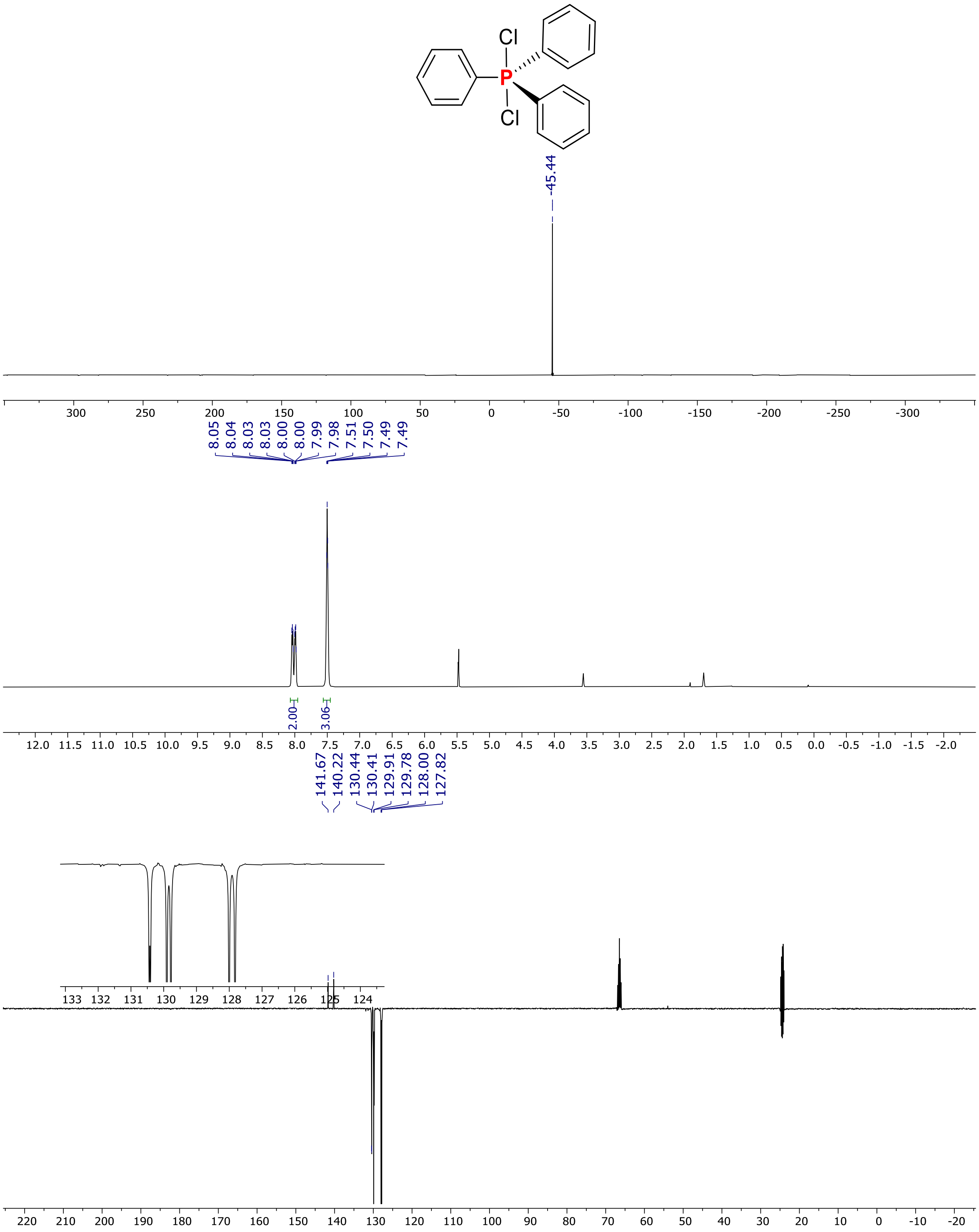

Fig. S3. *^{31}P (162 MHz), ^{1}H (400 MHz) and ^{13}C NMR (101 MHz) NMR of* ***2a-Cl*** *in THF-d_8*

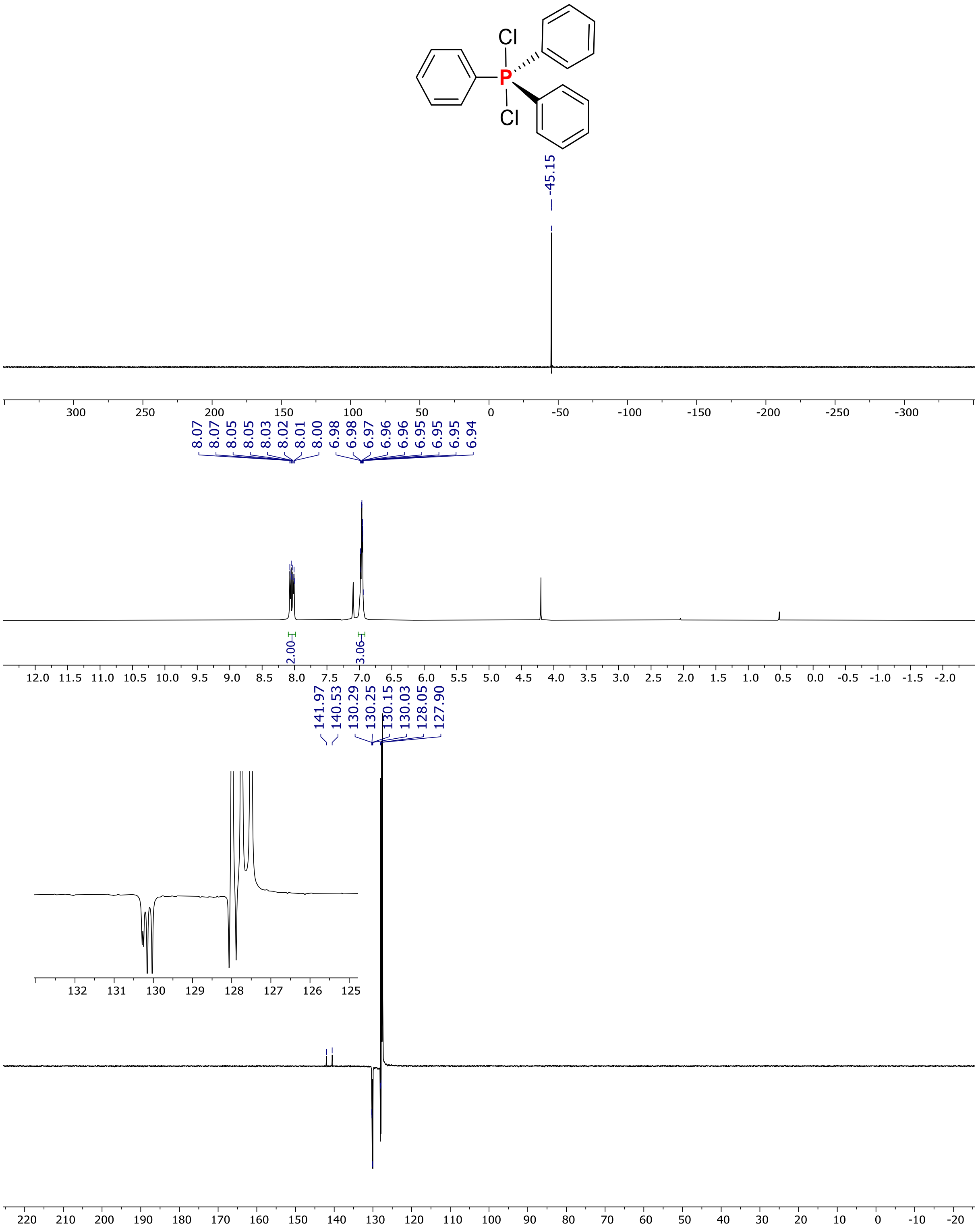

Fig. S4. *^{31}P (162 MHz), ^{1}H (400 MHz) and ^{13}C NMR (101 MHz) NMR of* ***2a-Cl*** *in Benzene-d_6*

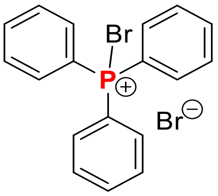

2a-Br

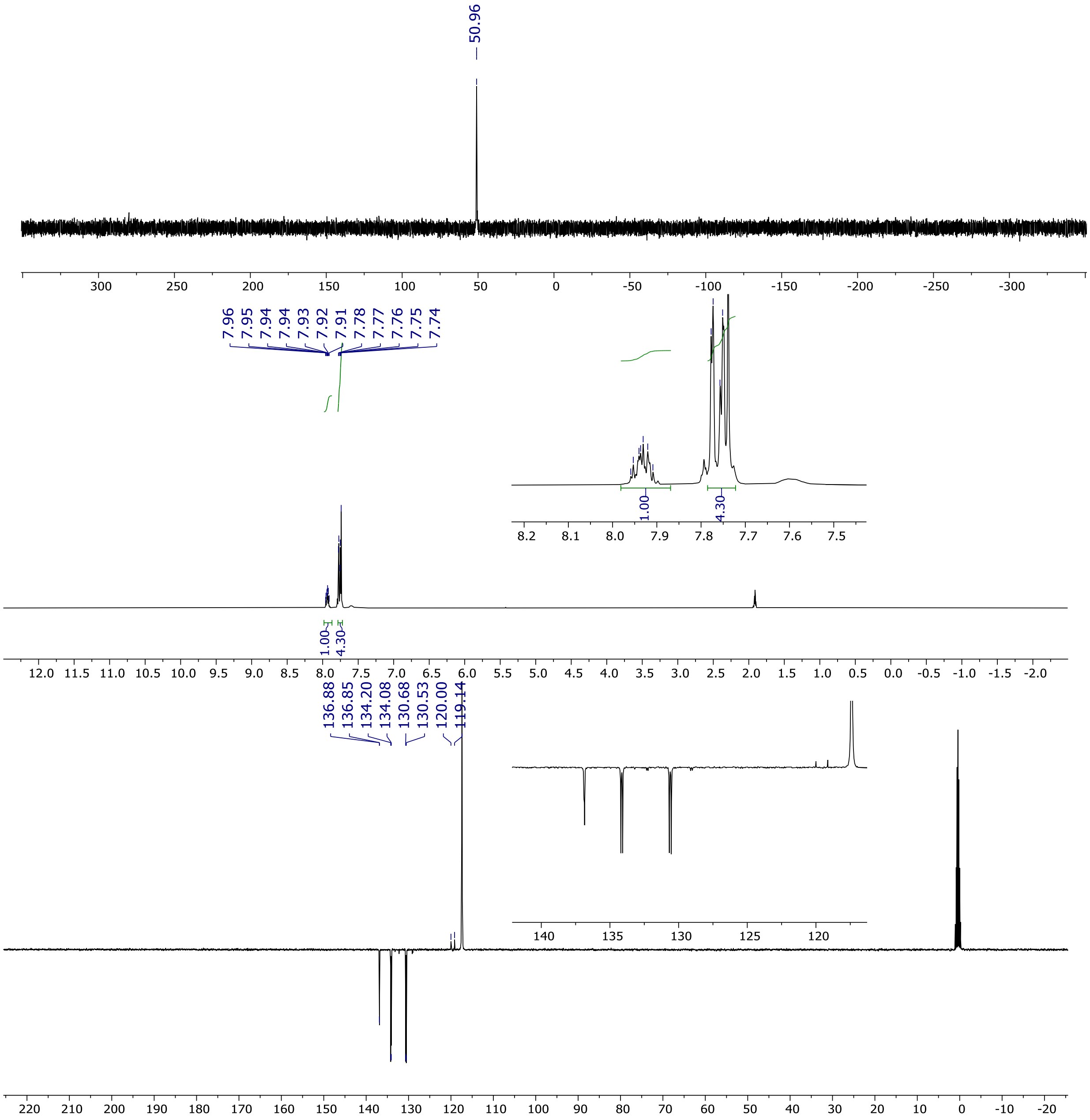

Fig. S5. *^{31}P (162 MHz), ^{1}H (400 MHz) and ^{13}C NMR (101 MHz) NMR of* ***2a-Br*** *in ACN-d_3*

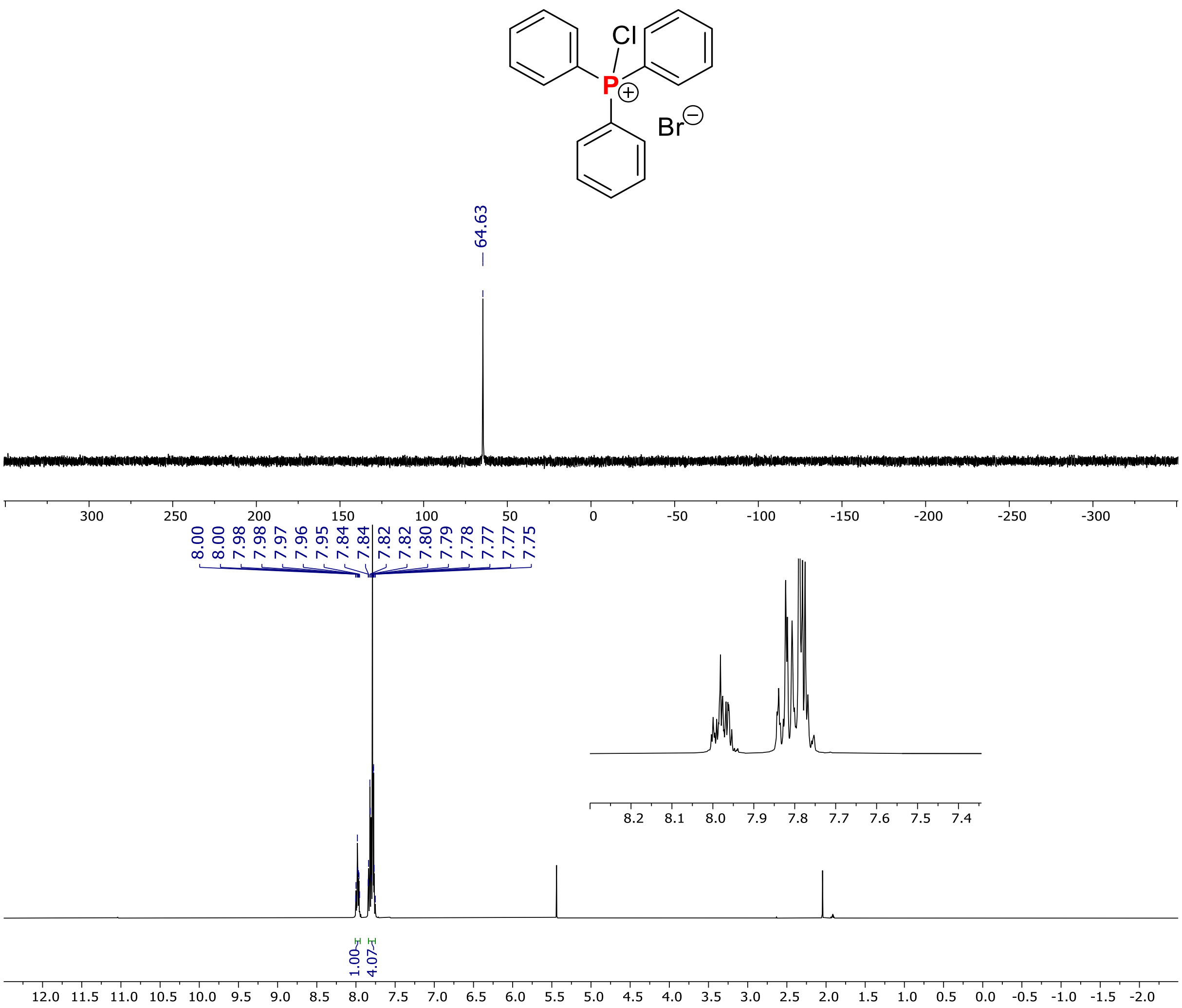

Fig. S6. *^{31}P (162 MHz)and ^{1}H (400 MHz) NMR of $[Ph_3PCl]^+Br^-$ in ACN-d_3*

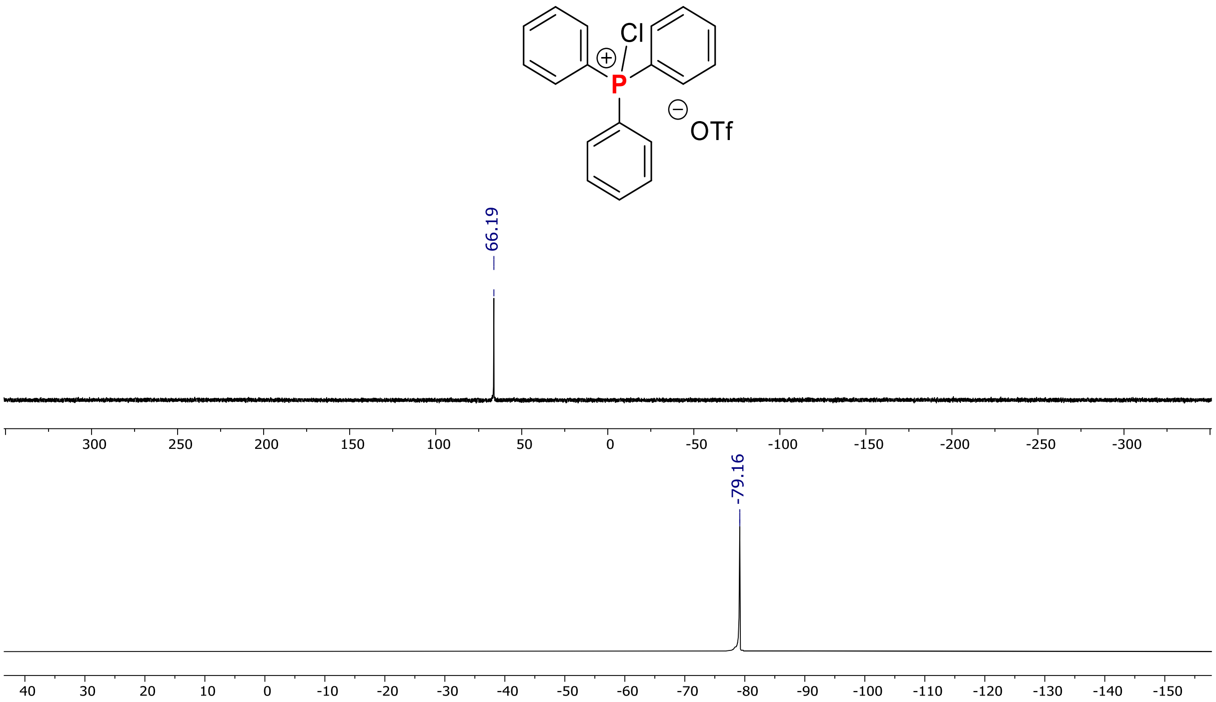

Fig. S7. *^{31}P (162 MHz) and ^{19}F (376 MHz) NMR of $[Ph_3PCl]^+OTf^-$ in ACN-d_3*

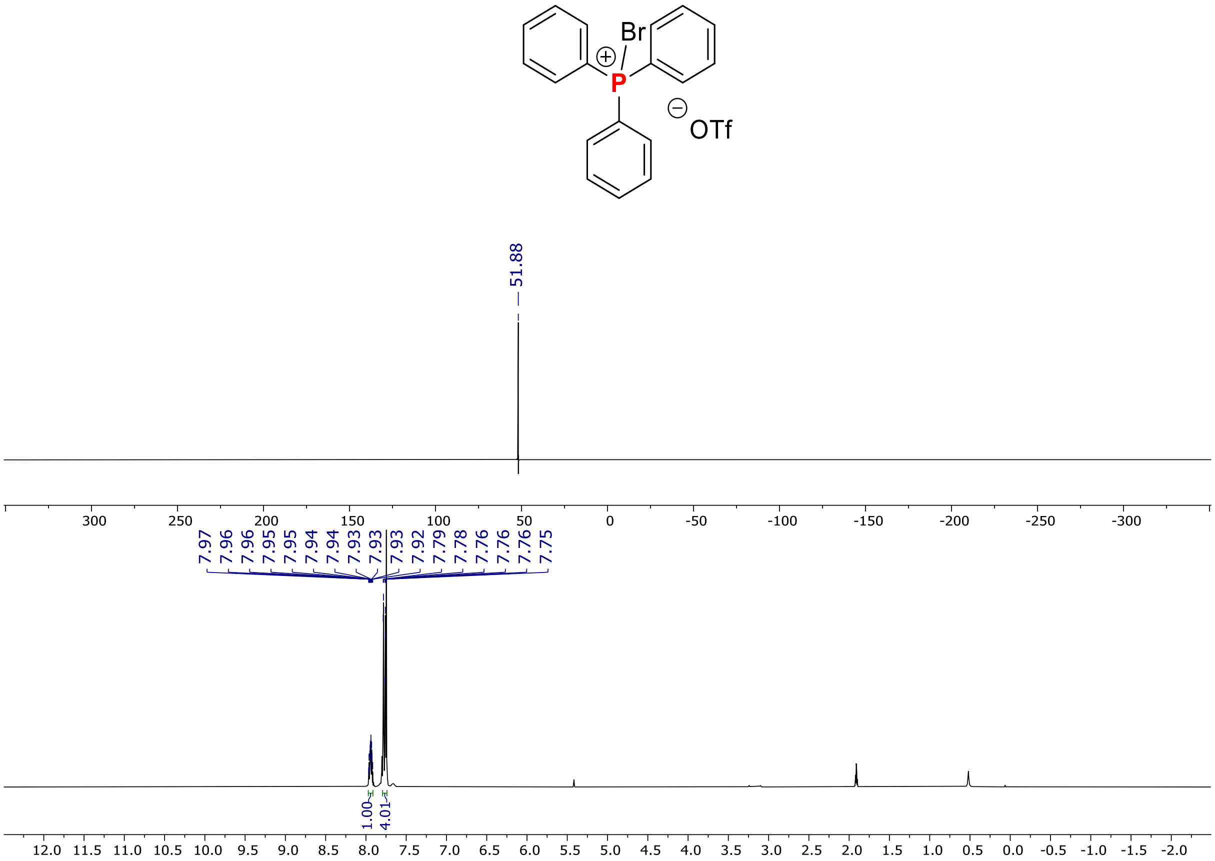

Fig. S8. ^{31}P (162 MHz) and ^{1}H (400 MHz) NMR of $[Ph_3PBr]^{+}OTf^{-}$ in ACN-d_3

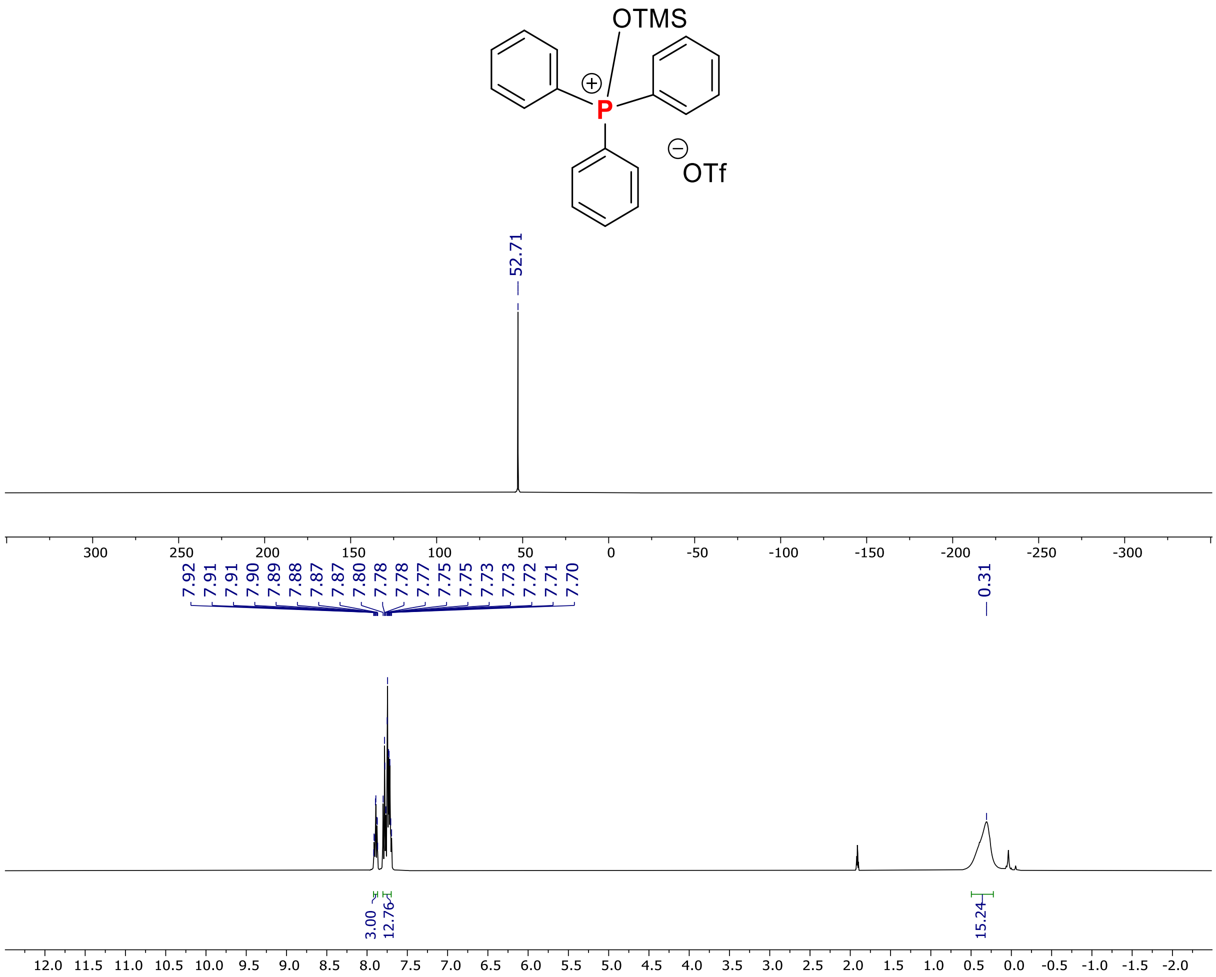

Fig. S9. *^{31}P (162 MHz) and ^{1}H (400 MHz) NMR of $[Ph_3POTMS]^+OTf^-$ in ACN-d_3*

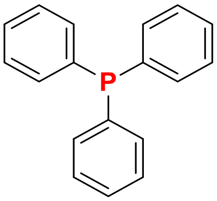

3a

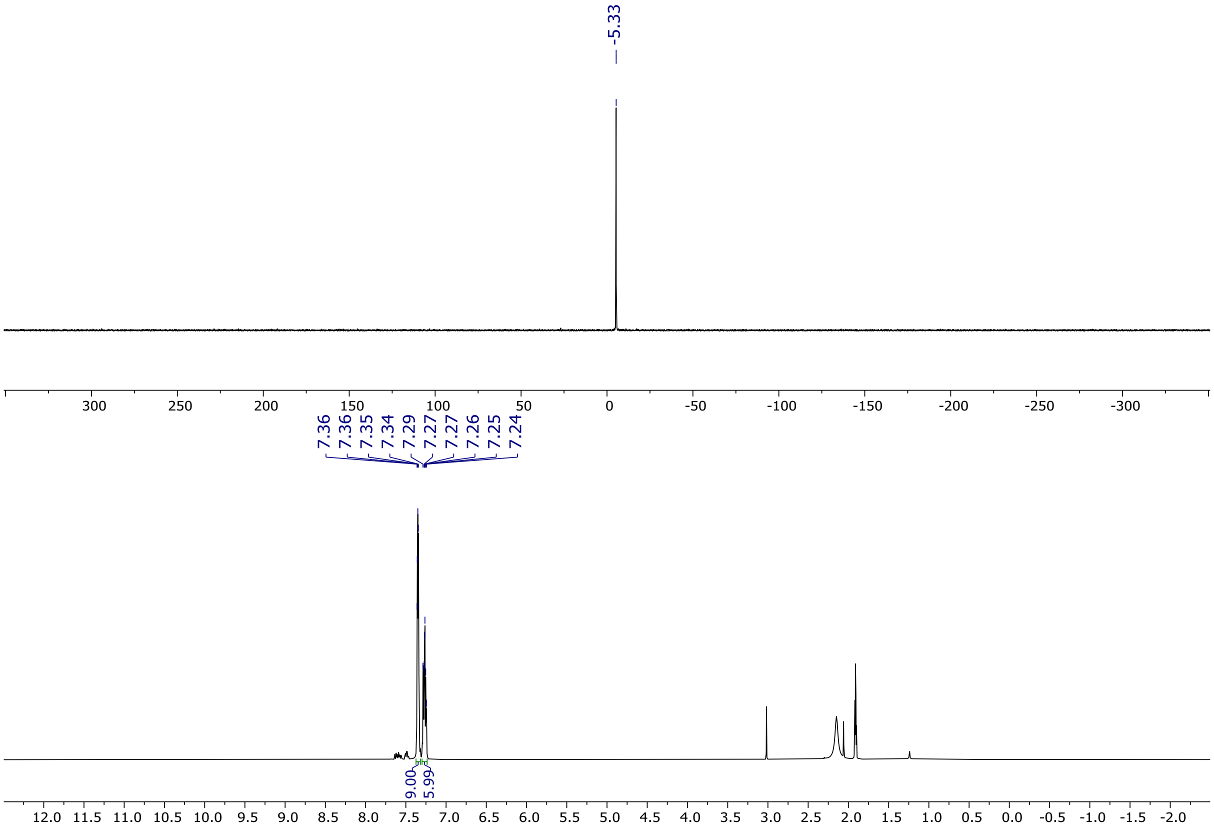

Fig. S10. *^{31}P (162 Mhz) and ^{1}H (400 MHz) NMR of* ***3a*** *in ACN-d_3*

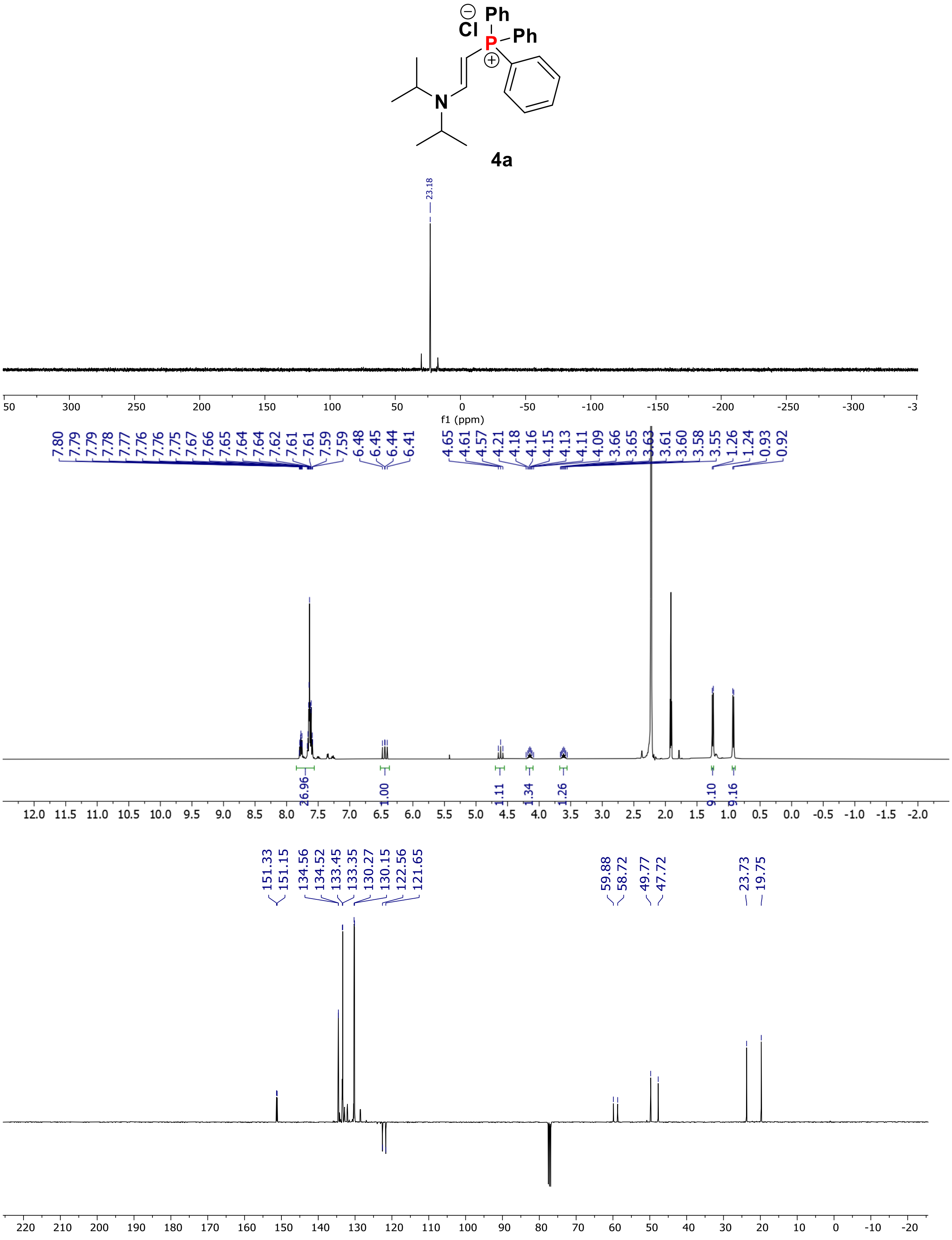

Fig. S11. *^{31}P (162 MHz), ^{1}H (400 MHz) and ^{13}C NMR (101 MHz) NMR of **4a** in CD_3Cl*

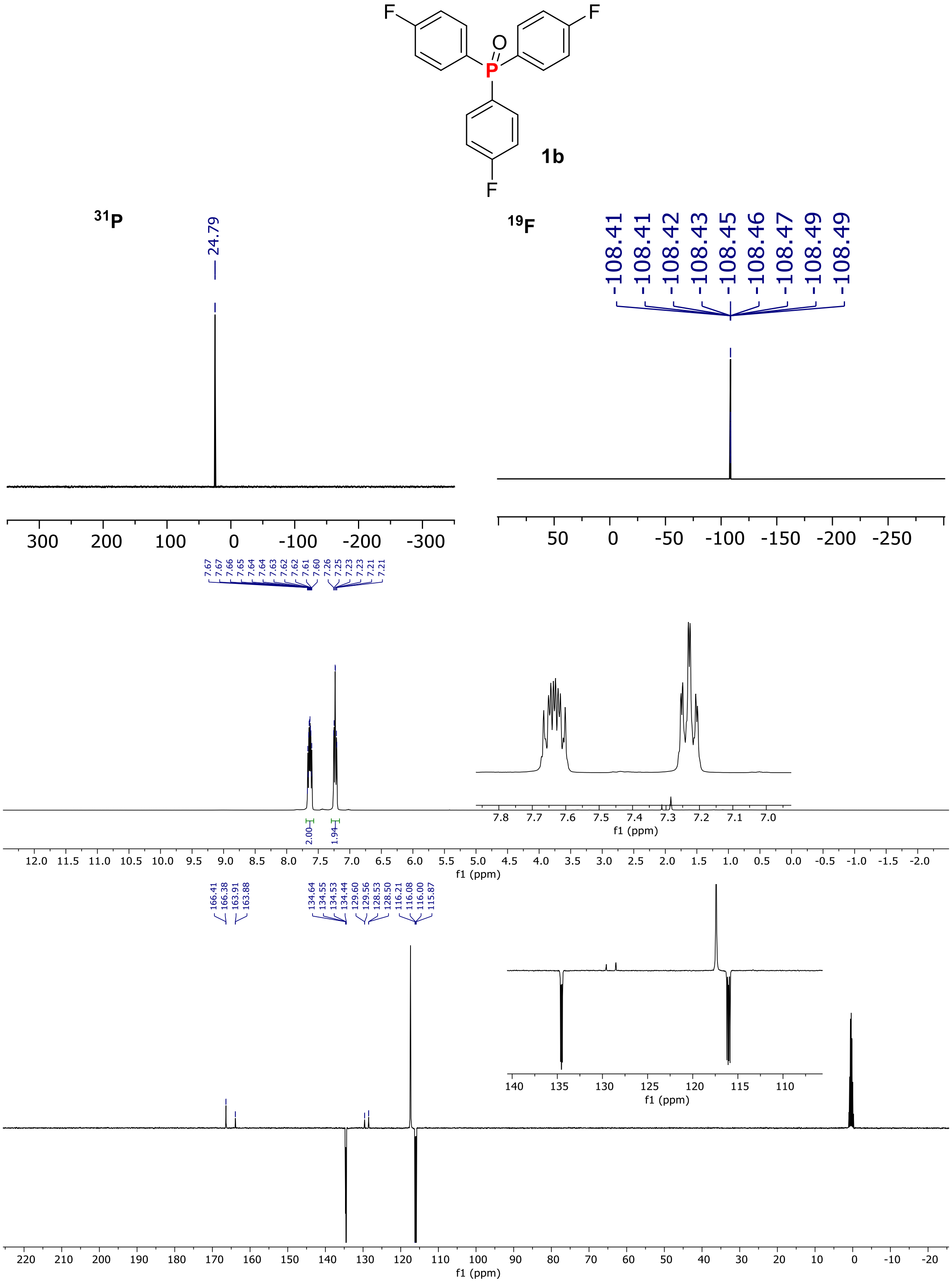

Fig. S12. *^{31}P (162 MHz), ^{19}F (376 MHz), ^{1}H (400 MHz) and ^{13}C (101 MHz) NMR of **1b** in ACN-d_3*

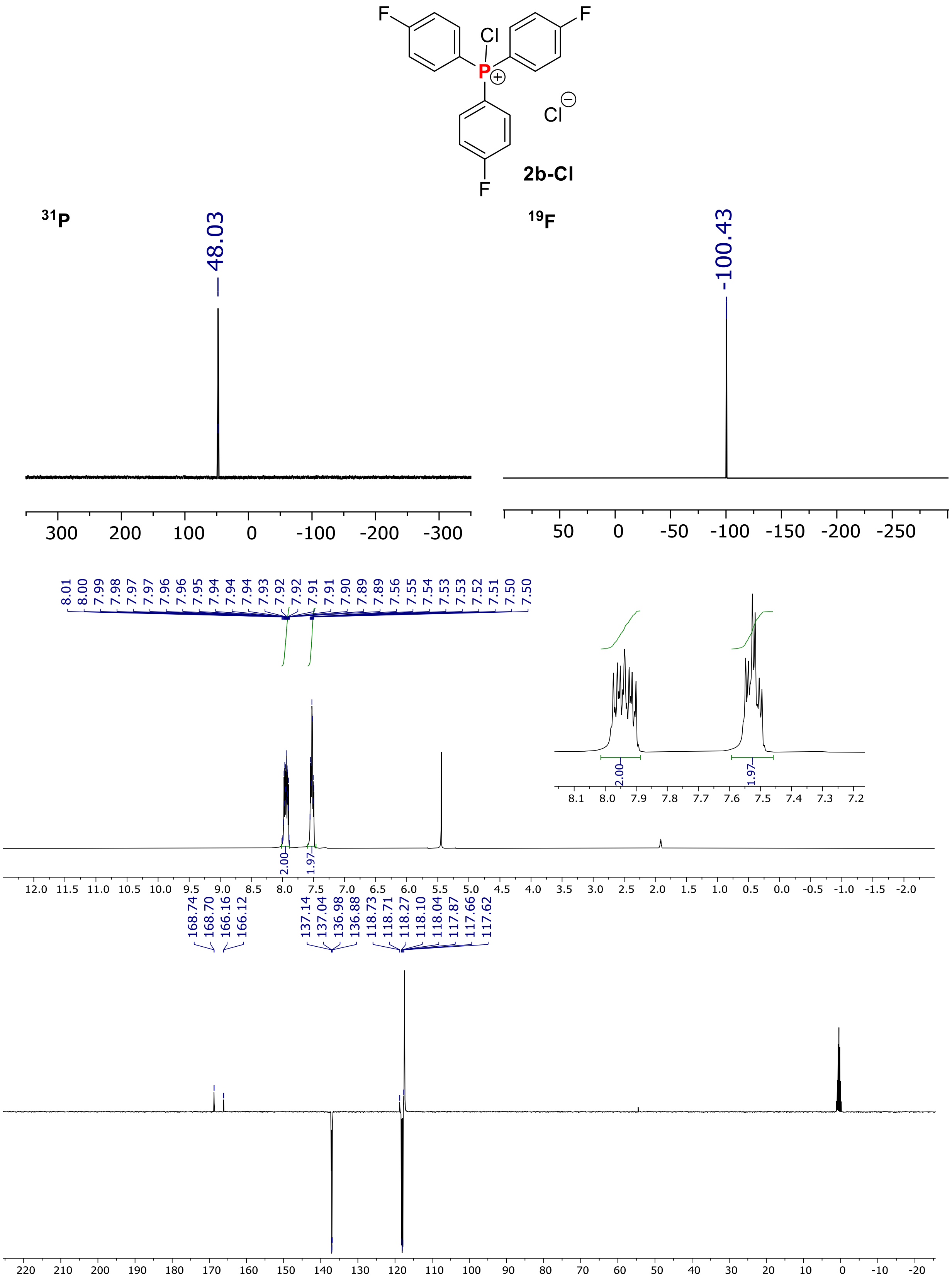

Fig. S13. ^{31}P (162 MHz), ^{19}F (376 MHz), ^{1}H (400 MHz) and ^{13}C (101 MHz) NMR of **2b-Cl** in ACN-d_3

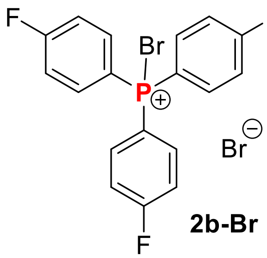

2b-Br

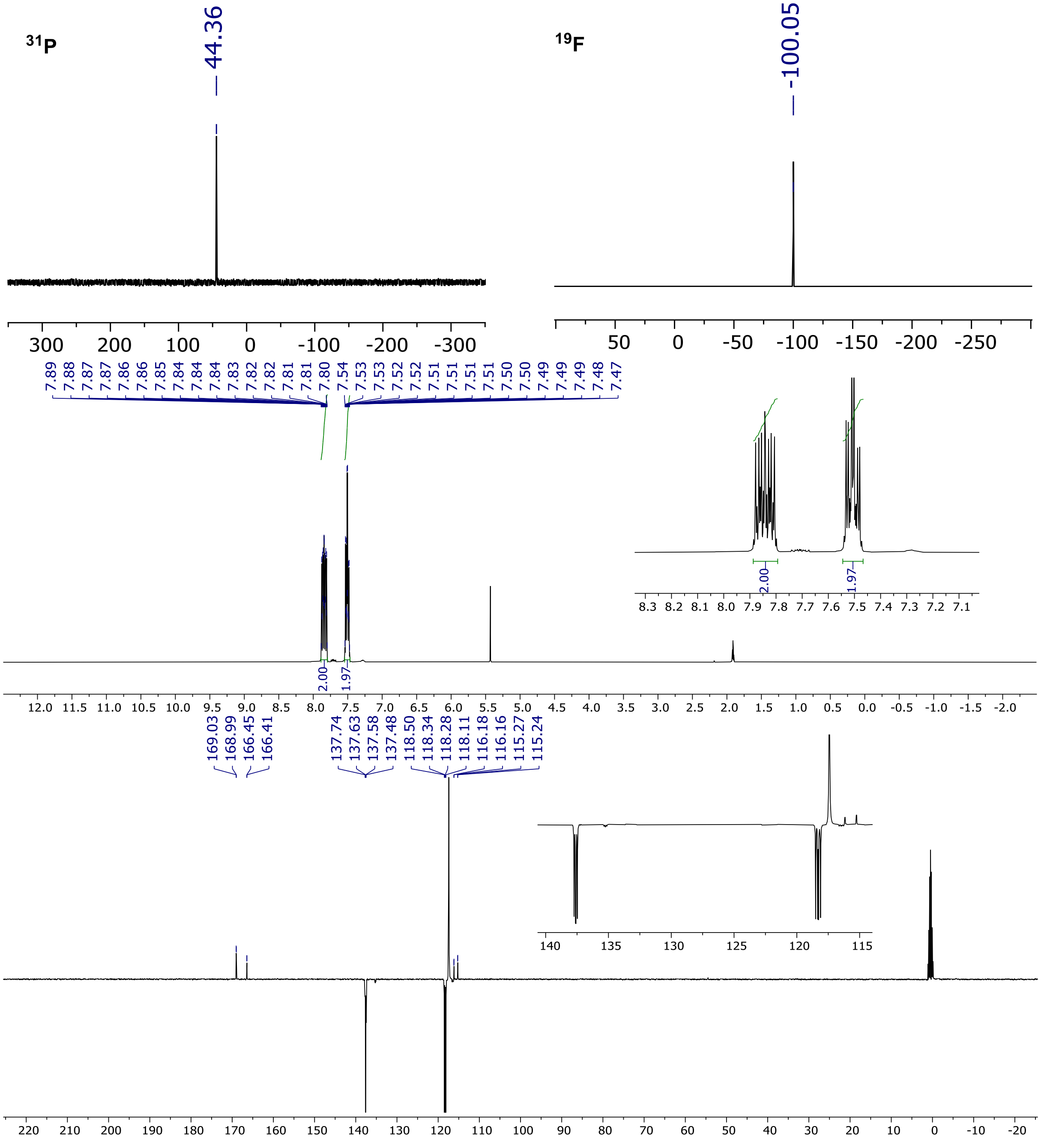

Fig. S14. *^{31}P (162 MHz), ^{19}F (376 MHz), ^{1}H (400 MHz) and ^{13}C (101 MHz) NMR of* ***2b-Br*** *in ACN-d_3*

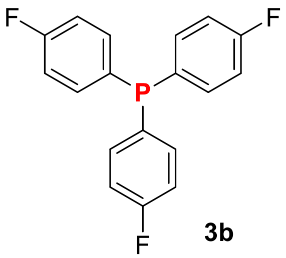

3b

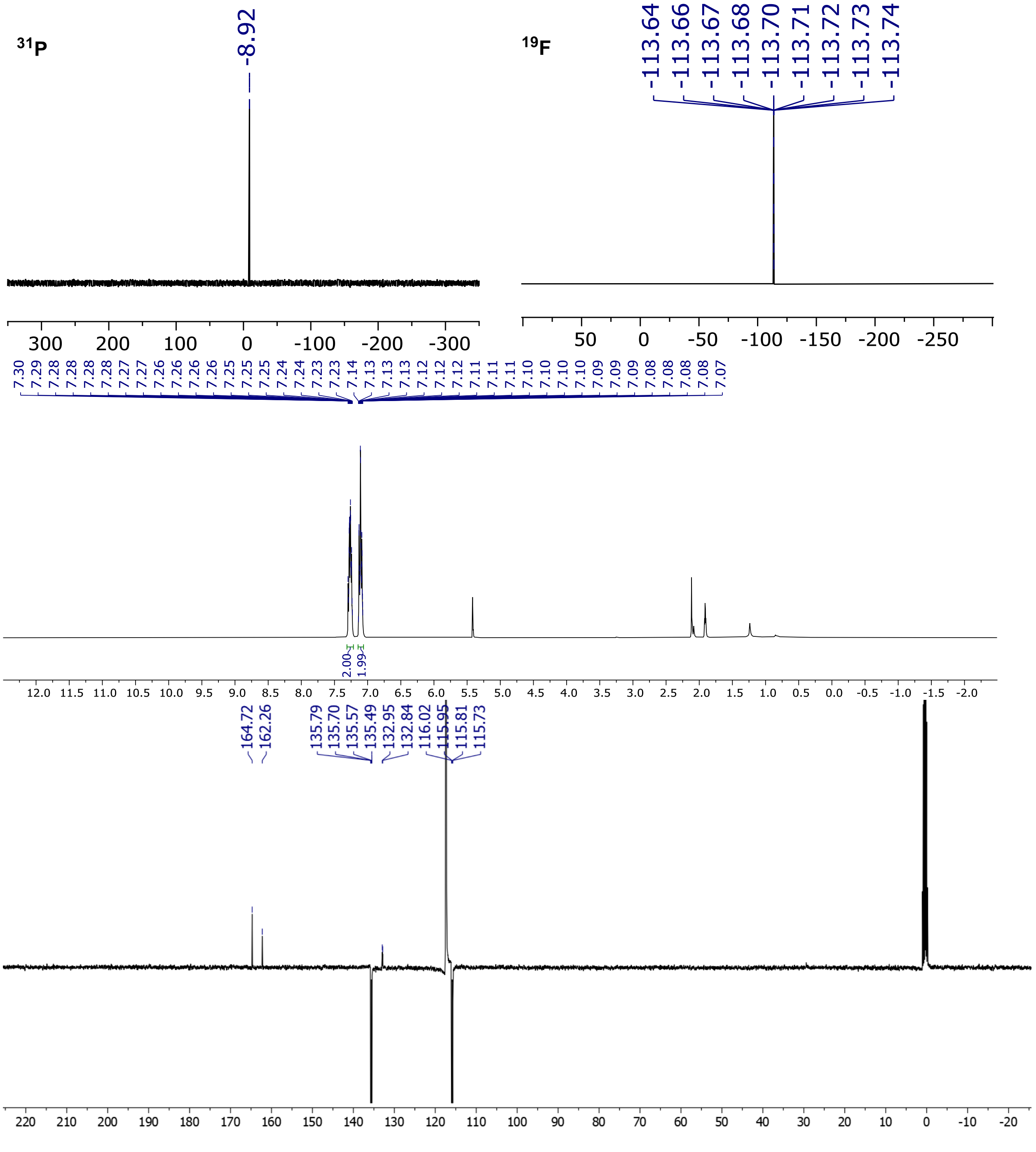

Fig. S15. *^{31}P (162 MHz), ^{19}F (376 MHz), ^{1}H (400 MHz) and ^{13}C (101 MHz) NMR of* ***3b*** *in ACN-d_3*

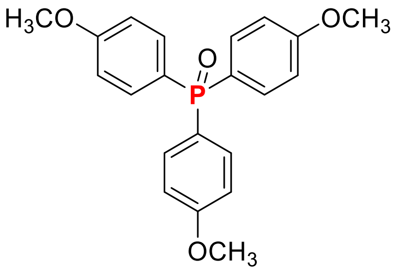
H3CO
OCH3
O
P
OCH3

1c

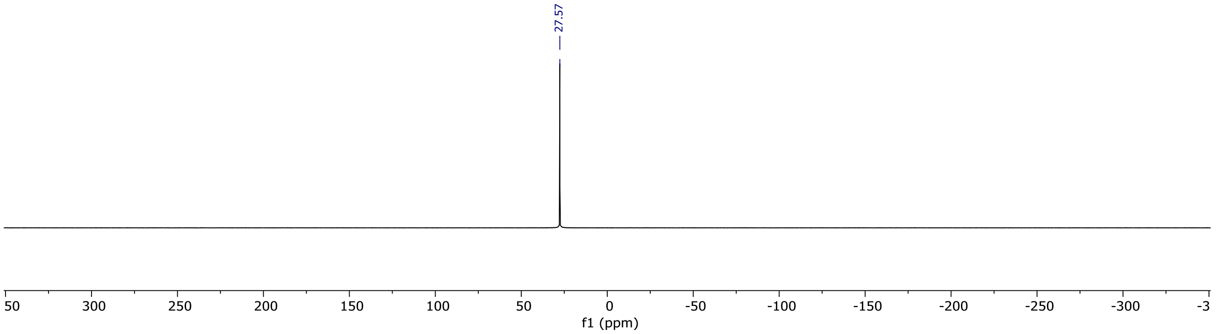
27.57
f1 (ppm)

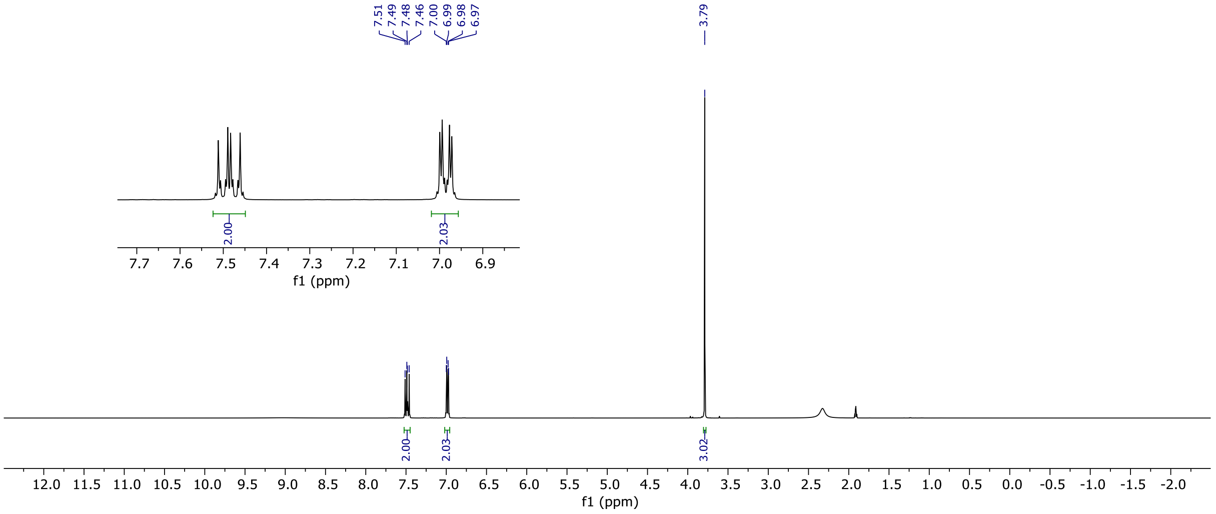
7.51
7.49
7.48
7.46
7.00
6.99
6.98
6.97
3.79
2.00
2.03
3.02
f1 (ppm)

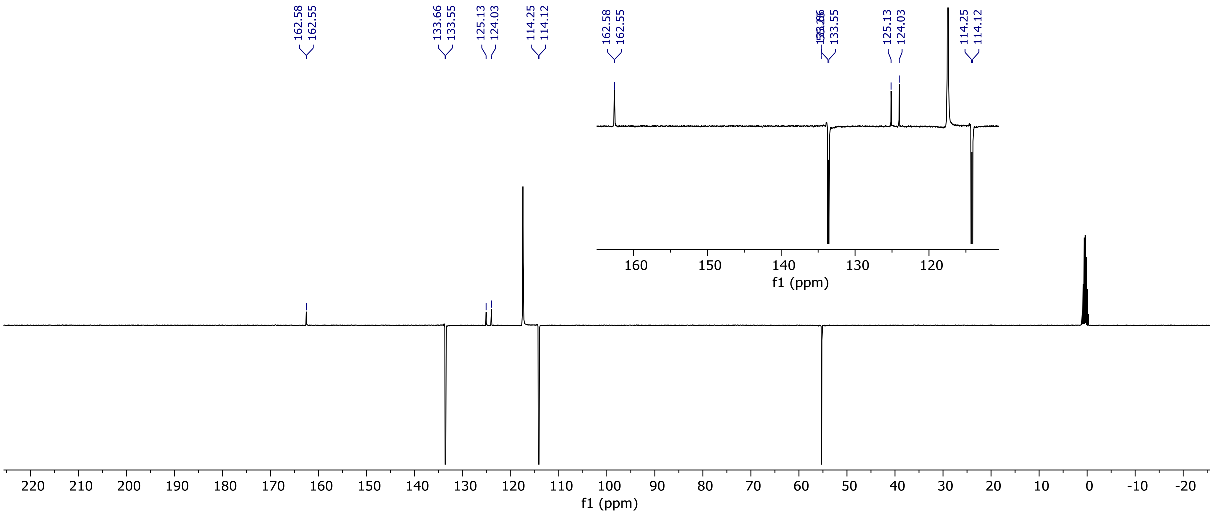

Fig. S16. *^{31}P (162 MHz), ^{1}H (400 MHz) and ^{13}C (101 MHz) NMR of **1c** in ACN-d$_3$*

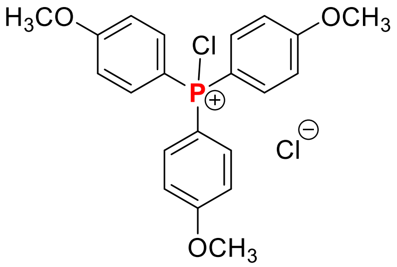

2c-Cl

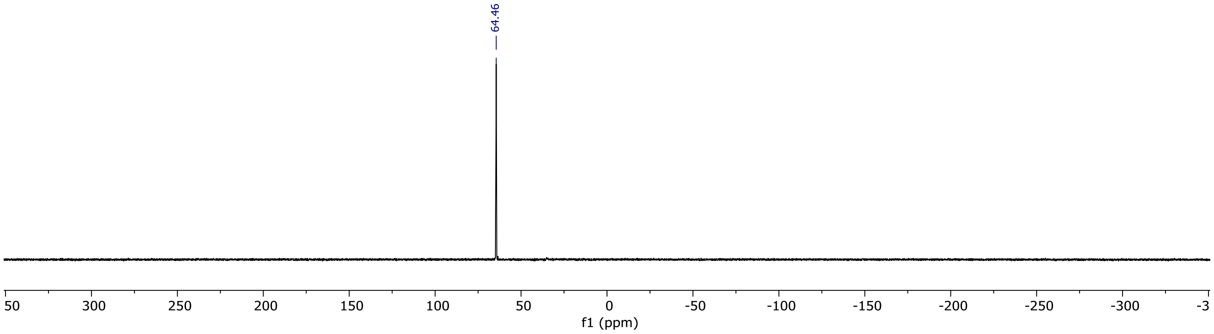

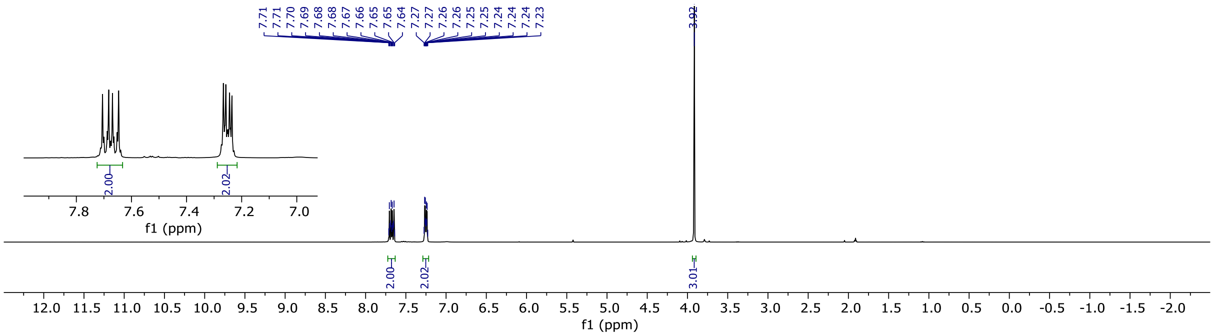

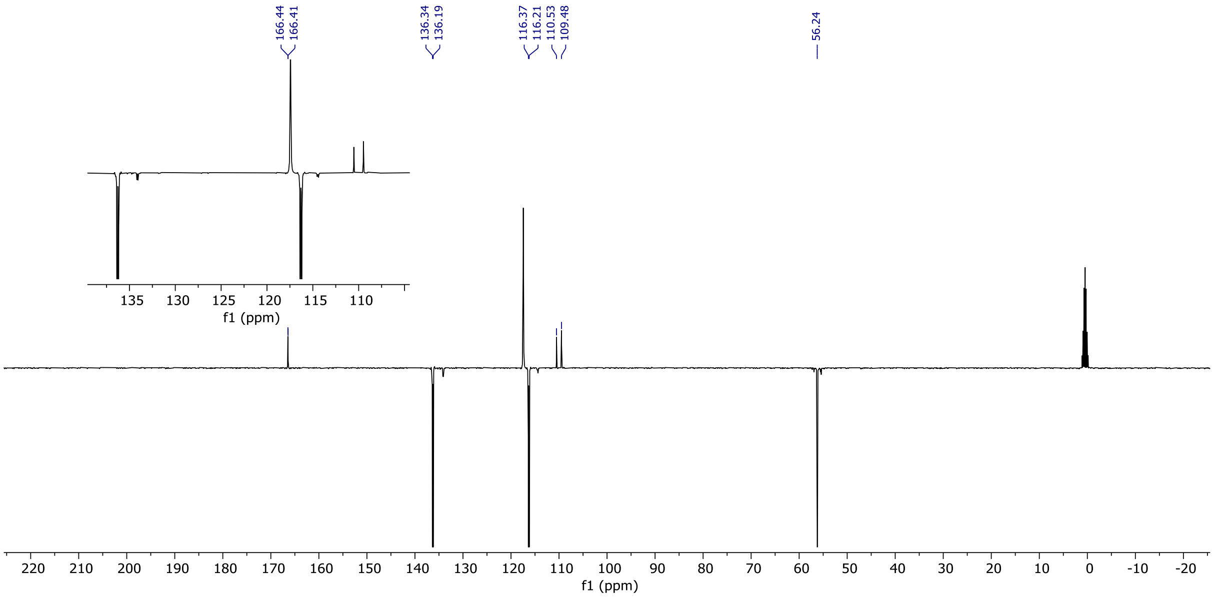

Fig. S17. *^{31}P (162 MHz), ^{1}H (400 MHz) and ^{13}C (101 MHz) NMR of* ***2c-Cl*** *in ACN-d$_3$*

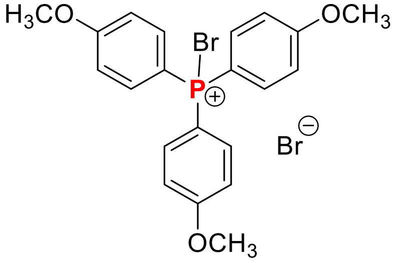

2c-Br

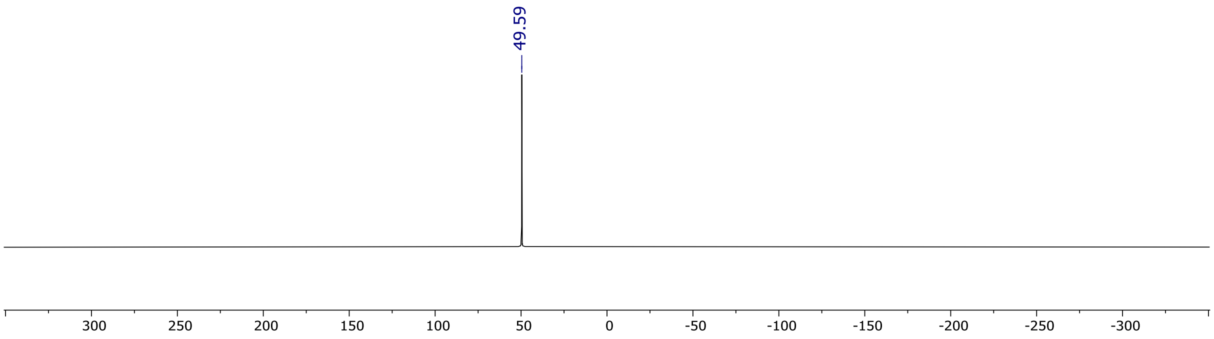

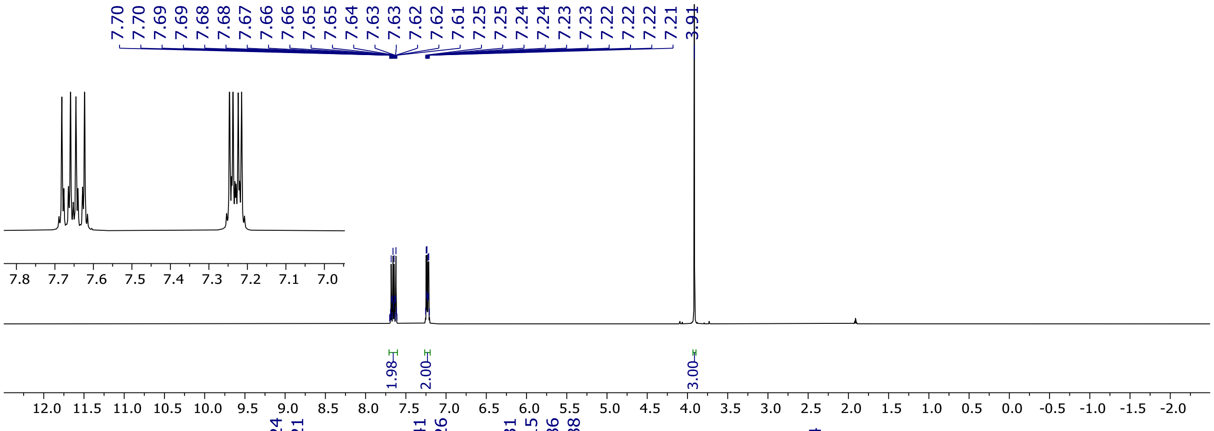

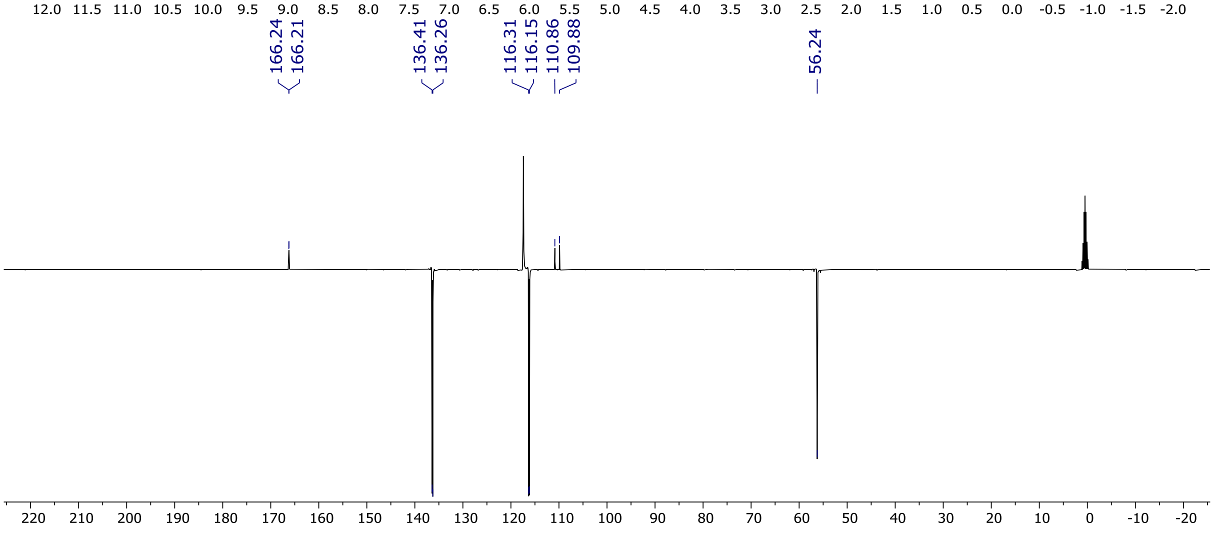

Fig. S18. *^{31}P (162 MHz), ^{1}H (400 MHz) and ^{13}C (101 MHz) NMR of **2c-Br** in ACN-d_3*

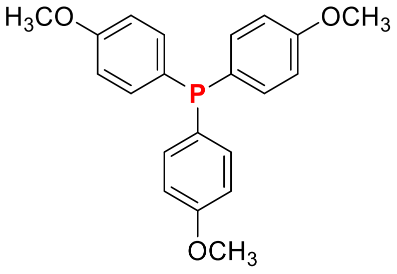

3c

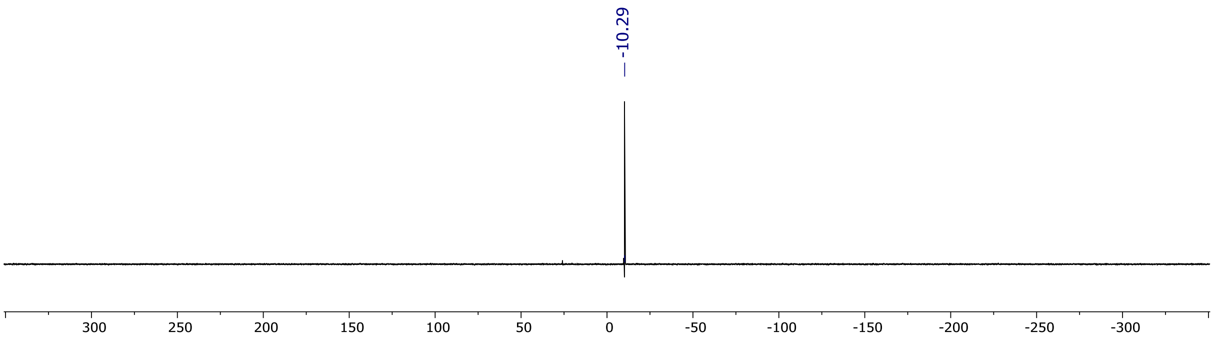

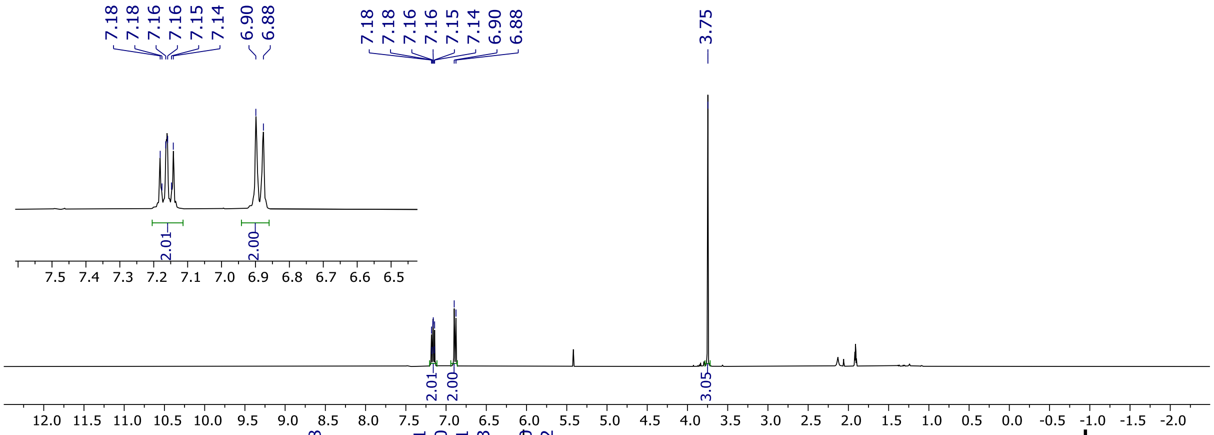

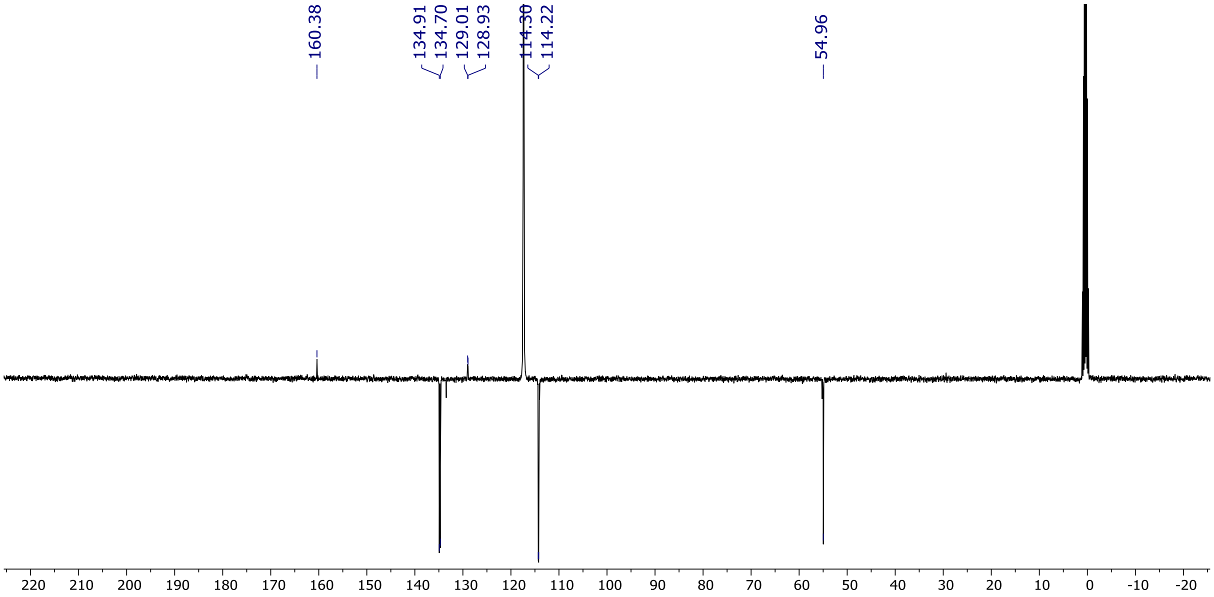

Fig. S19. *^{31}P (162 MHz), ^{1}H (400 MHz) and ^{13}C (101 MHz) NMR of **3c** in ACN-d$_3$*

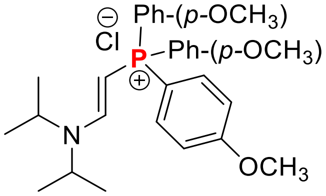

4c

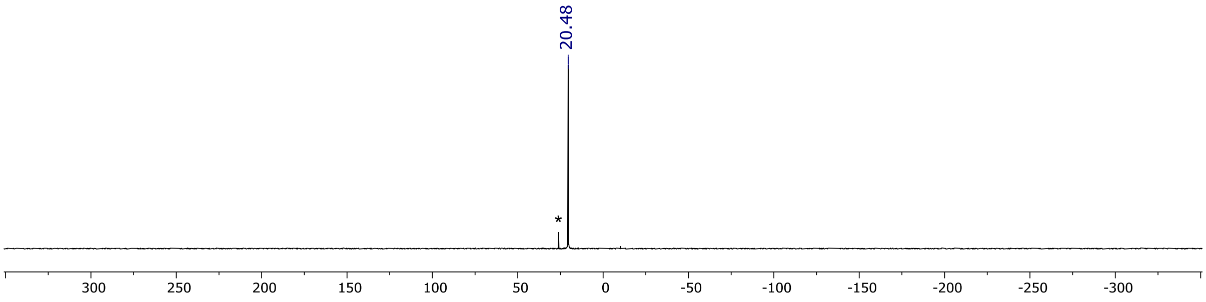

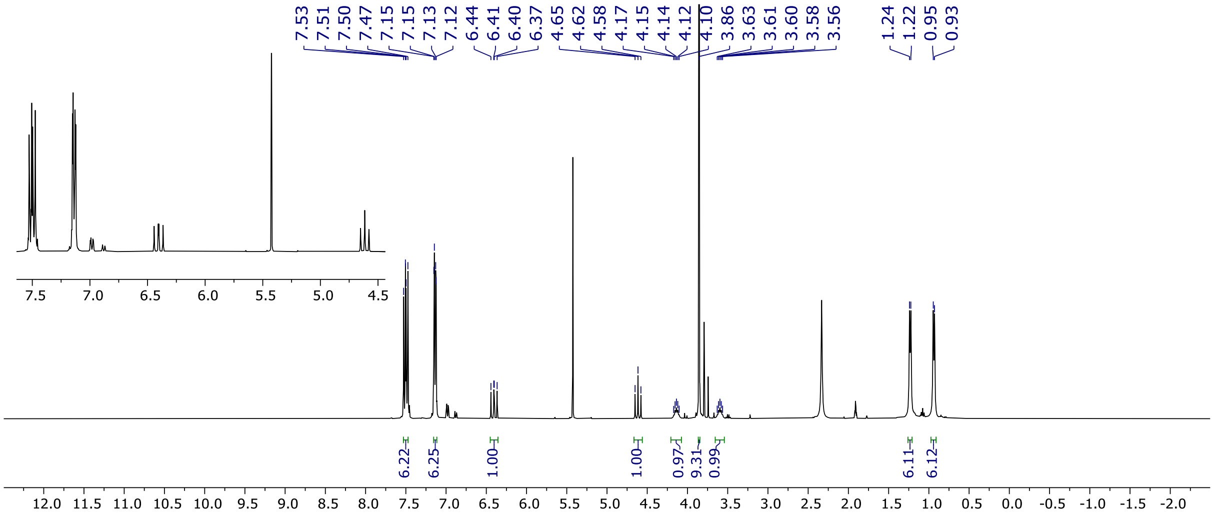

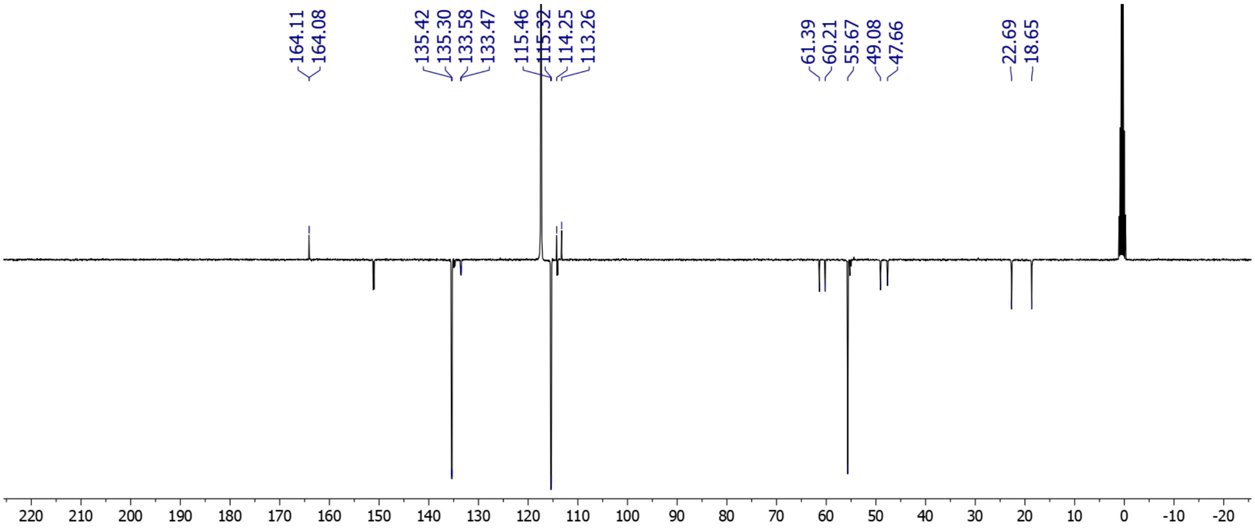

Fig. S20. *^{31}P (162 MHz), ^{1}H (400 MHz) and ^{13}C (101 MHz) NMR of* ***4c*** *in ACN-d$_3$. * marks the signal of phosphine oxide* ***1c***

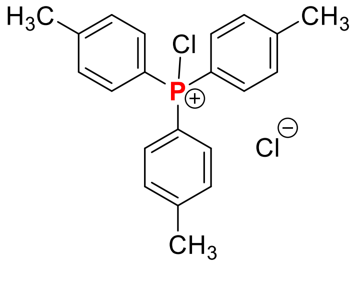

2d-Cl

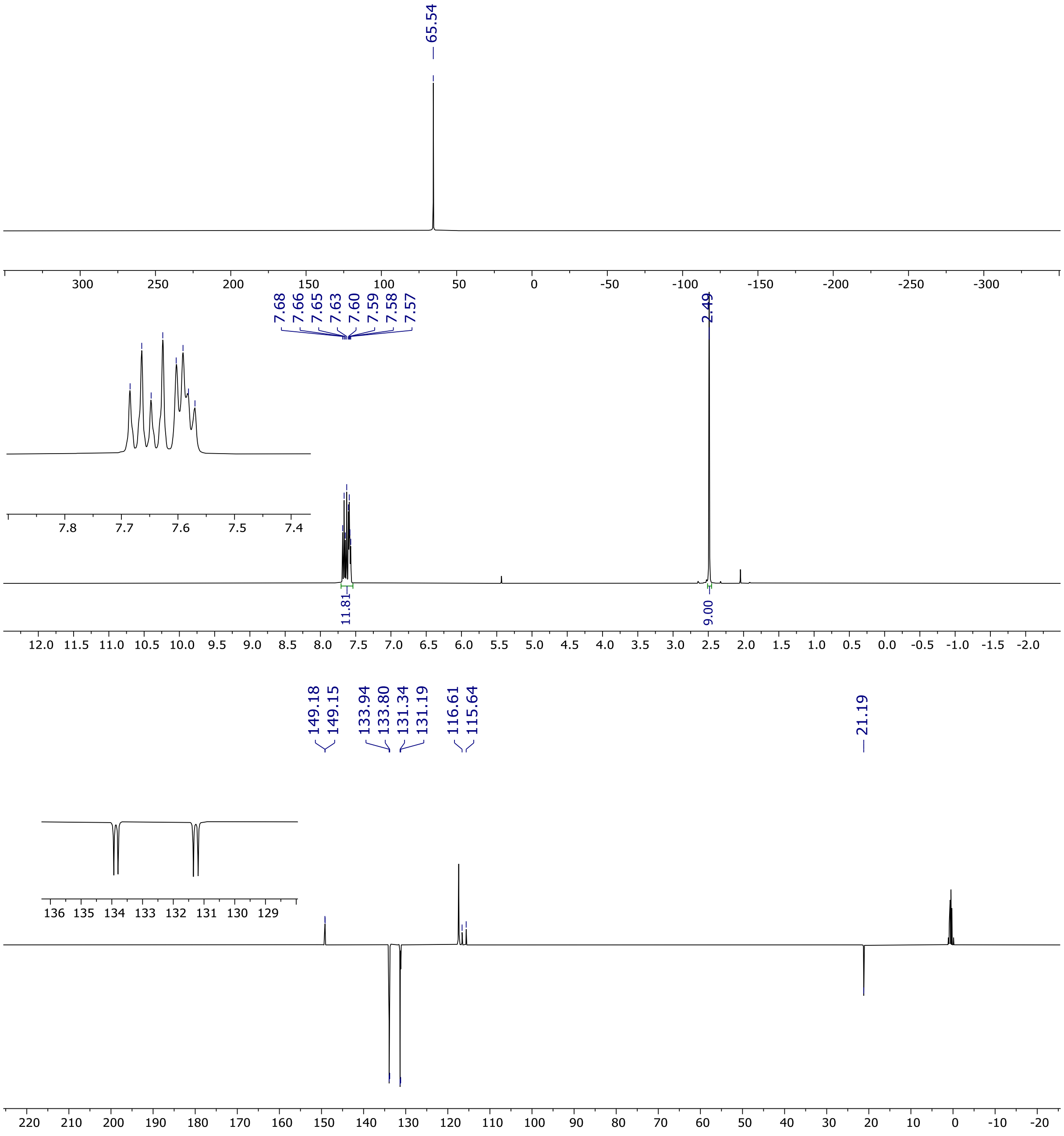

Fig. S21. *^{31}P (162 MHz), ^{1}H (400 MHz) and ^{13}C (101 MHz) NMR of* ***2d-Cl*** *in ACN-d_3*

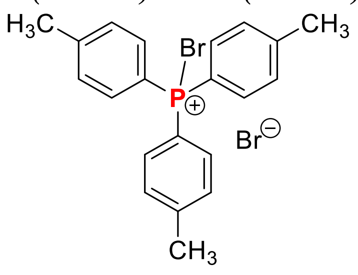

2d-Br

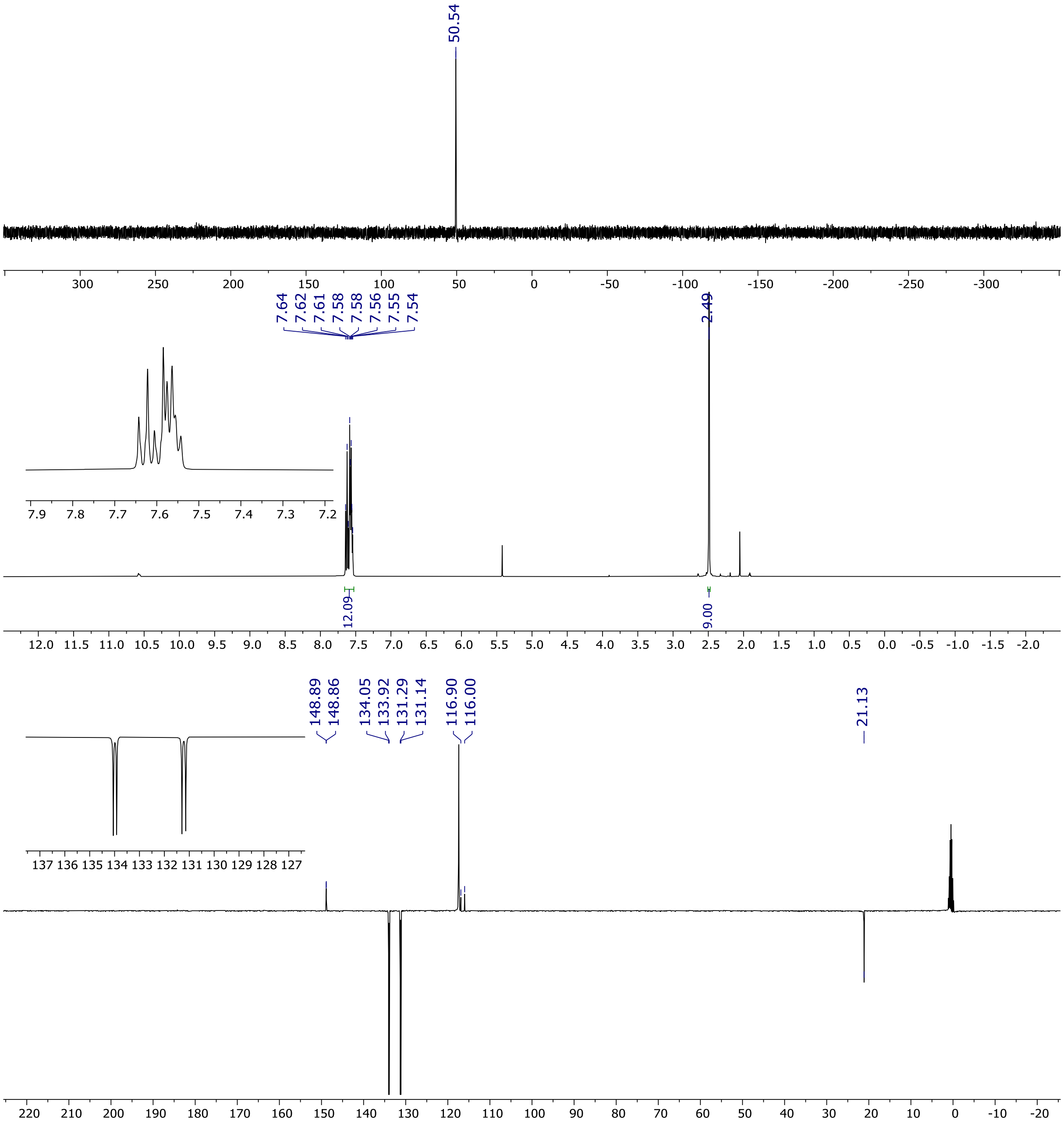

Fig. S22. *^{31}P (162 MHz), ^{1}H (400 MHz) and ^{13}C (101 MHz) NMR of **2d-Br** in ACN-d$_3$*

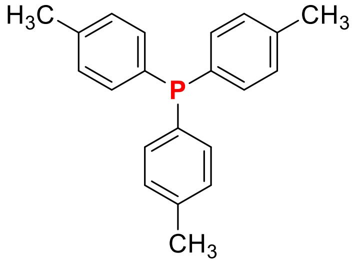

3d

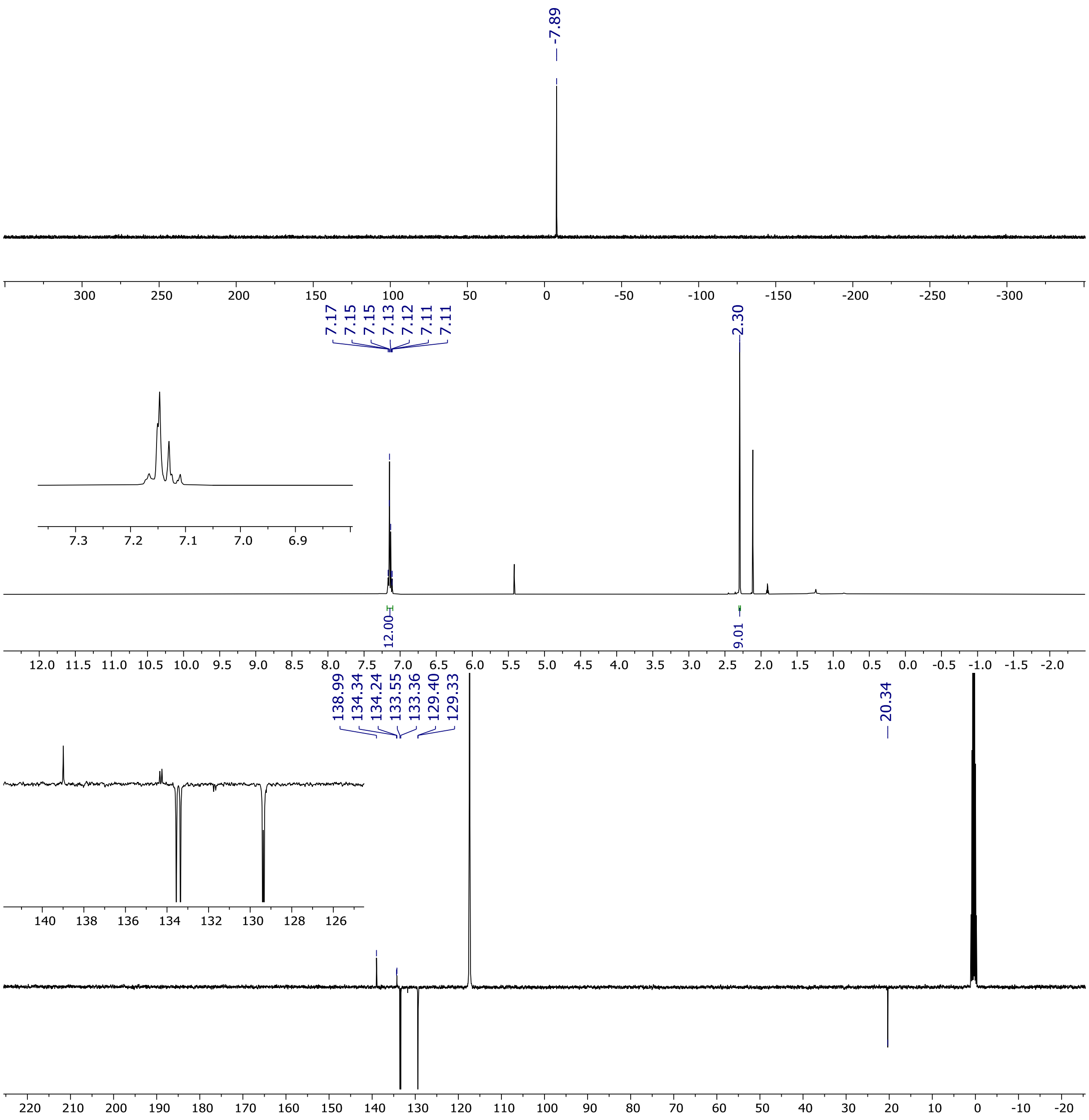

Fig. S23. *^{31}P (162 MHz), ^{1}H (400 MHz) and ^{13}C (101 MHz) NMR of **3d** in ACN-d$_3$*

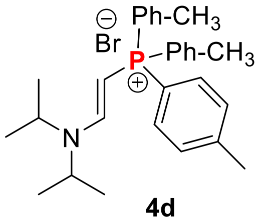

4d

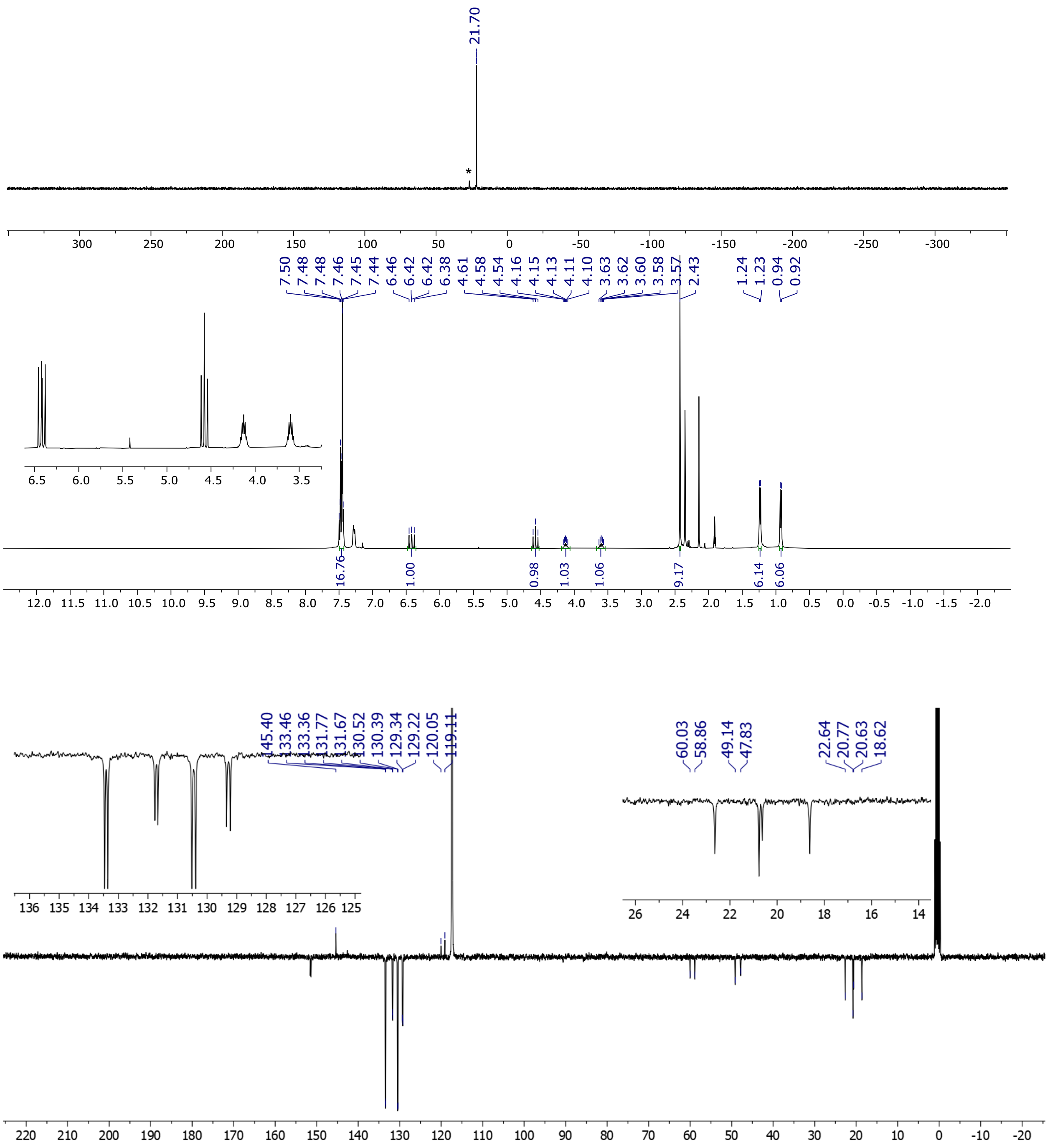

Fig. S24. *^{31}P (162 MHz), ^{1}H (400 MHz) and ^{13}C (101 MHz) NMR of **4d** in ACN-d₃. * marks the signal of phosphine oxide **1d***

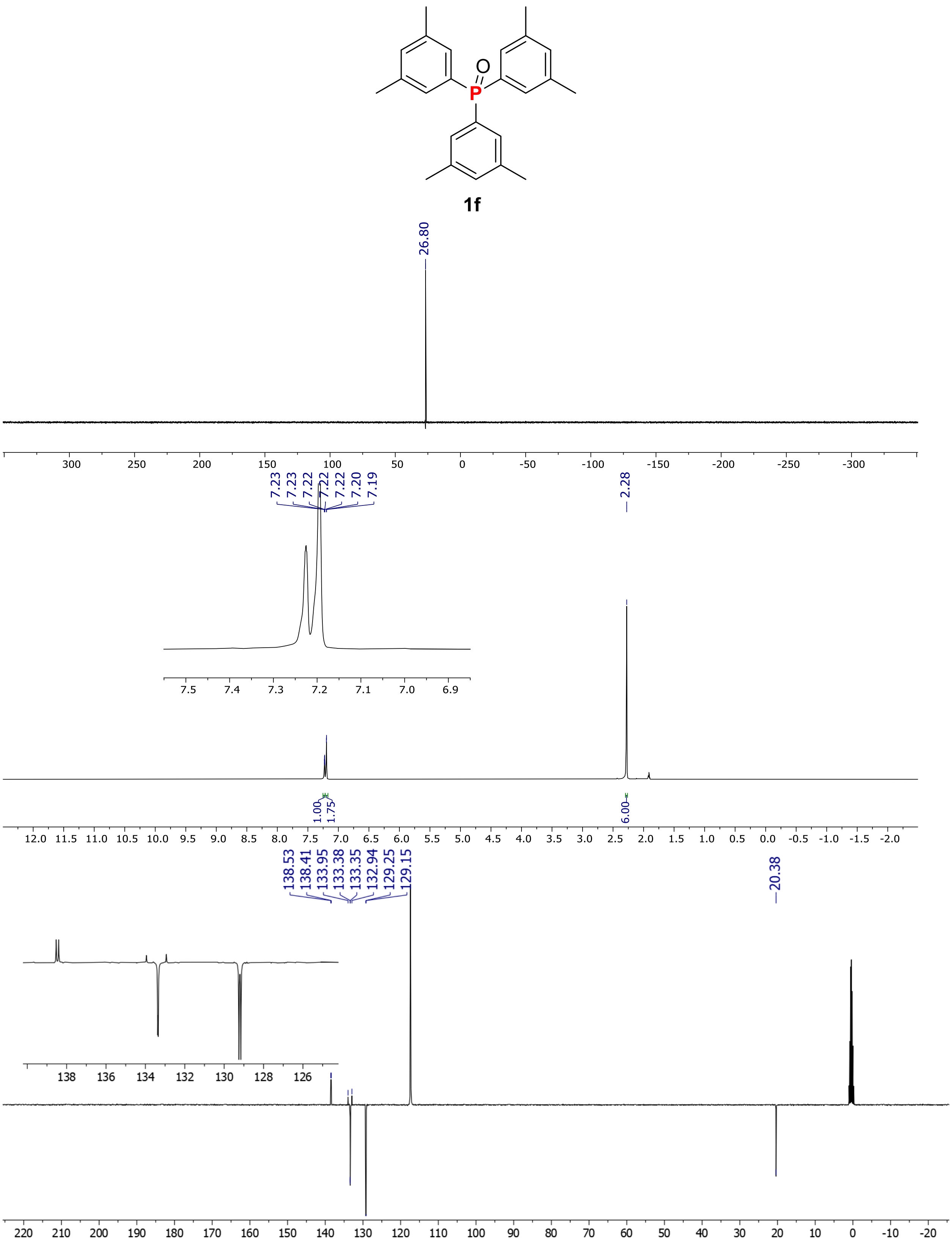

Fig. S25. *^{31}P (162 MHz), ^{1}H (400 MHz) and ^{13}C (101 MHz) NMR of **1f** in ACN-d_3*

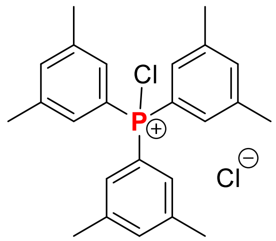

2f-Cl

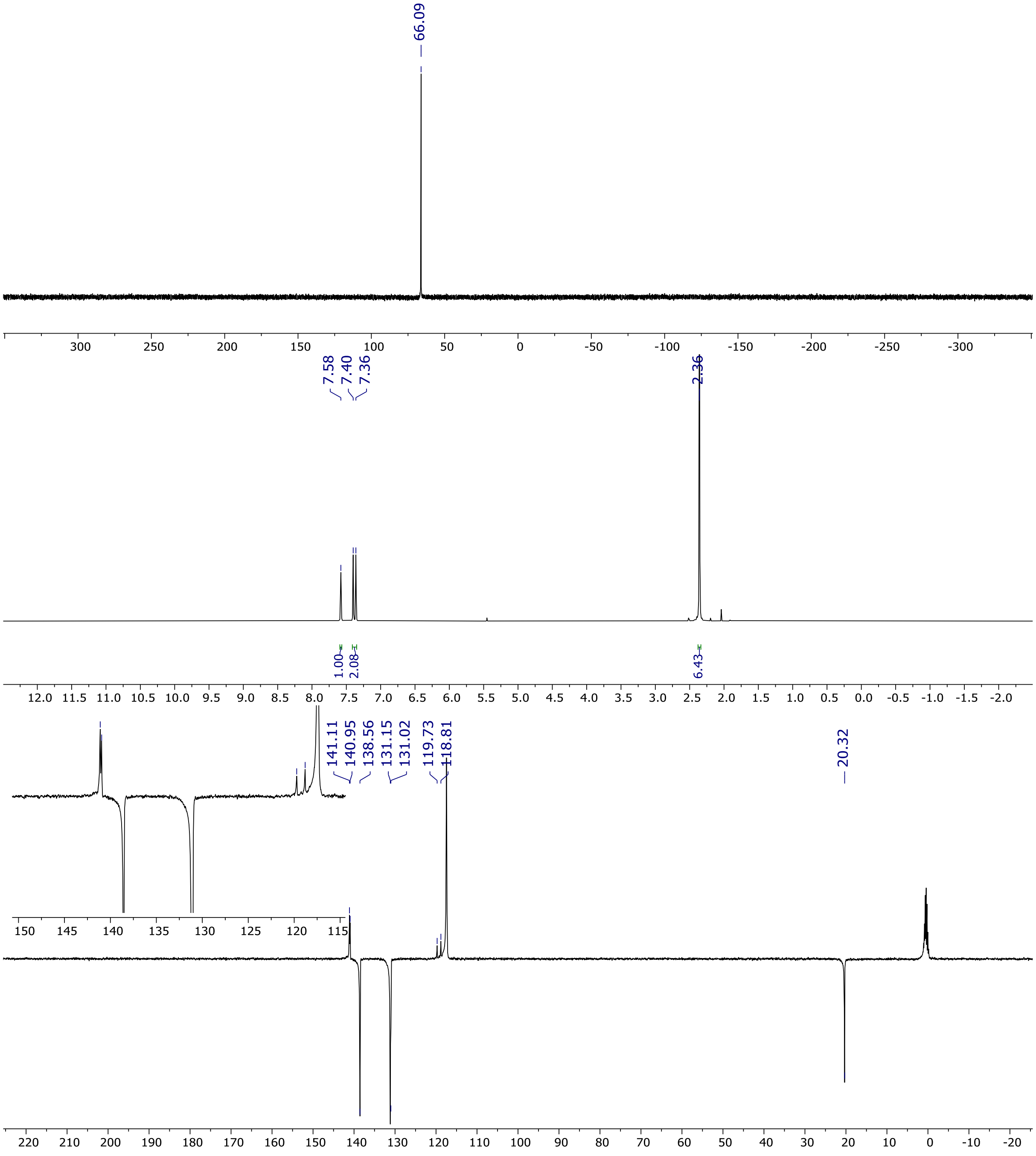

Fig. S26. *^{31}P (162 MHz), ^{1}H (400 MHz) and ^{13}C (101 MHz) NMR of* ***2f-Cl*** *in ACN-d_3*

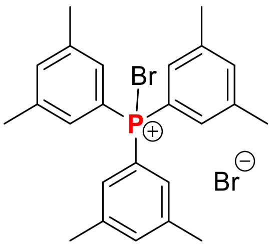

2f-Br

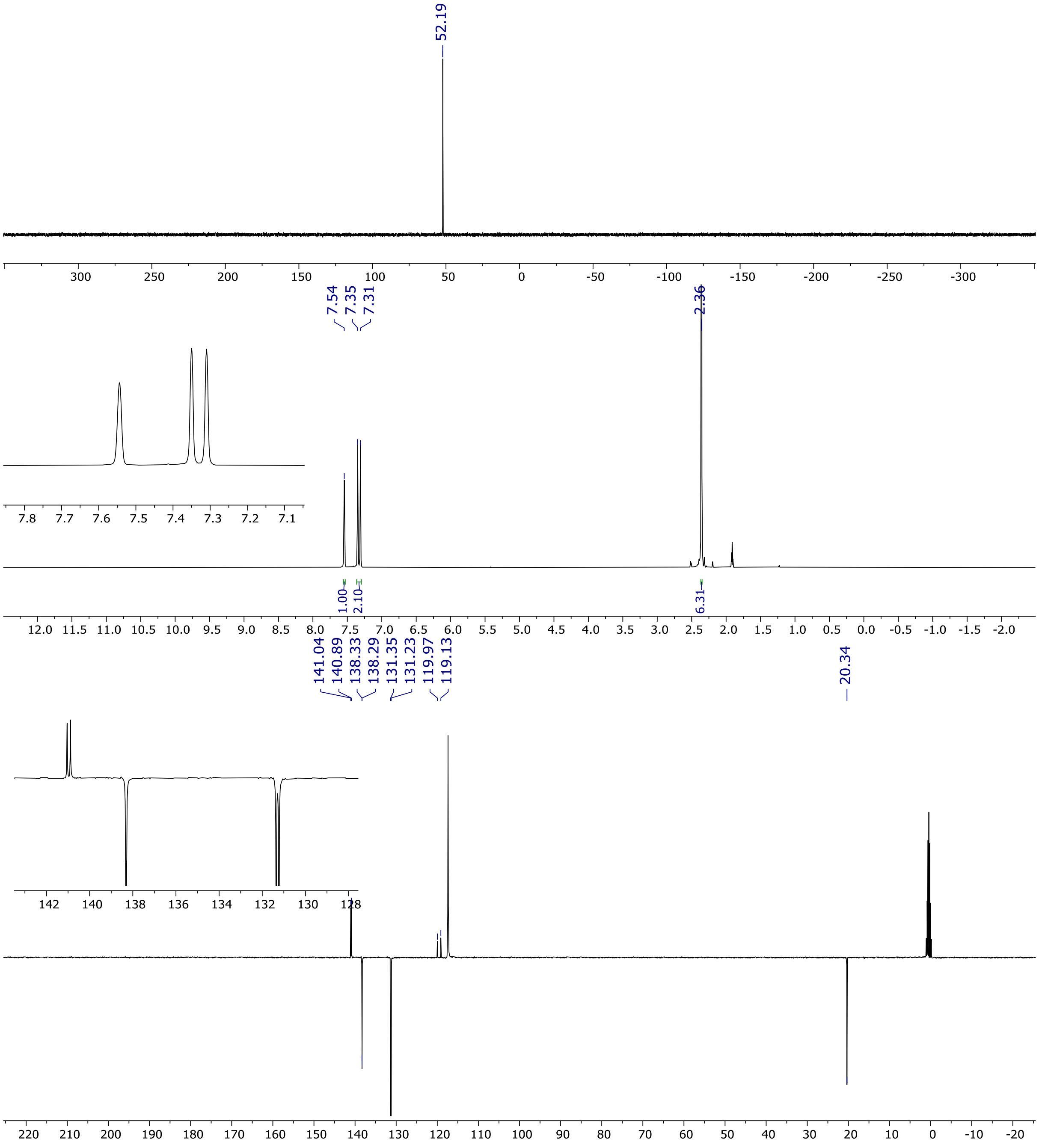

Fig. S27. *^{31}P (162 MHz), ^{1}H (400 MHz) and ^{13}C (101 MHz) NMR of* ***2f-Br*** *in ACN-d_3*

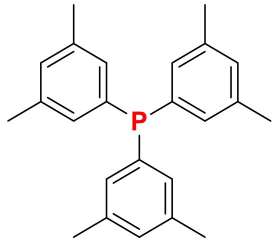

3f

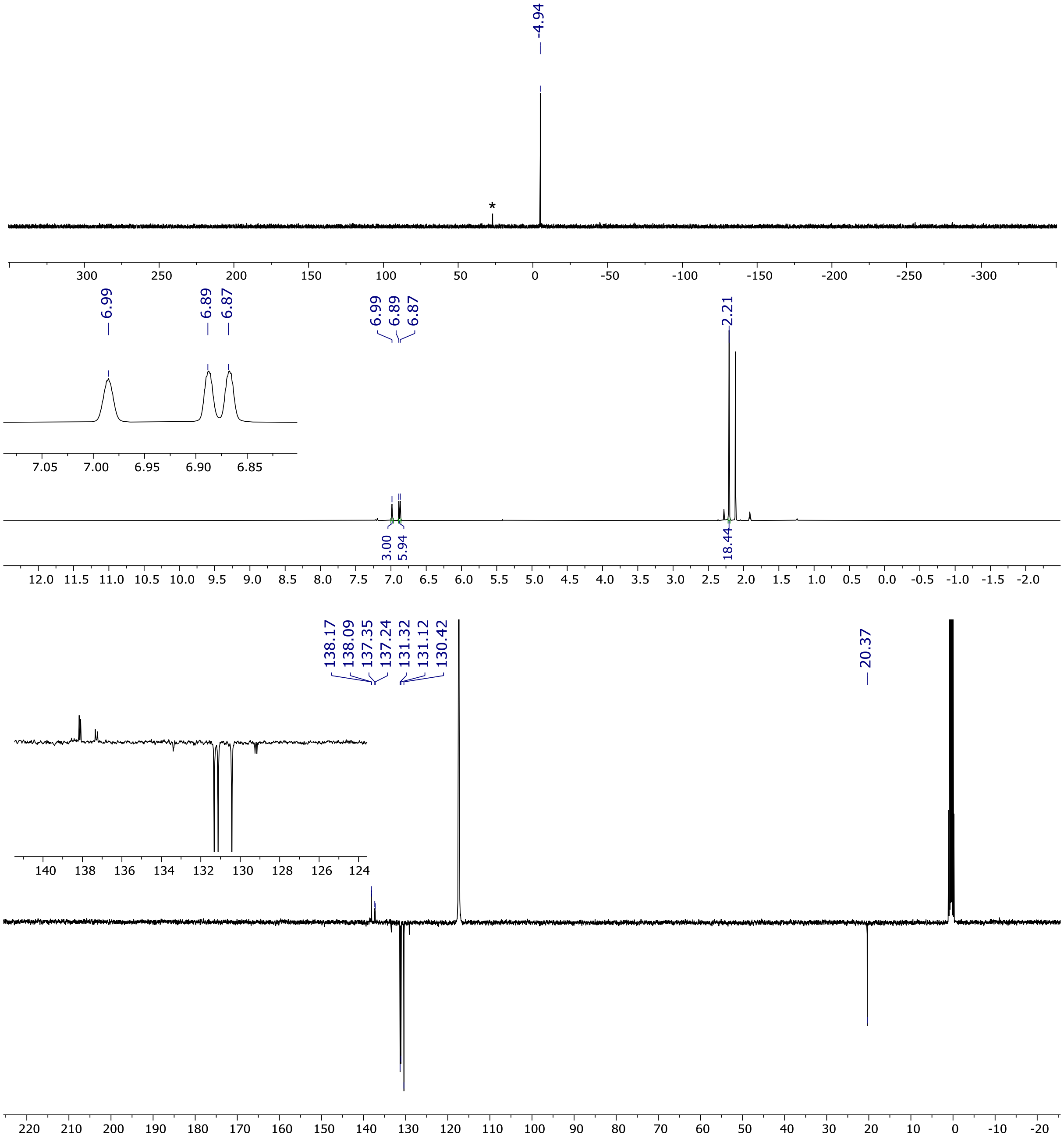

Fig. S28. *^{31}P (162 MHz), ^{1}H (400 MHz) and ^{13}C (101 MHz) NMR of* ***3f*** *in ACN-d$_3$. * marks the signal of phosphine oxide* ***1f***

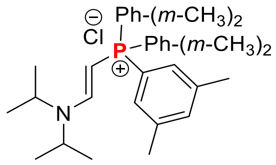

4f

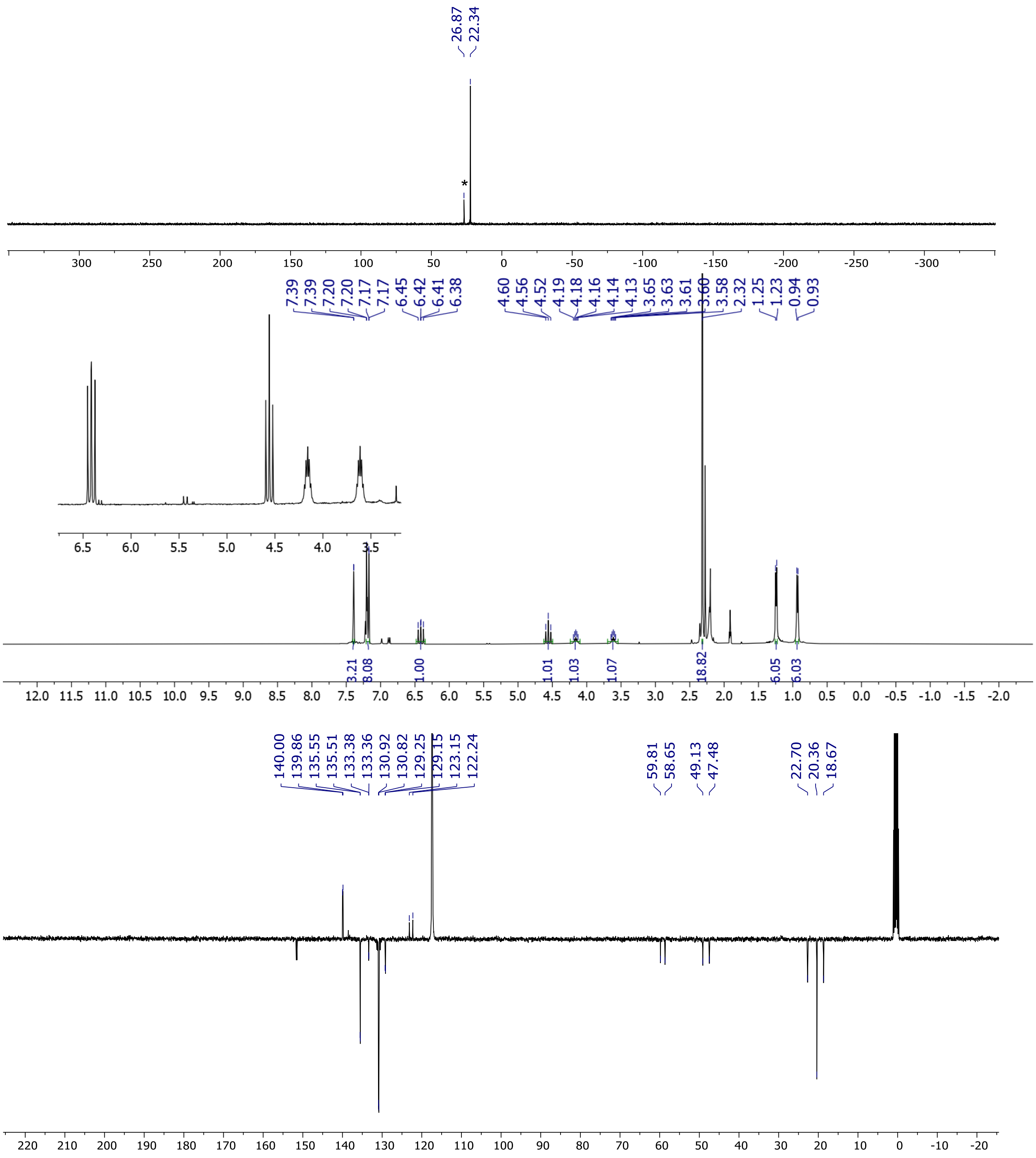

Fig. S29. ^{31}P (162 MHz), ^{1}H (400 MHz) and ^{13}C (101 MHz) NMR of **4e** in ACN-d_3. * marks the signal of phosphine oxide ***1f***

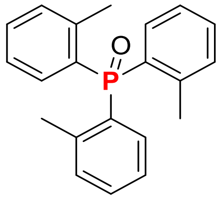

1e

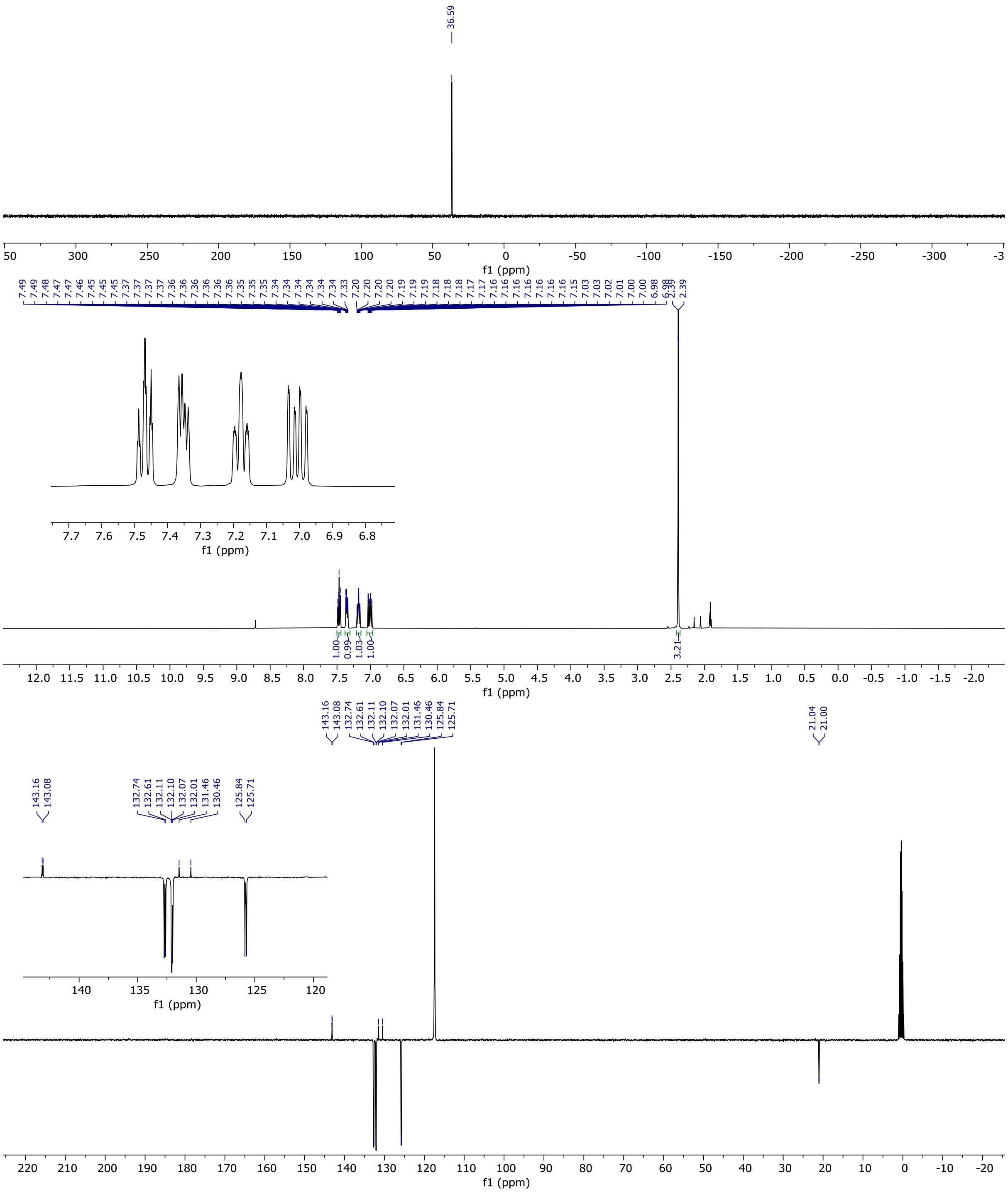

Fig. S30. *^{31}P (162 MHz), ^{1}H (400 MHz) and ^{13}C (101 MHz) NMR of **1e** in ACN-d_3*

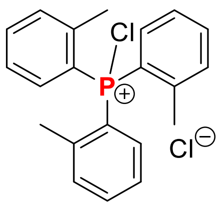

2e-Cl

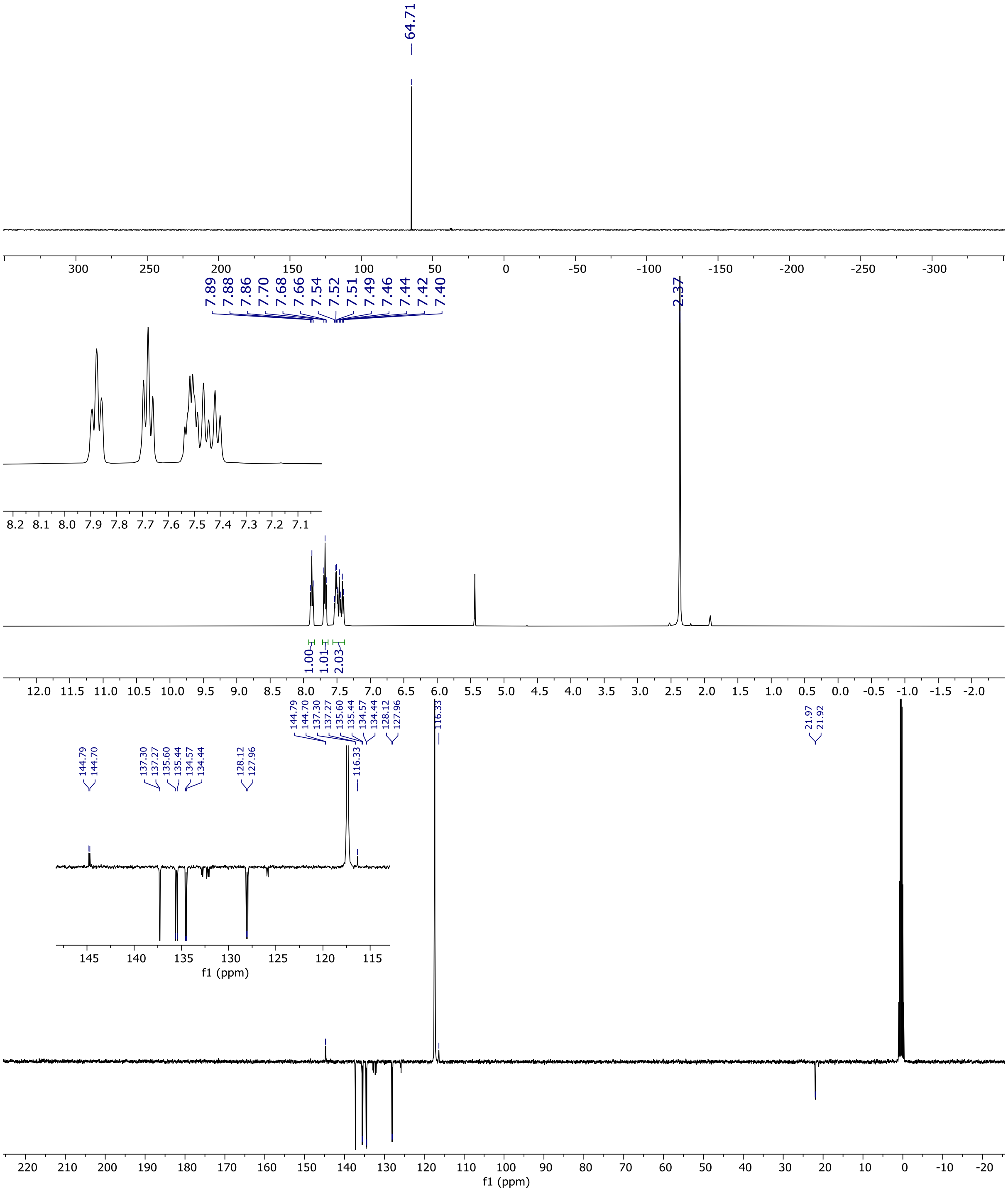

Fig. S31. ^{31}P (162 MHz), ^{1}H (400 MHz) and ^{13}C (101 MHz) NMR of ***2e-Cl*** in ACN-d_3

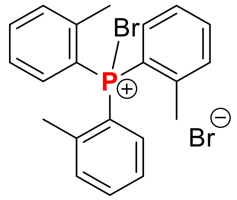

2e-Br

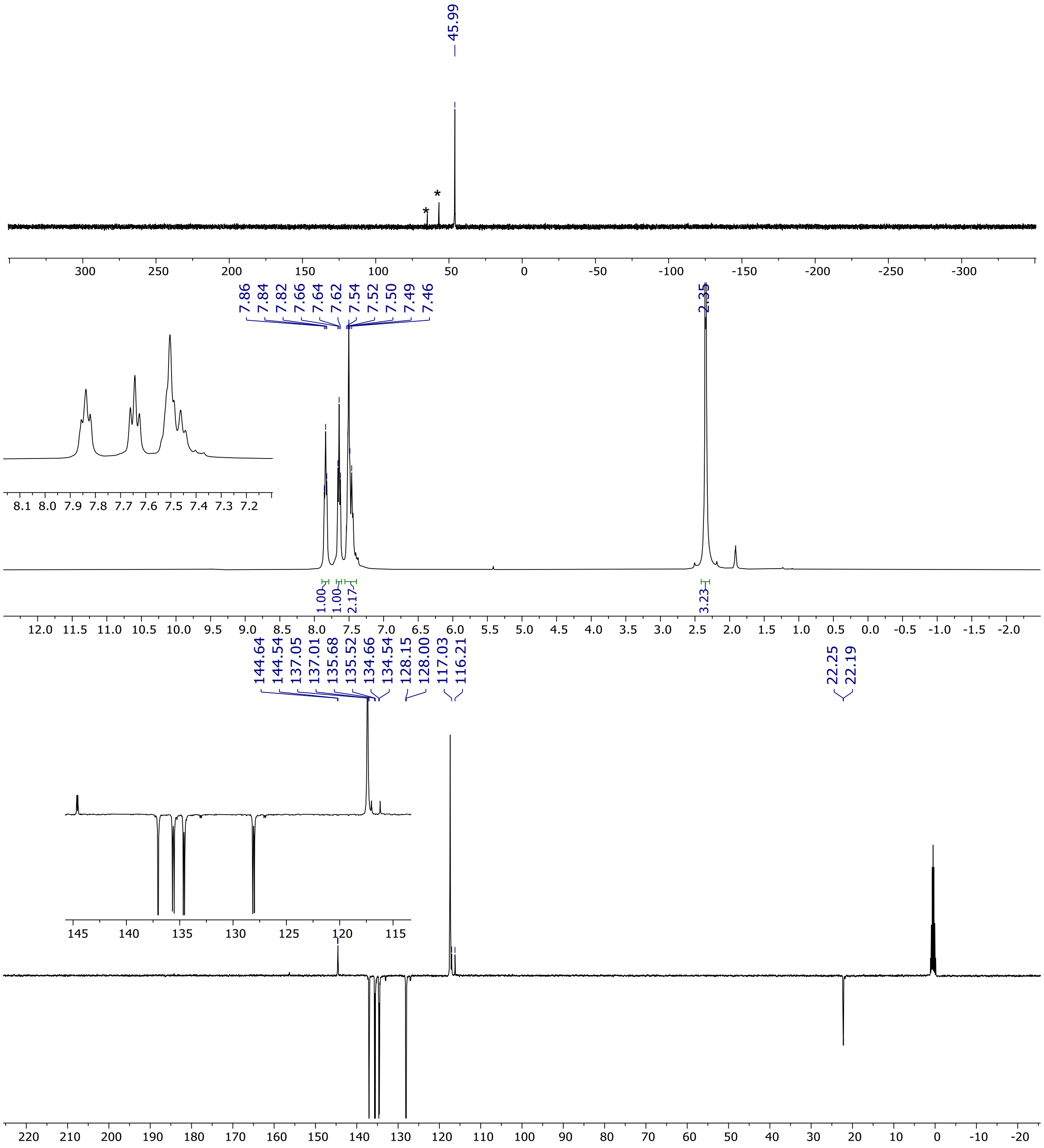

Fig. S32. ^{31}P (162 MHz), ^{1}H (400 MHz) and ^{13}C (101 MHz) NMR of **2e-Br** in ACN-d_3. * marks the signal of unknown product.

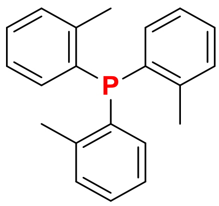

3e

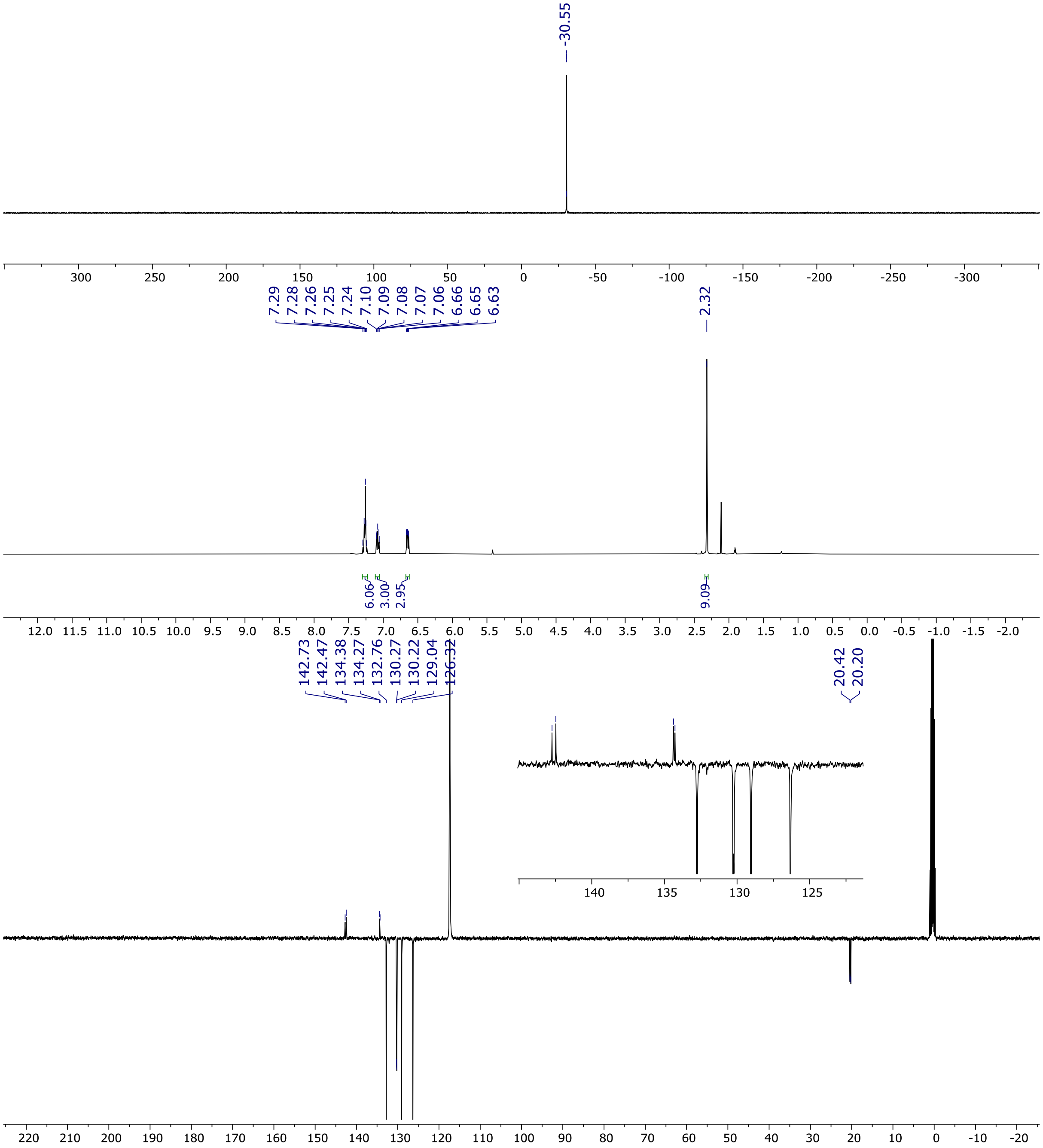

Fig. S33. *^{31}P (162 MHz), ^{1}H (400 MHz) and ^{13}C (101 MHz) NMR of **3e** in ACN-d$_3$*

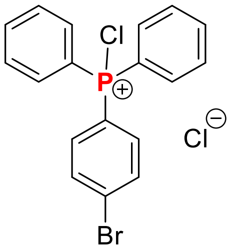

2h-Cl

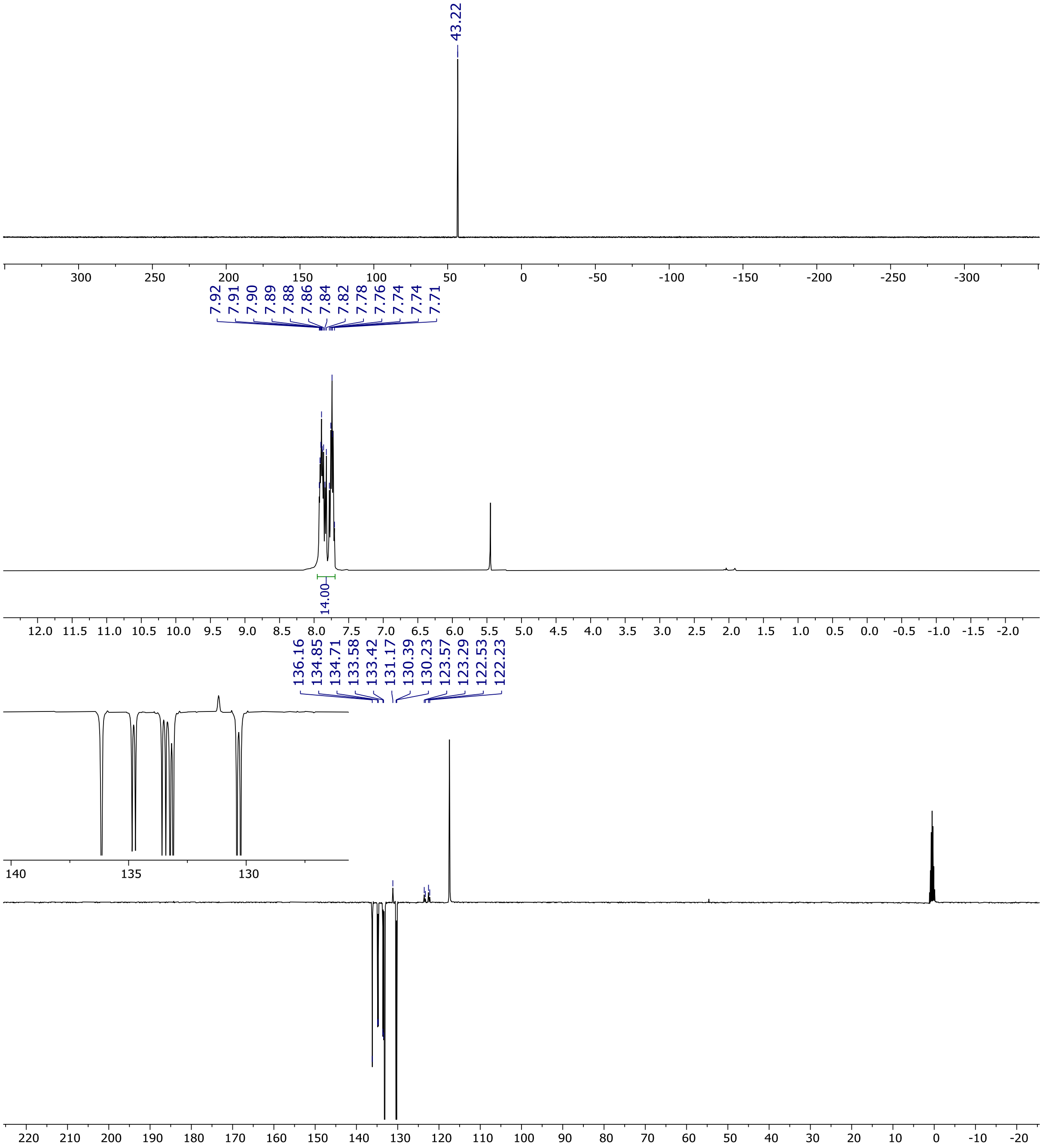

Fig. S34. *^{31}P (162 MHz), ^{1}H (400 MHz) and ^{13}C (101 MHz) NMR of **2h-Cl** in ACN-d_3*

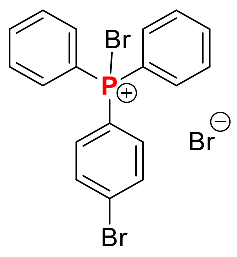

2h-Br

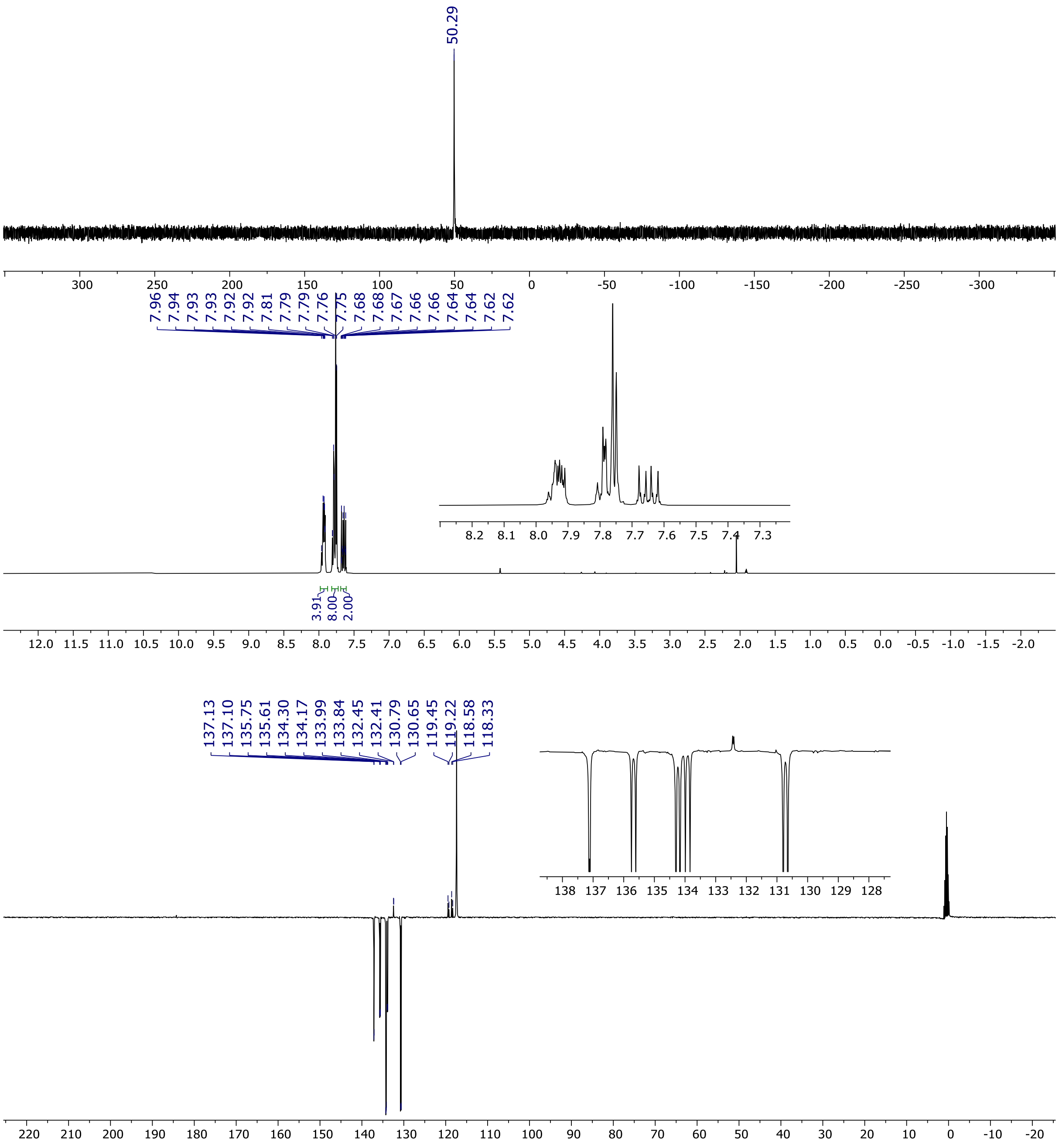

***Fig. S35**. ^{31}P (162 MHz), ^{1}H (400 MHz) and ^{13}C (101 MHz) NMR of **2h-Br** in ACN-d_3*

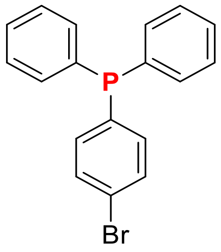

3h

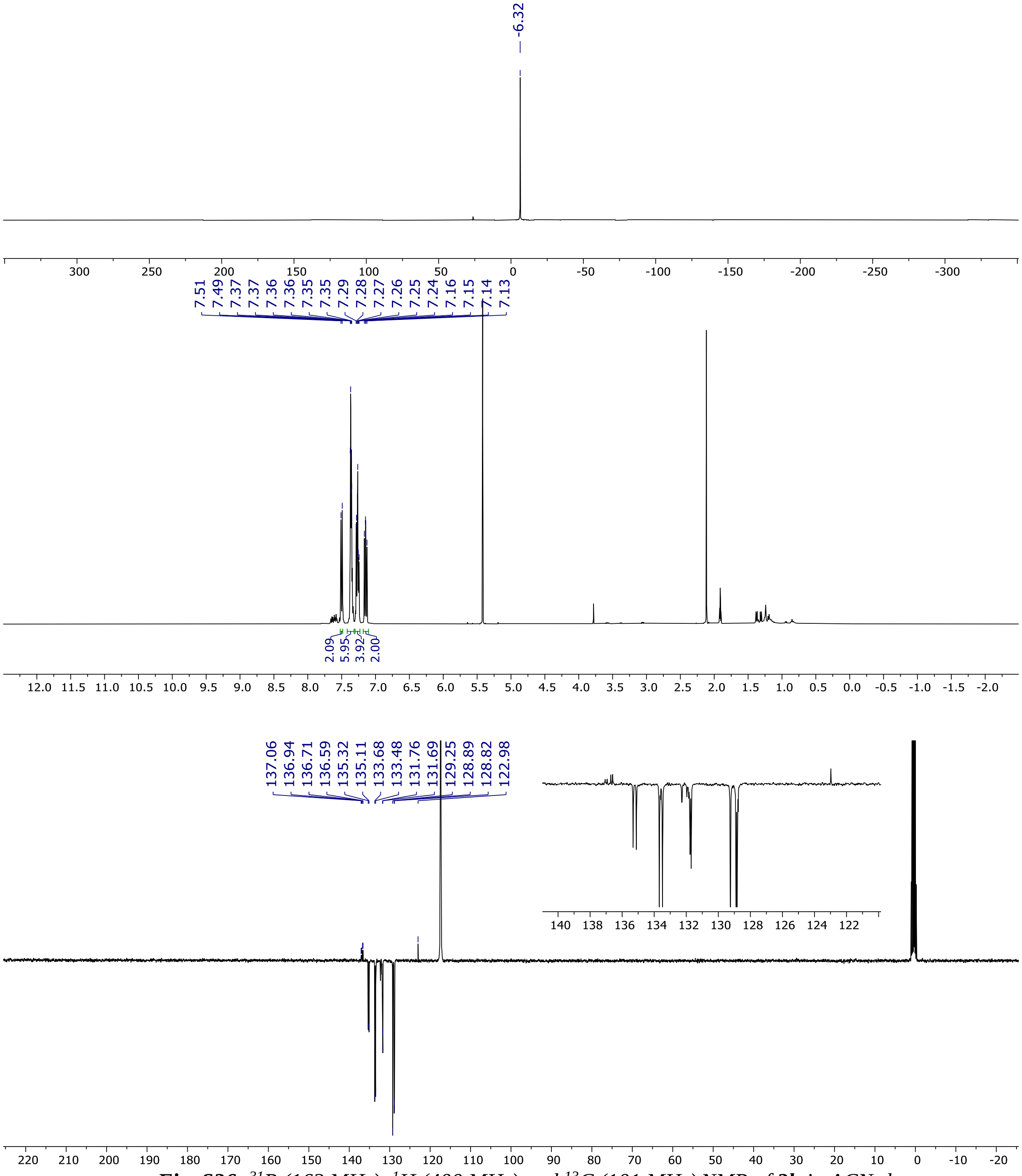

Fig. S36. *^{31}P (162 MHz), ^{1}H (400 MHz) and ^{13}C (101 MHz) NMR of **3h** in ACN-d_3*

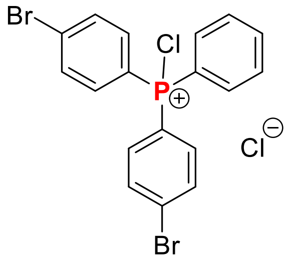

2i-Cl

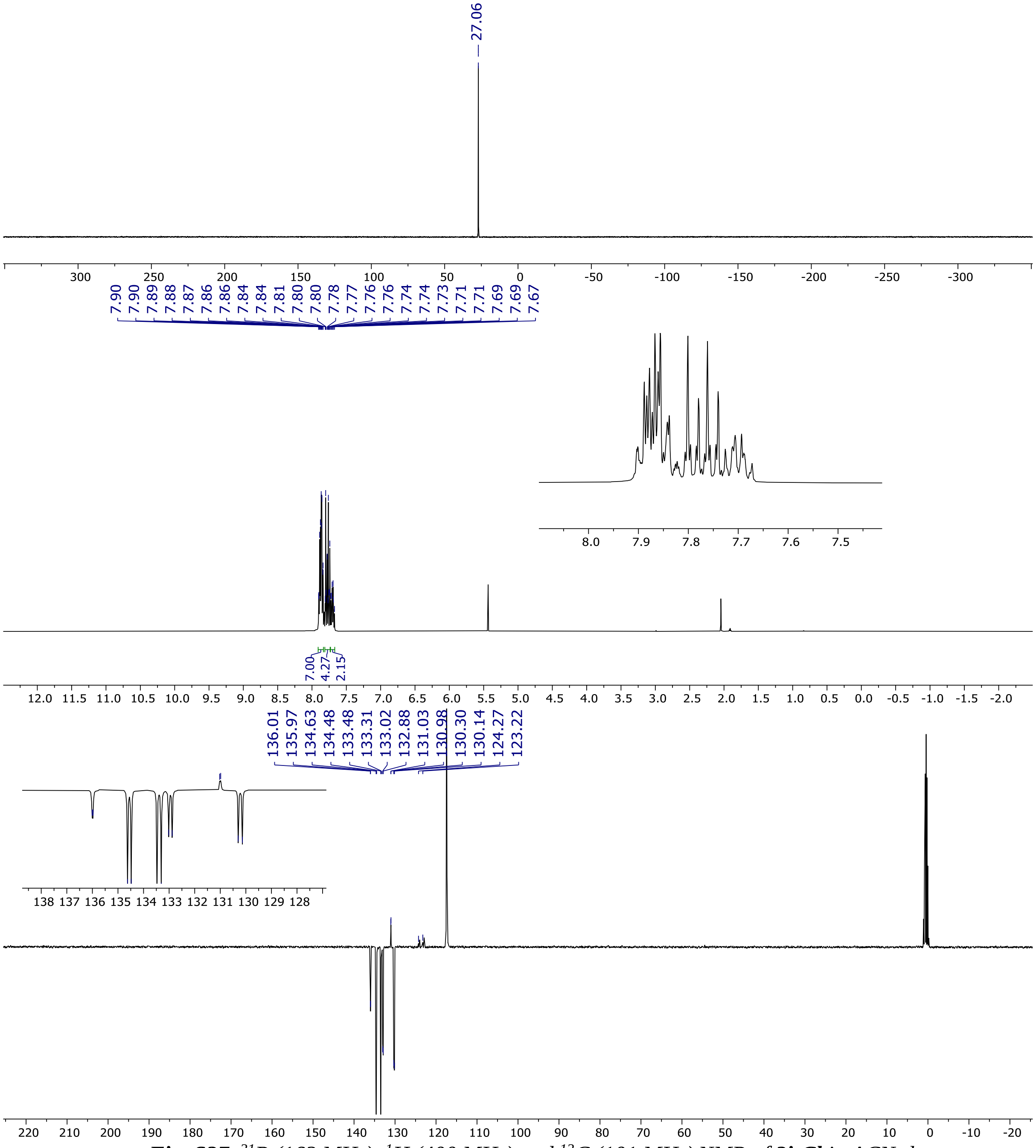

Fig. S37. ^{31}P (162 MHz), ^{1}H (400 MHz) and ^{13}C (101 MHz) NMR of **2i-Cl** in ACN-d_3

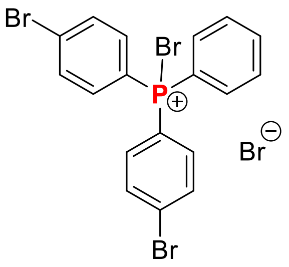

2i-Br

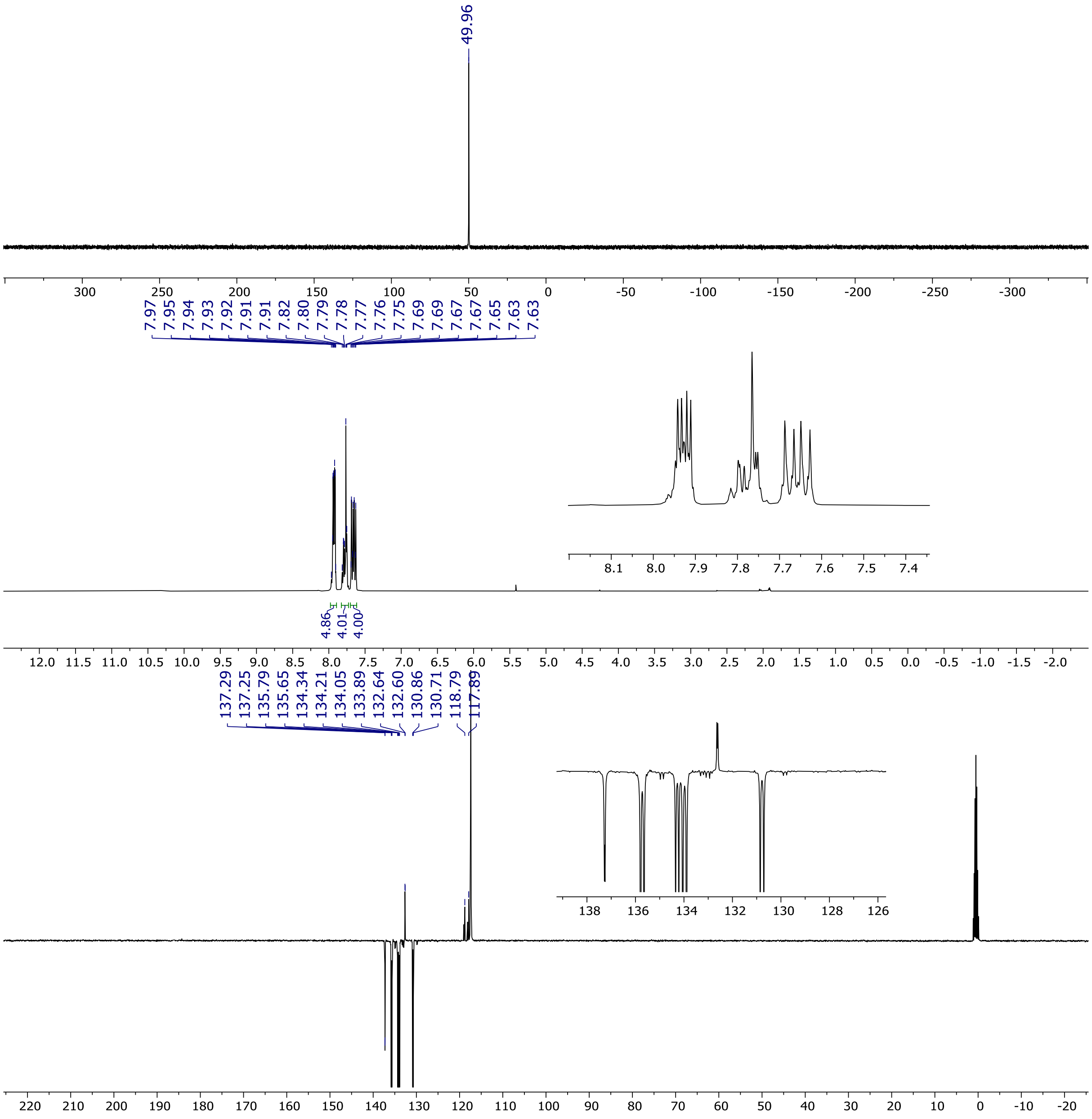

Fig. S38. *^{31}P (162 MHz), ^{1}H (400 MHz) and ^{13}C (101 MHz) NMR of* ***2i-Br*** *in ACN-d_3*

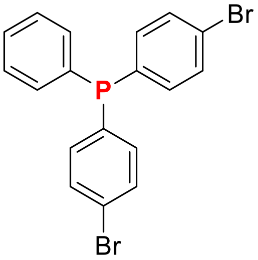

3i

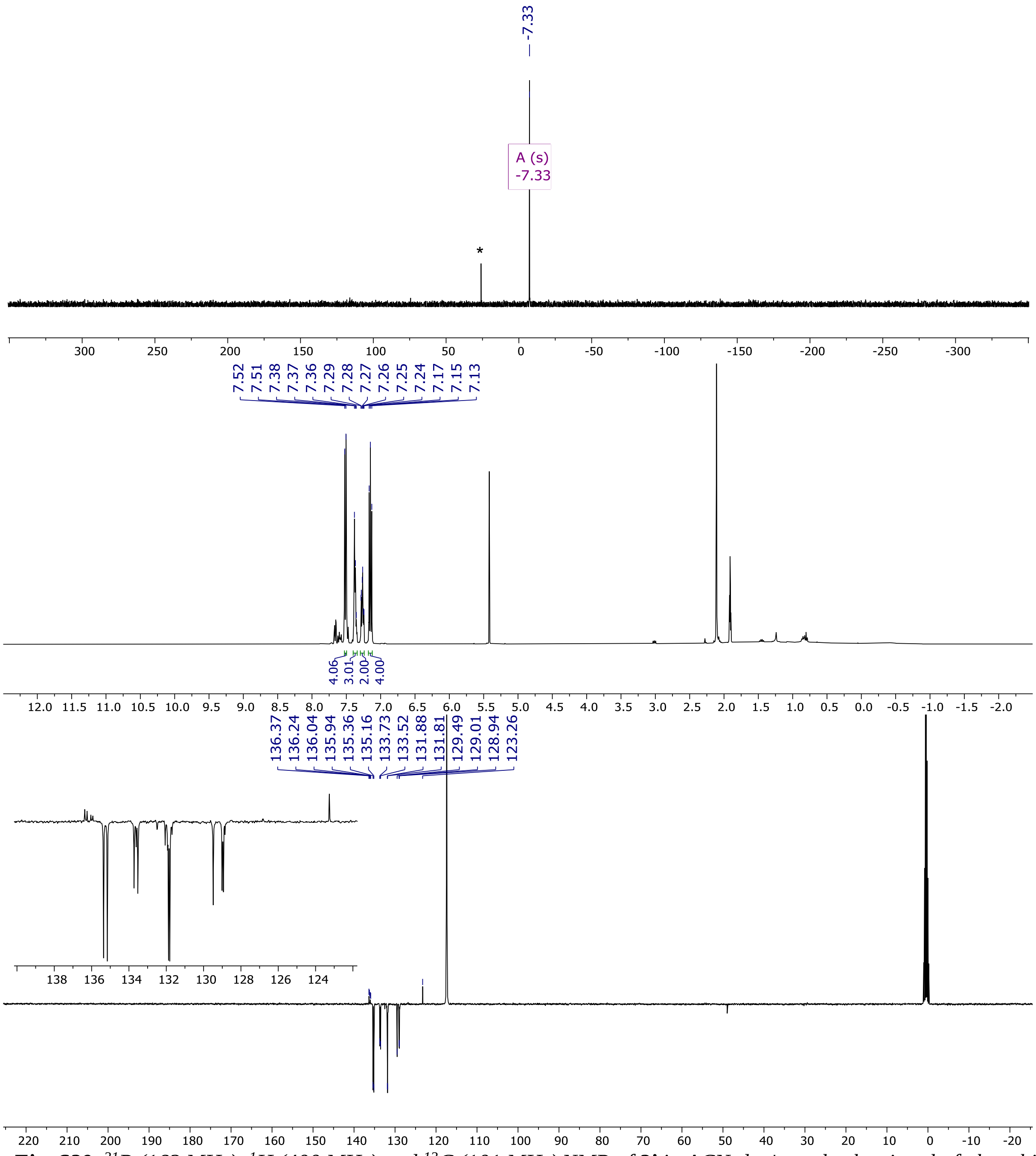

Fig. S39. *^{31}P (162 MHz), ^{1}H (400 MHz) and ^{13}C (101 MHz) NMR of **3i** in ACN-d_3. * marks the signal of phosphine oxide **1i***

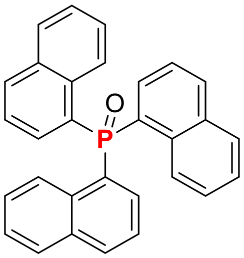

1g

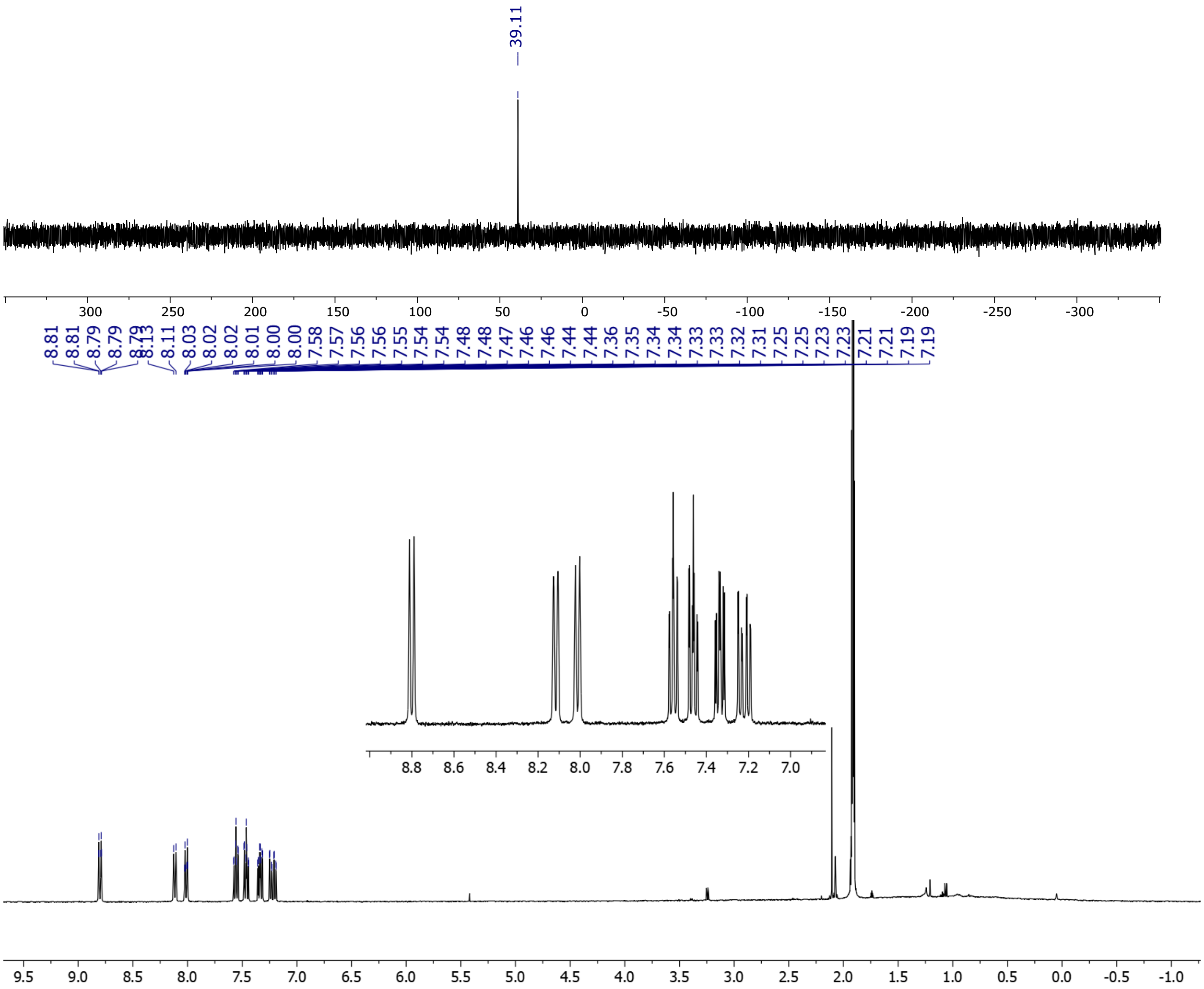

Fig. S40. *^{31}P (162 MHz) and ^{1}H (400 MHz) NMR of* ***1g*** *in ACN-d_3*

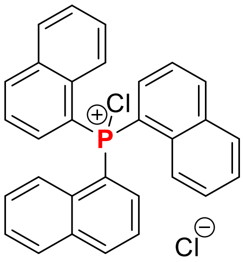

2g-Cl

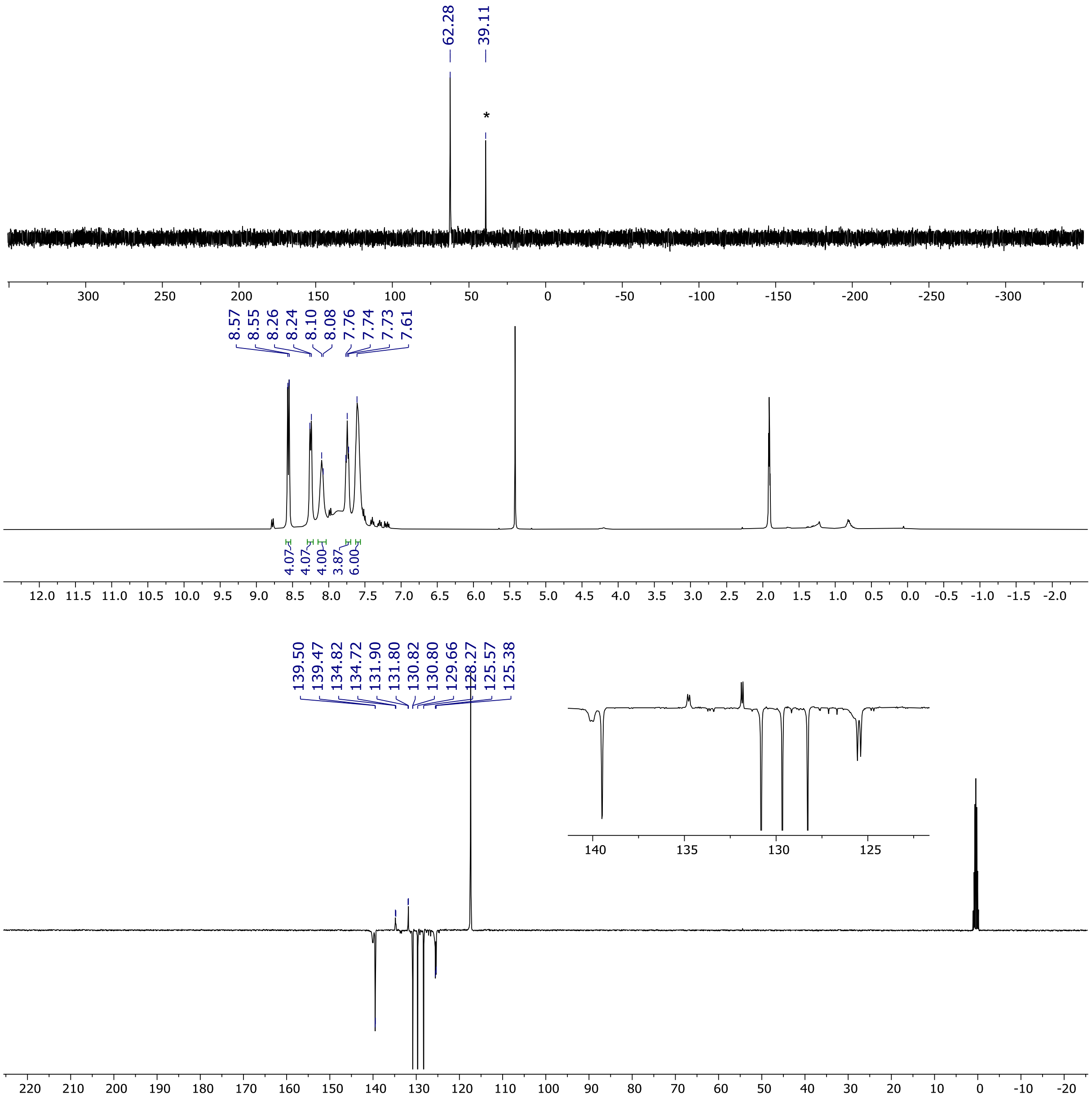

Fig. S41. *^{31}P (162 MHz), ^{1}H (400 MHz) and ^{13}C (101 MHz) NMR of* ***2g-Cl*** *in ACN-d_3. * marks the signal of phosphine oxide* ***1g***

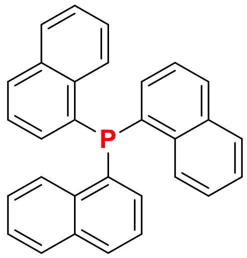

3g

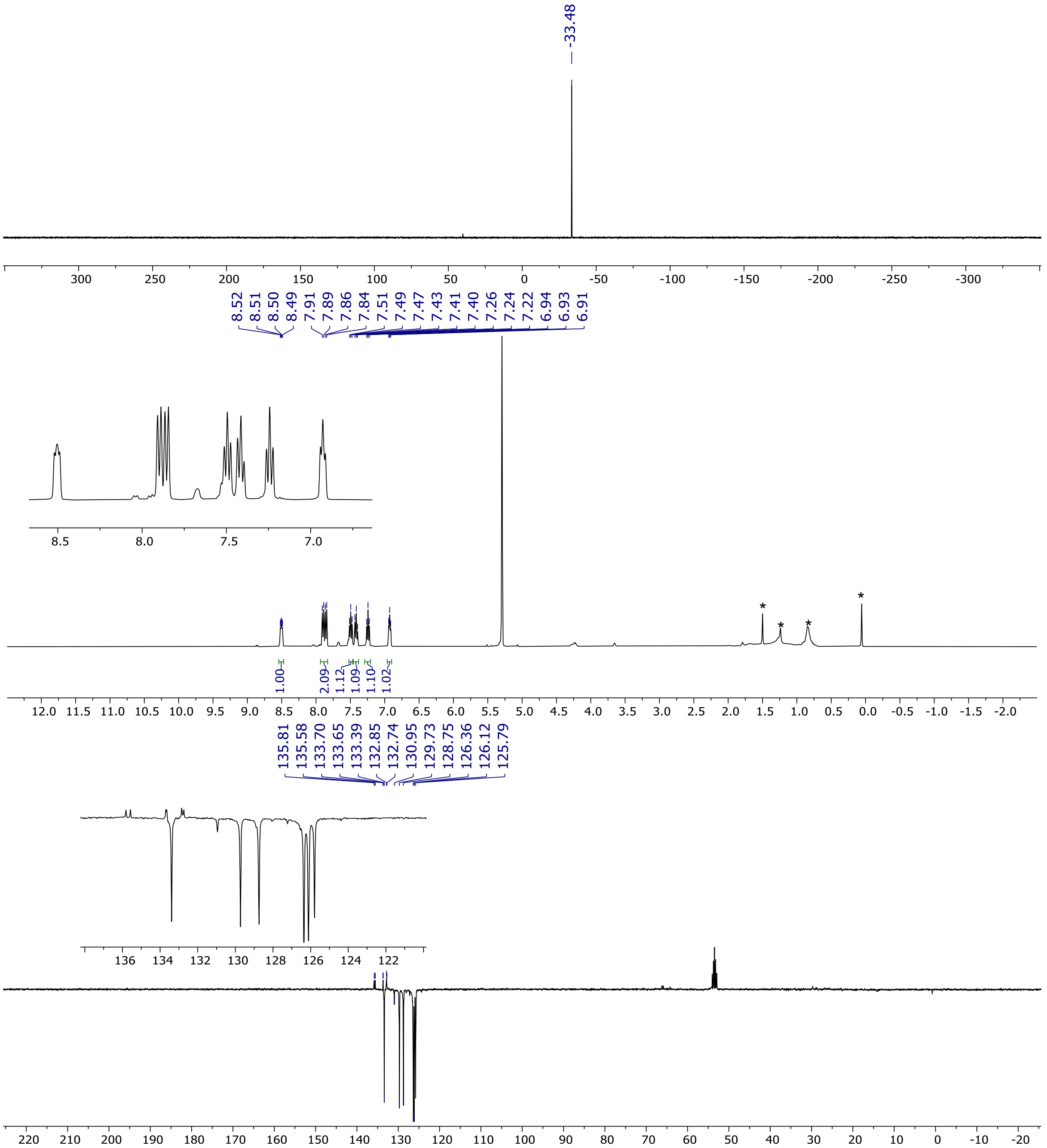

Fig. S42. *^{31}P (162 MHz), ^{1}H (400 MHz) and ^{13}C (101 MHz) NMR of **3g** in ACN-d_3. * marks the signal of impurity*

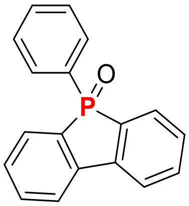

1j

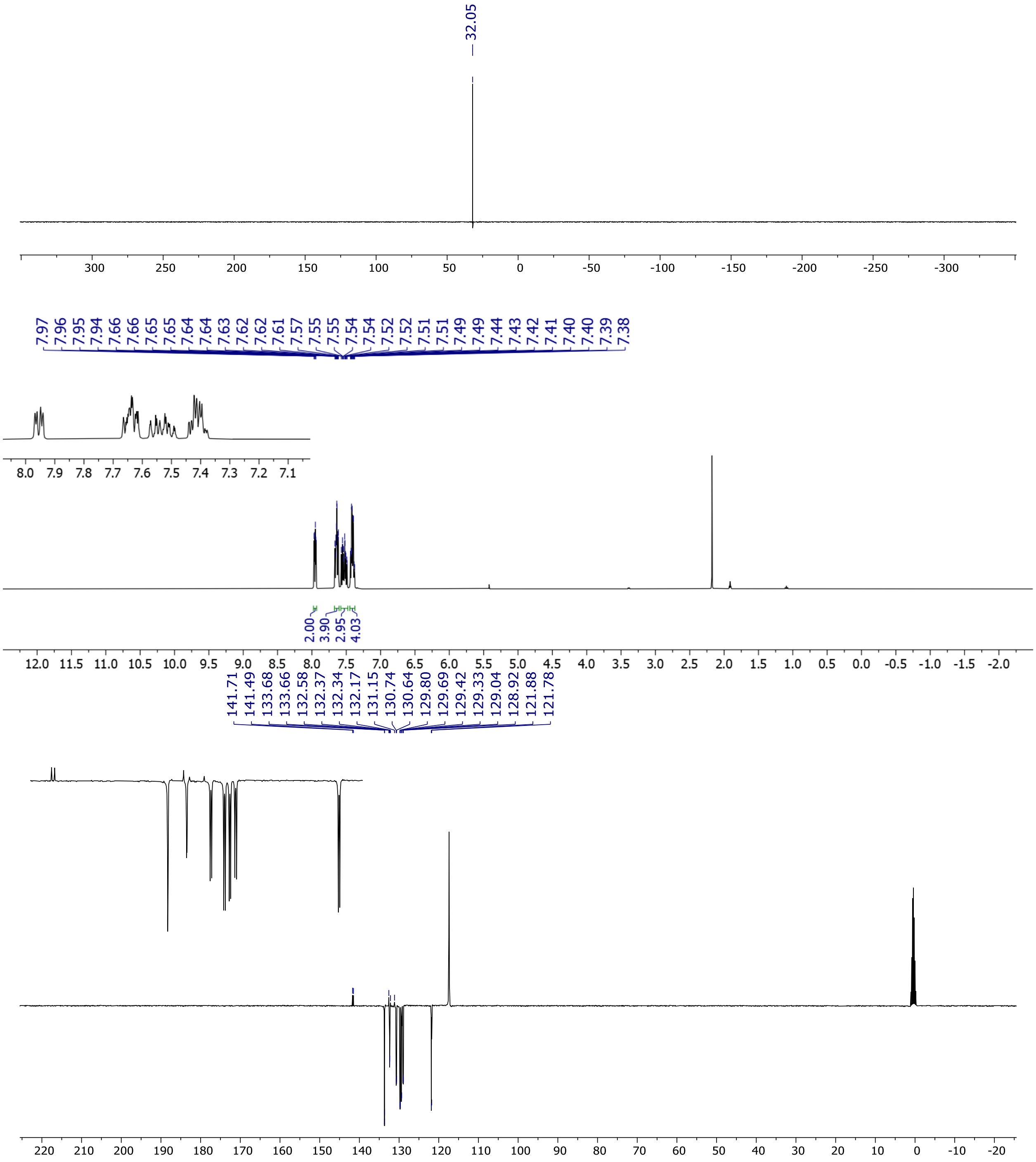

Fig. S43. *^{31}P (162 MHz), ^{1}H (400 MHz) and ^{13}C (101 MHz) NMR of **1j** in ACN-d_3*

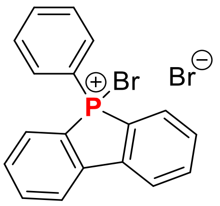

3j-Br

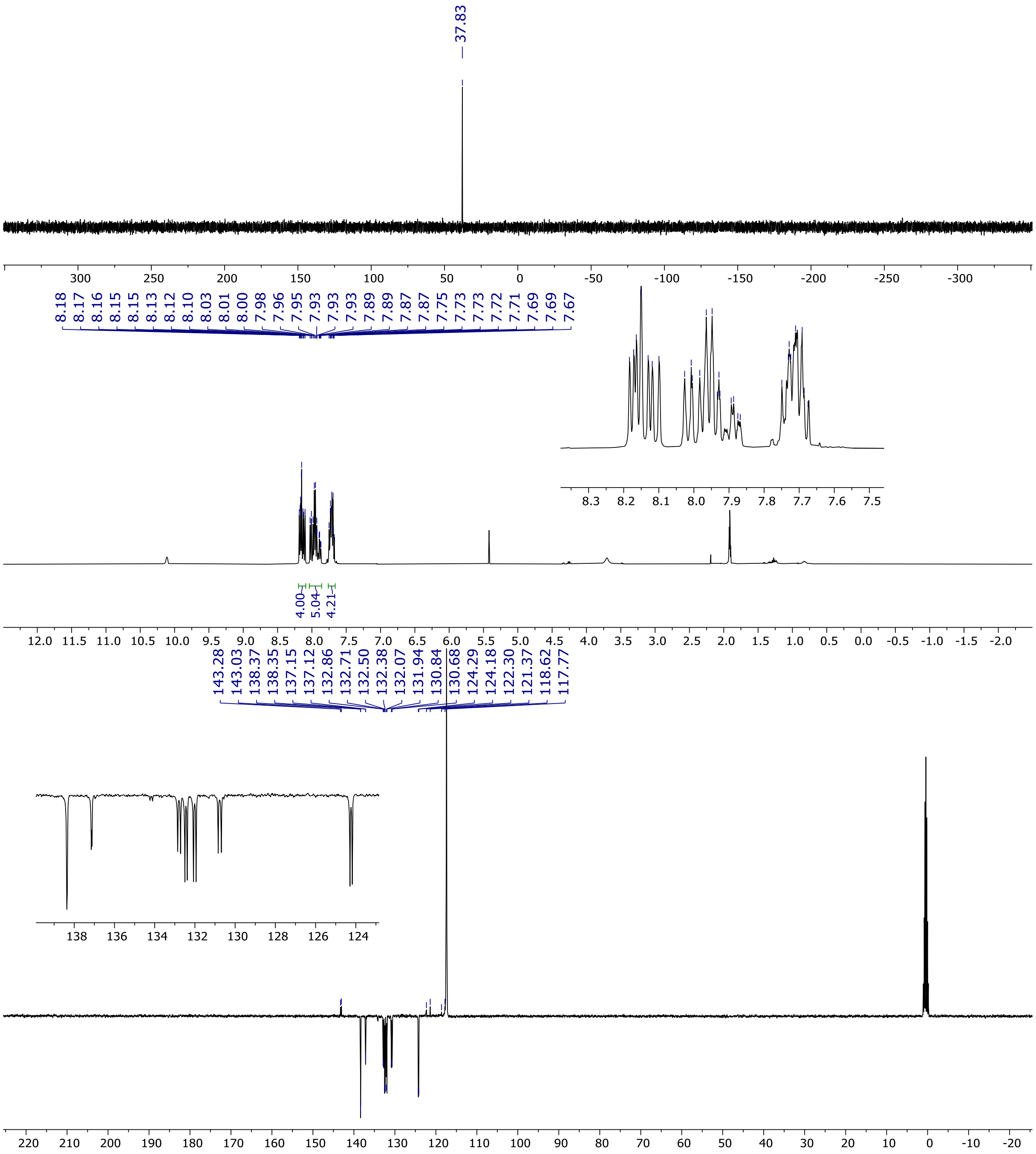

Fig. S44. ^{31}P (162 MHz), ^{1}H (400 MHz) and ^{13}C (101 MHz) NMR of ***3j-Br*** in ACN-d_3

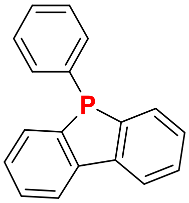

3j

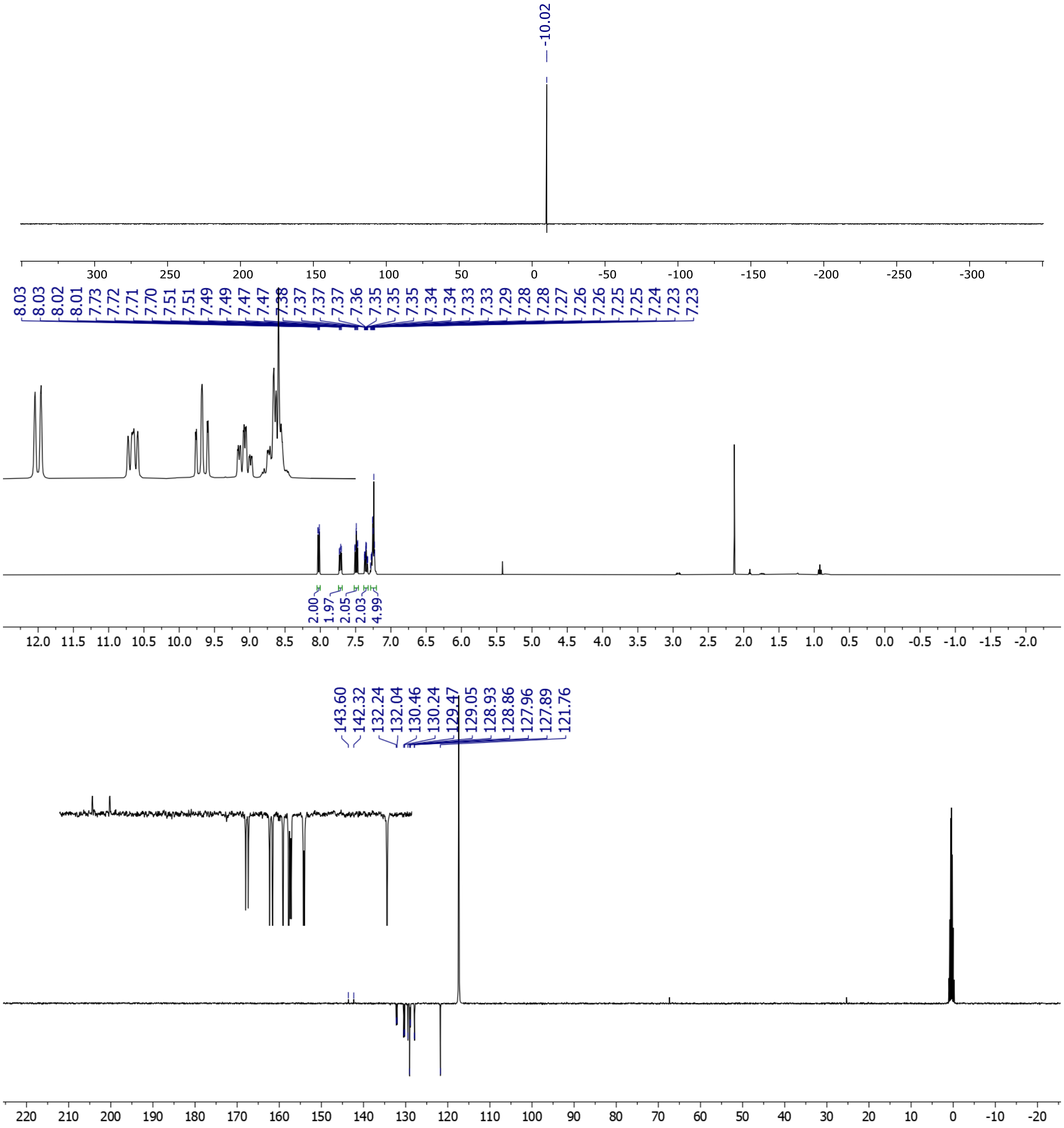

Fig. S45. *^{31}P (162 MHz), ^{1}H (400 MHz) and ^{13}C (101 MHz) NMR of **3j** in ACN-d_3*

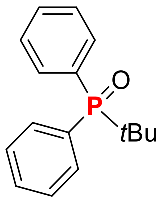

1k

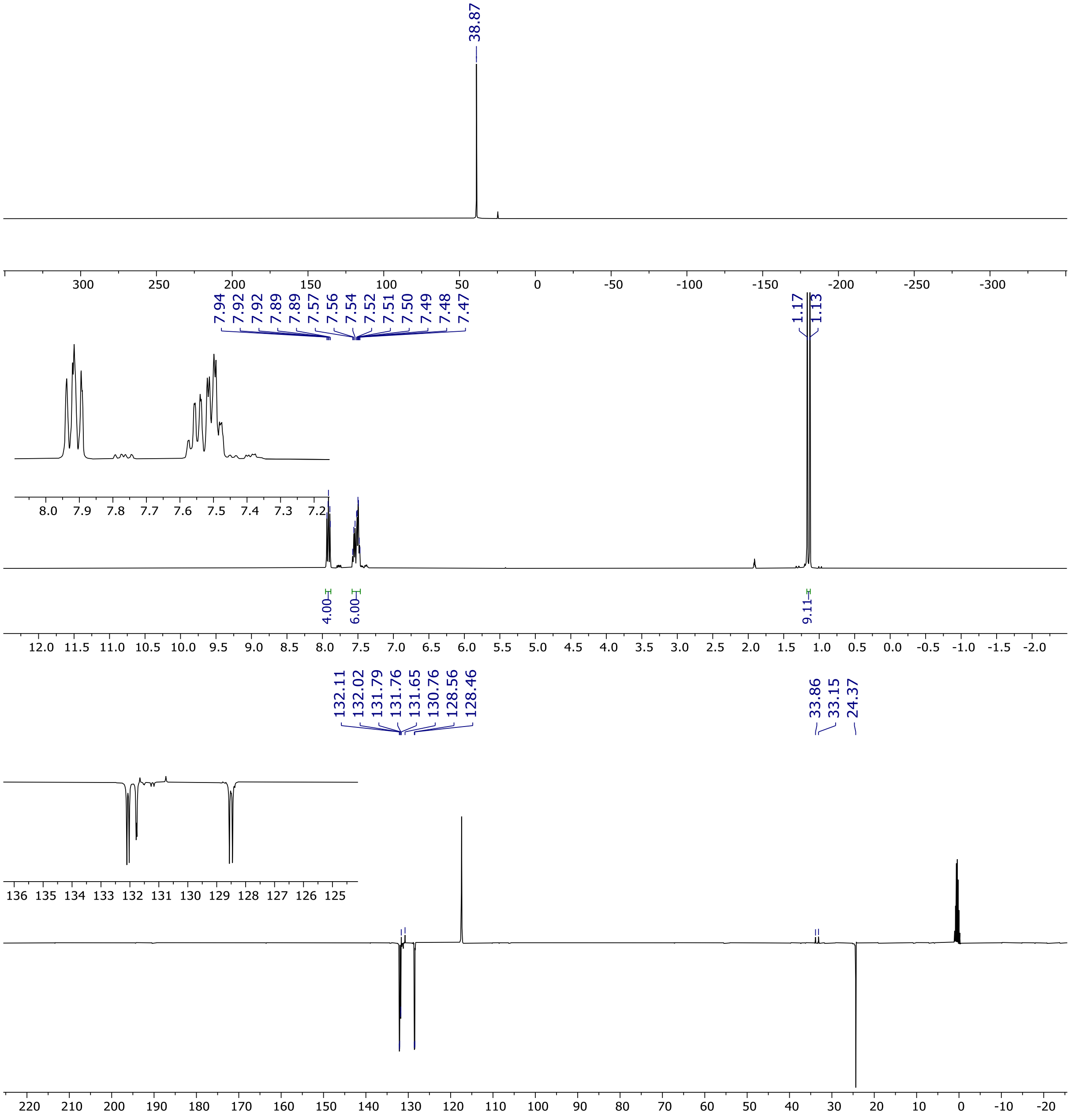

Fig. S46. *^{31}P (162 MHz), ^{1}H (400 MHz) and ^{13}C (101 MHz) NMR of **1k** in ACN-d_3*

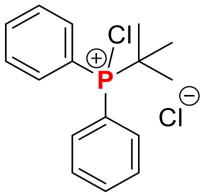

2l-Cl

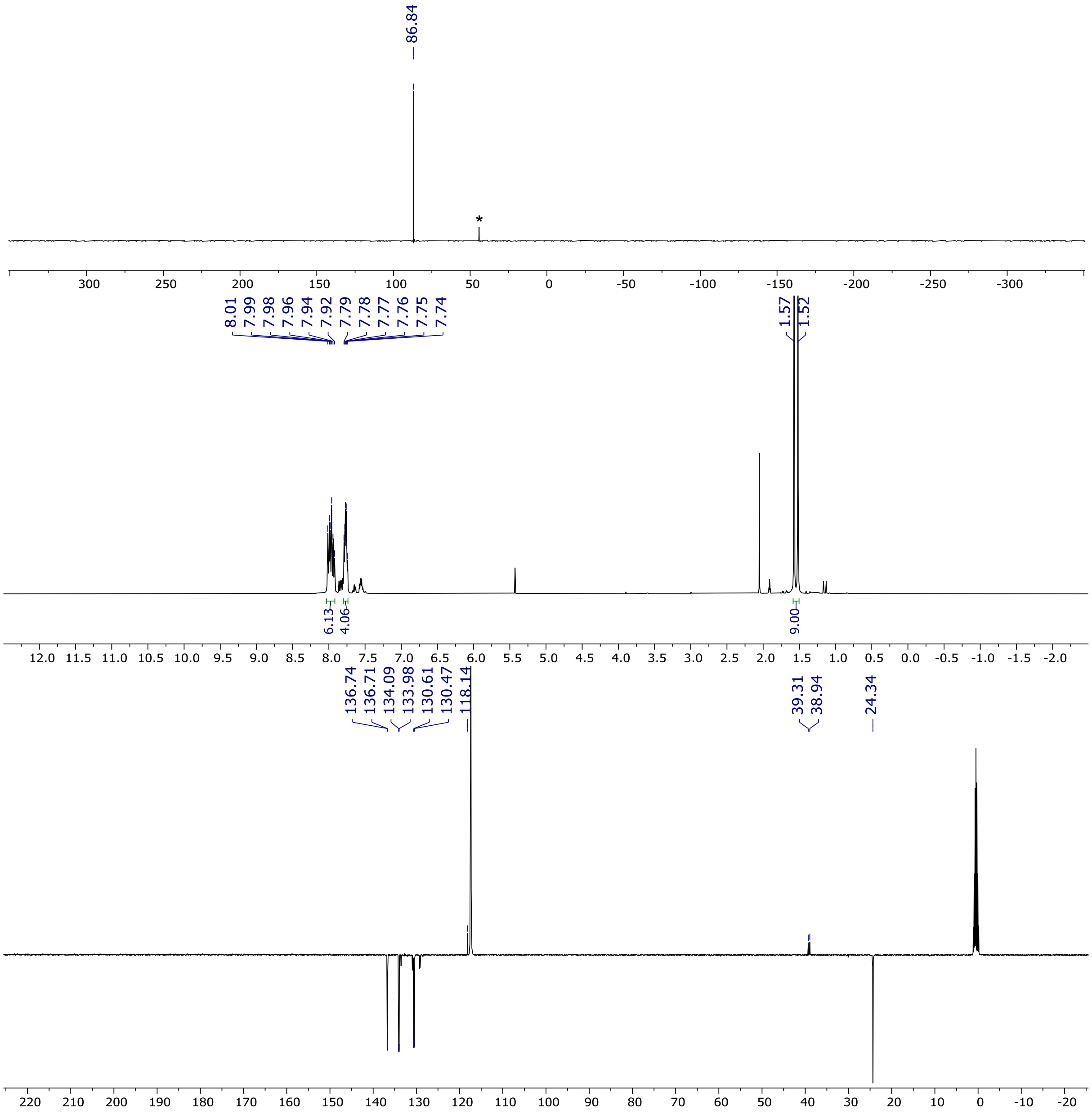

Fig. S47. *^{31}P (162 MHz), ^{1}H (400 MHz) and ^{13}C (101 MHz) NMR of* ***2k-Cl*** *in ACN-d_3. * marks the signal of unknown product*

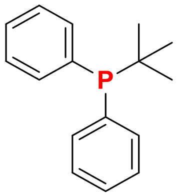

3k

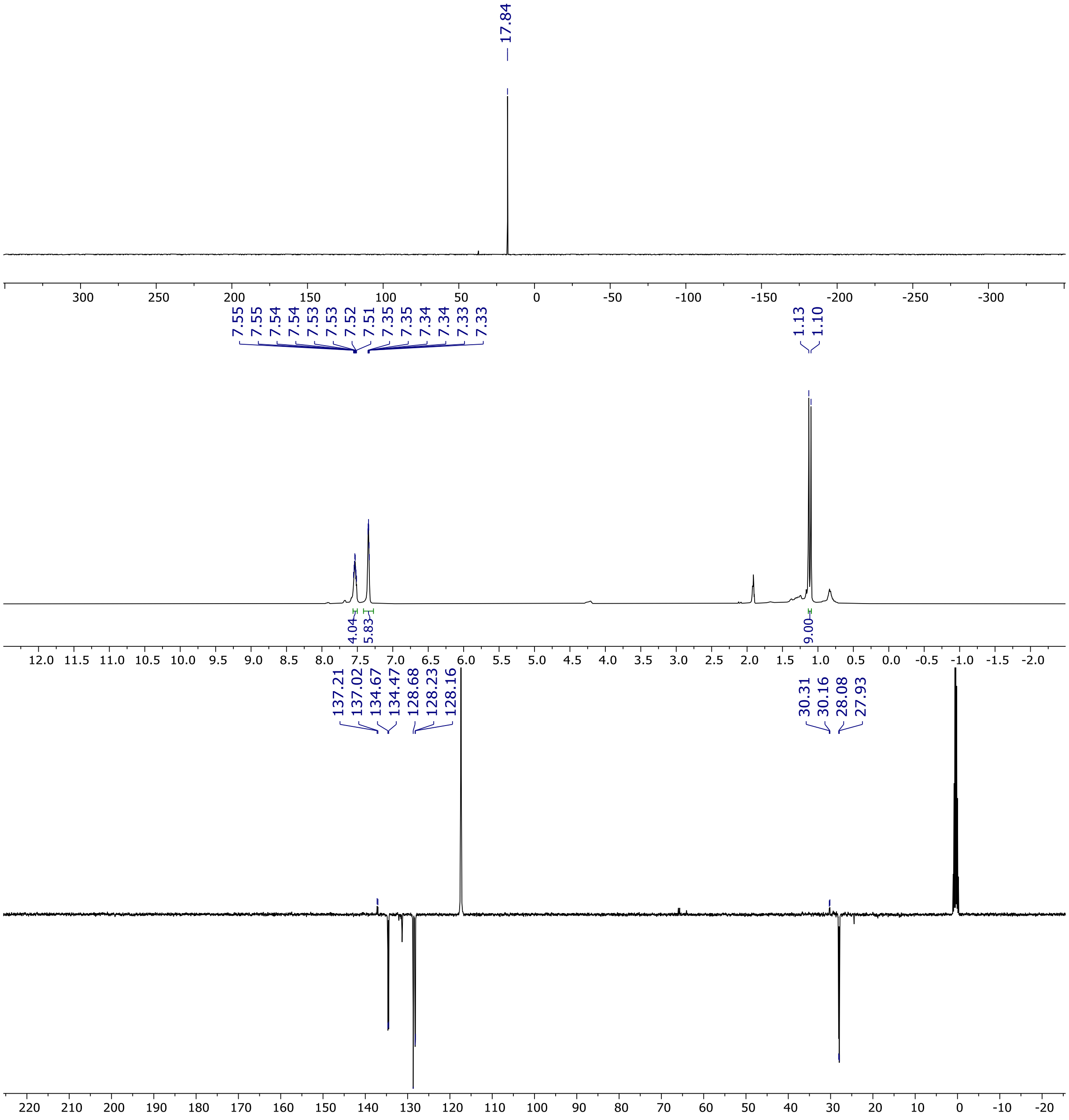

Fig. S48. *^{31}P (162 MHz), ^{1}H (400 MHz) and ^{13}C (101 MHz) NMR of **3k** in ACN-d_3*

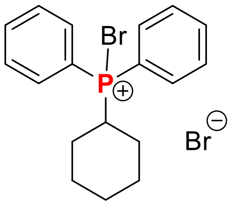

2l-Br

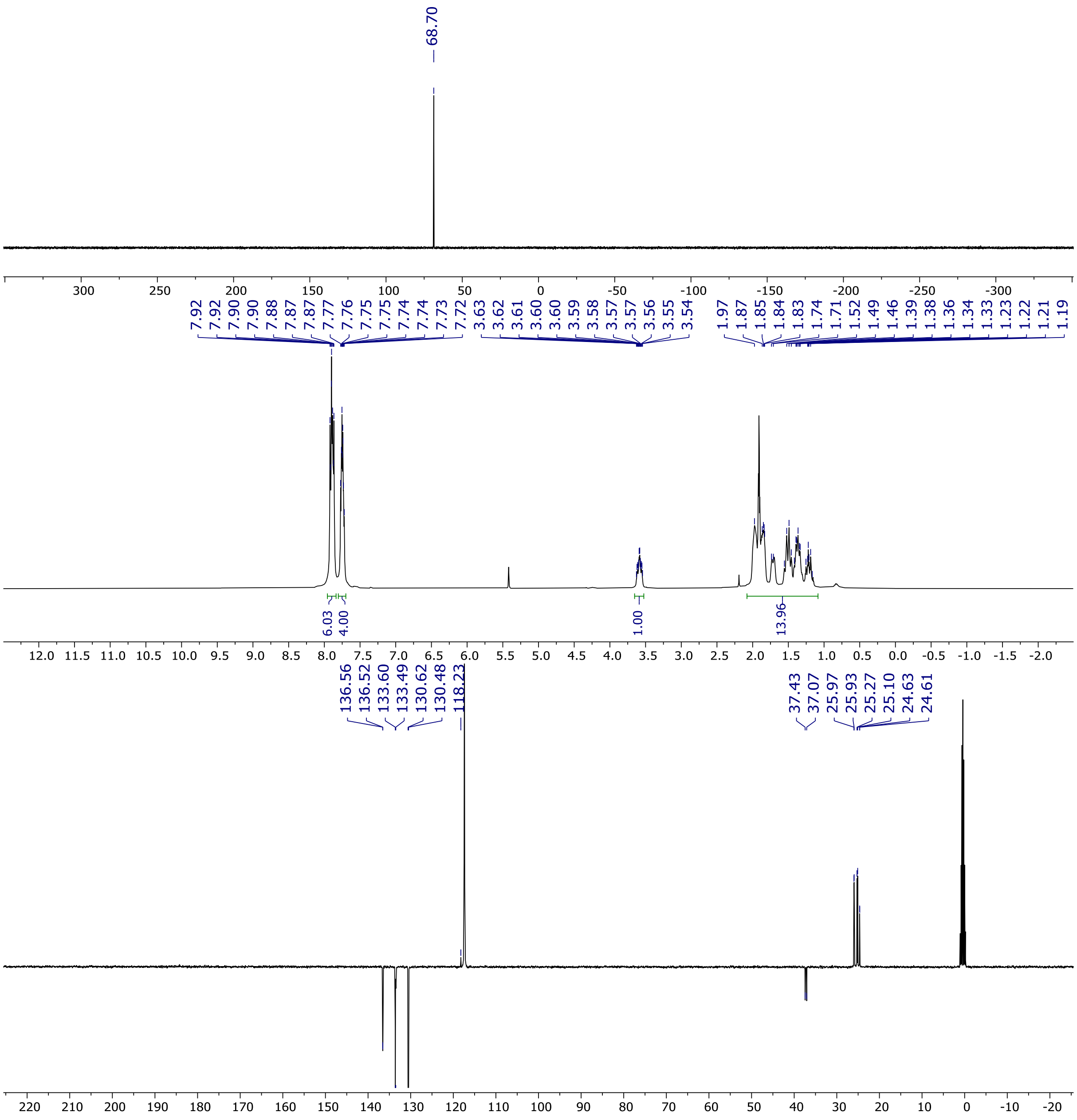

Fig. S49. *^{31}P (162 MHz), ^{1}H (400 MHz) and ^{13}C (101 MHz) NMR of* ***2l-Br*** *in ACN-d_3*

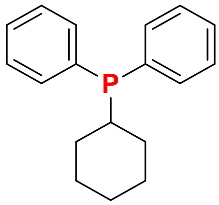

3l

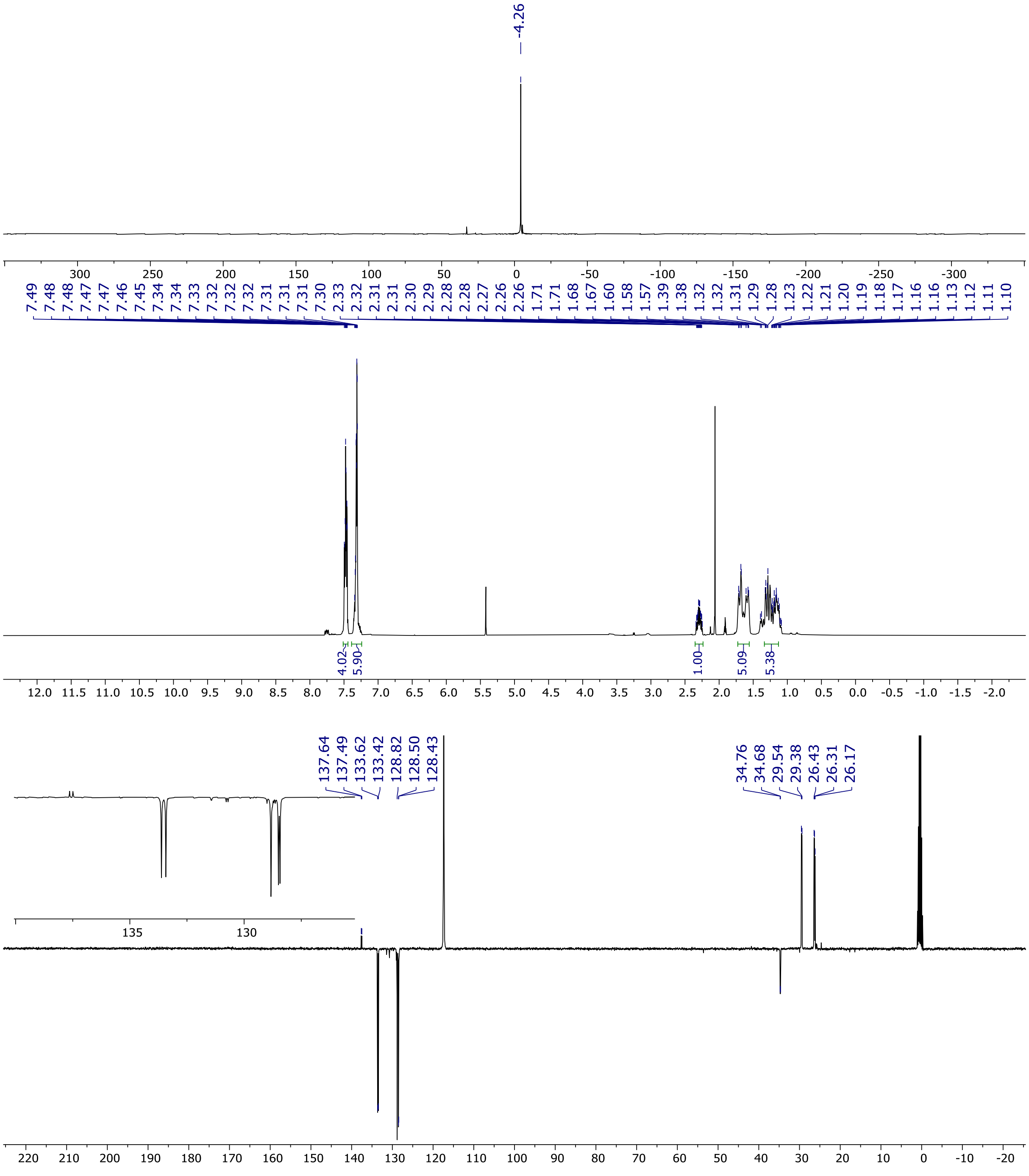

Fig. S50. *^{31}P (162 MHz), ^{1}H (400 MHz) and ^{13}C (101 MHz) NMR of **3l** in ACN-d_3*

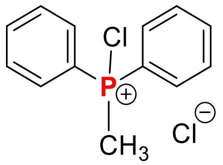

2m-Cl

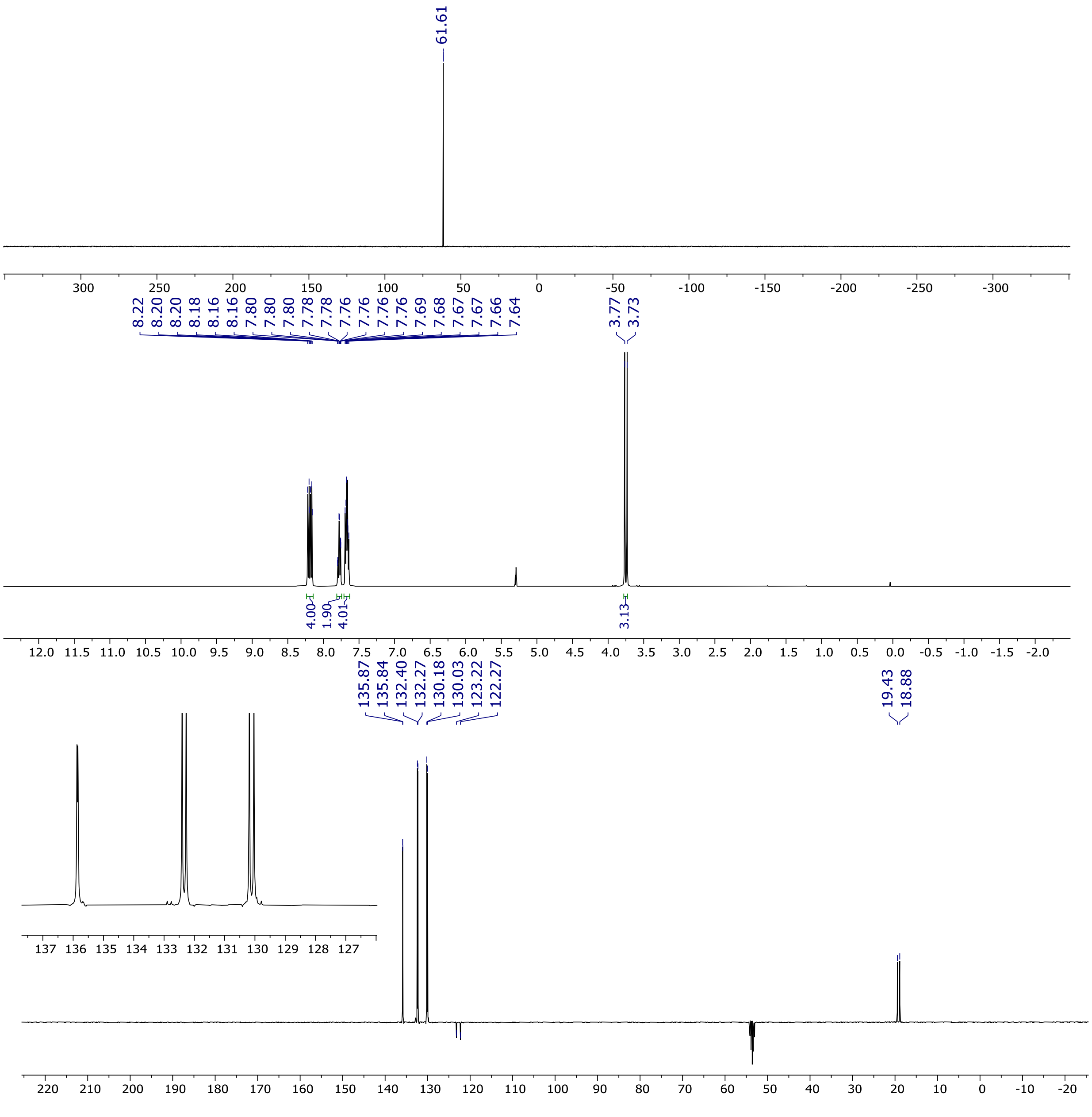

Fig. S51. ^{31}P (162 MHz), ^{1}H (400 MHz) and ^{13}C (101 MHz) NMR of **2m-Cl** in DCM-d_2

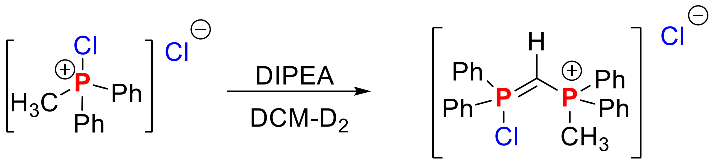

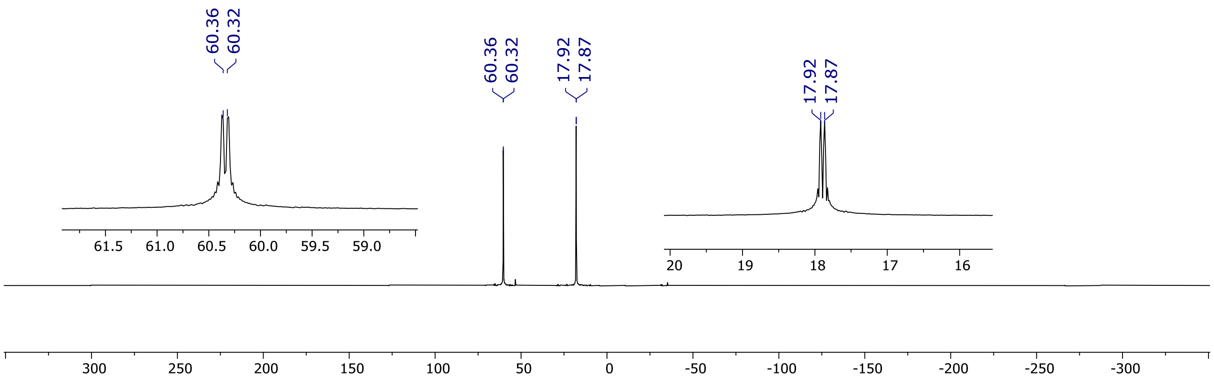

Fig. S52. *Crude* ^{31}*P (162 MHz) NMR of* ***2m-Cl*** *and DIPEA in DCM-d*$_2$

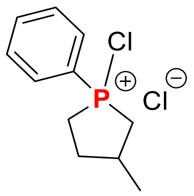

2n-Cl

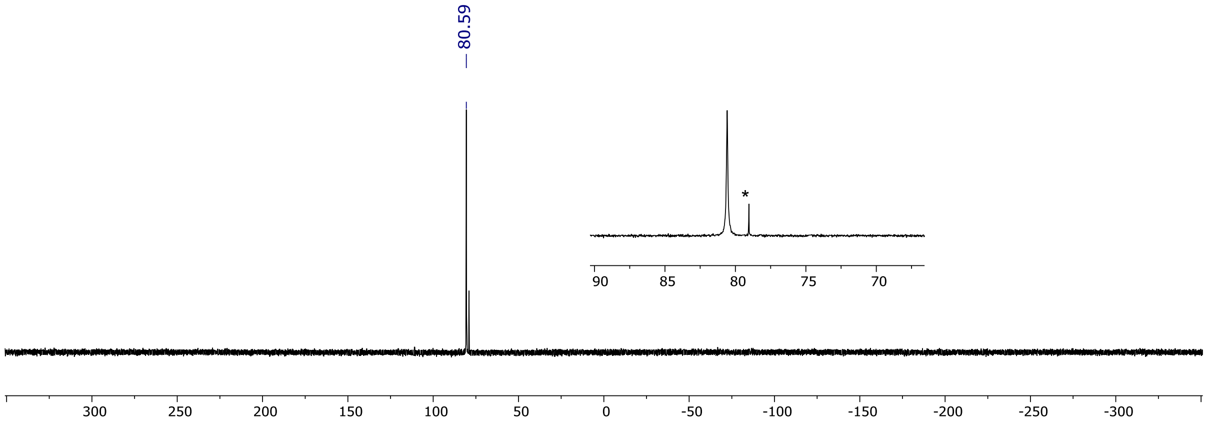

Fig. S53. *^{31}P (162 MHz) NMR of* ***2n-Cl*** *in ACN-d_3. * marks the signal of unknown compound*

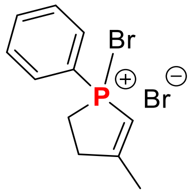

2o-Br

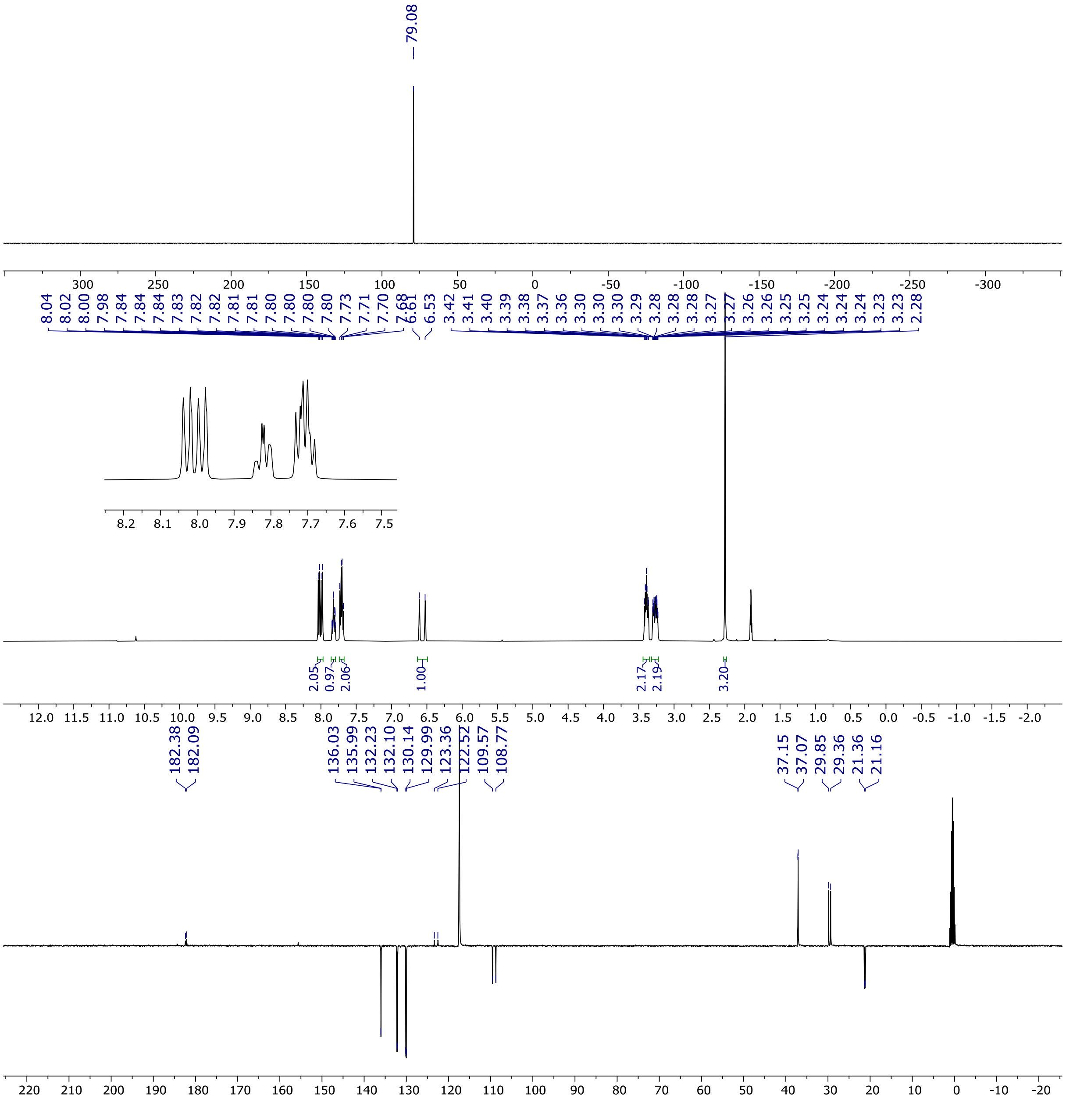

Fig. S54. *^{31}P (162 MHz), ^{1}H (400 MHz) and ^{13}C (101 MHz)NMR of* ***2o-Br*** *in ACN-d_3*

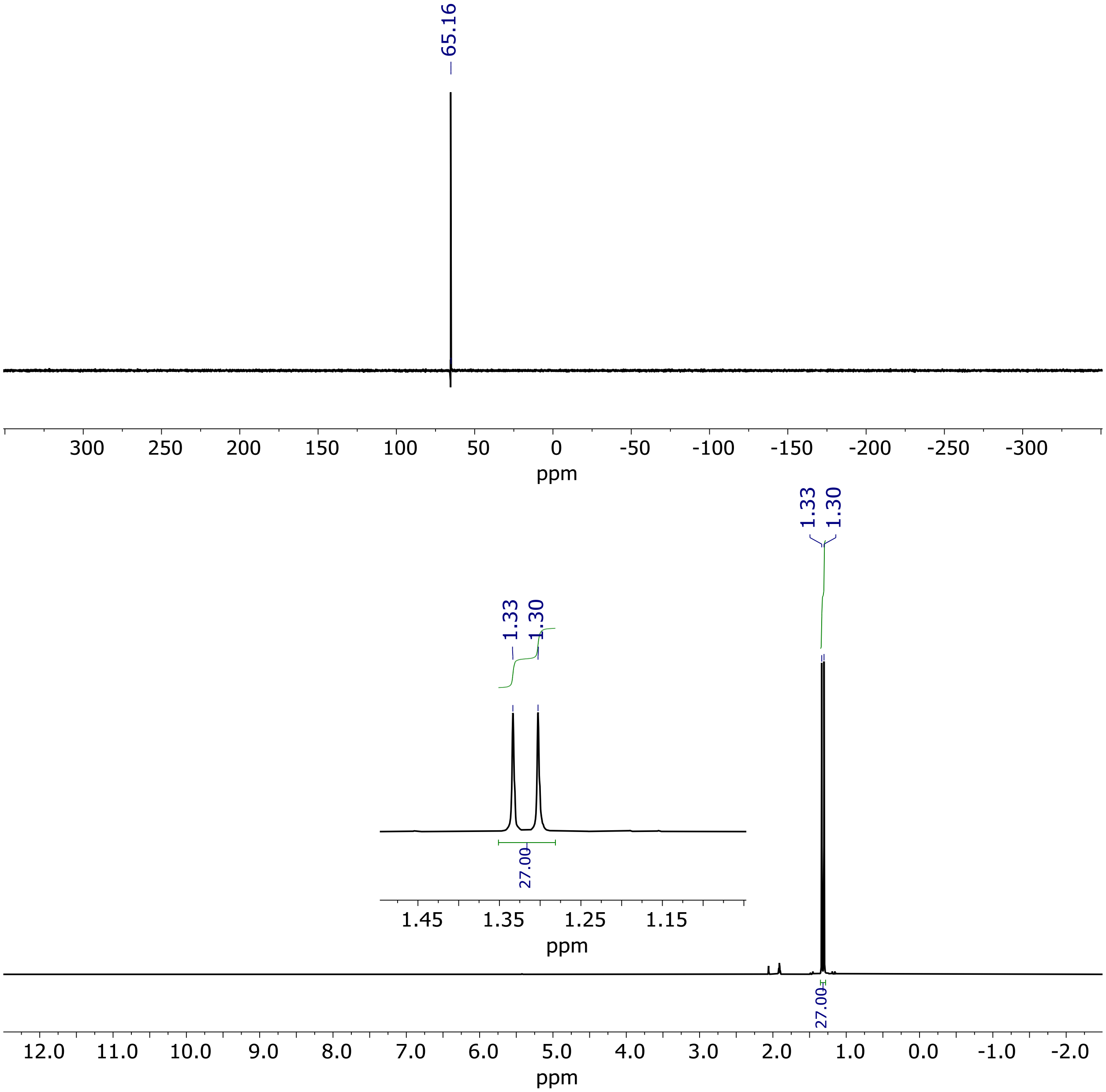

Fig. S55. *^{31}P (162 MHz) and ^{1}H (400 MHz) NMR of **1p** in ACN-d_3*

7. UV-Vis studies of EDA formation

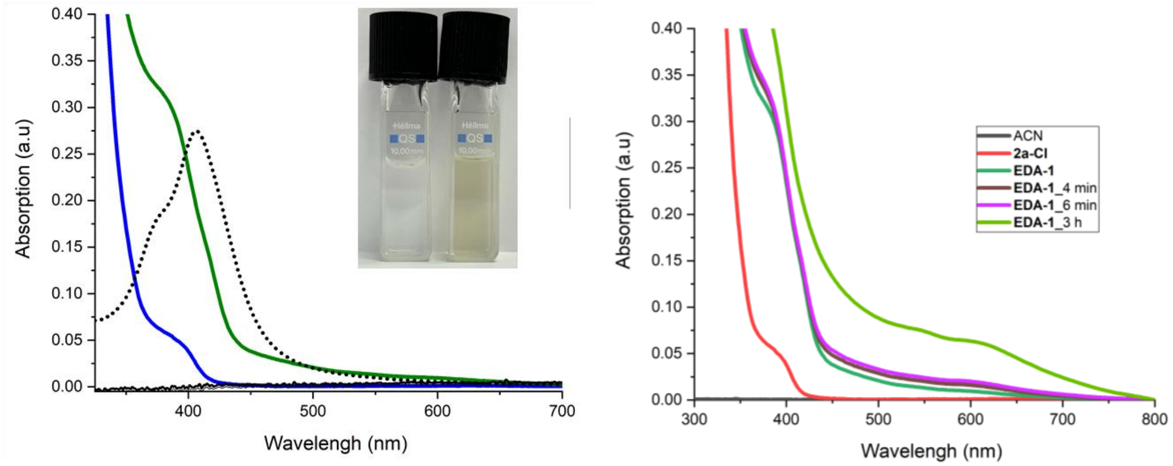

Fig. S56. *UV-Vis spectrum of 2a-Cl, EDA-1 (right) and its evolution over time in ACN (left).*

8. Cyclic voltammetry

Cyclic voltammograms (CVs) were recorded for **2a-Cl** in the absence and presence of DIPEA and *n*Pr_3N. The measurements were carried out using a standard 3 electrode cell equipped with a glassy carbon working electrode (3mm diameter), a platinum counter electrode and a Ag/Ag^+ reference electrode in ACN in the presence of 0.1M Bu_4NPF_6 as supporting electrolyte. All electrochemical measurements were performed in the glove box using ACN prepared as described in the general information section on p. 3 of this ESI using an Autolab PGSTAT 204N potentiostat by Metrohm. All data are referenced vs $Fc^{+/0}$ that was added to the electrolyte solution after each experiment. Scan rate was 100 mV/s. All measurements were carried out in the glove box at room temperature. The CVs were recorded in the cathodic direction first, starting at -0.5V; the scans were reversed at -3.2V. Between the cycles, the glassy carbon electrode was polished using 5µm Al_2O_3 suspension on polishing cloths, sonicated after polishing, rinsed with water and acetone, dried and reintroduced into the glove box.

The structural integrity of **2a-Cl** in the presence of the supporting electrolyte was ensured by parallel NMR scale reactions using the same batch of **2a-Cl** and supporting electrolyte. Illumination of these samples resulted in no deviation from the original protocol and obtained yields.

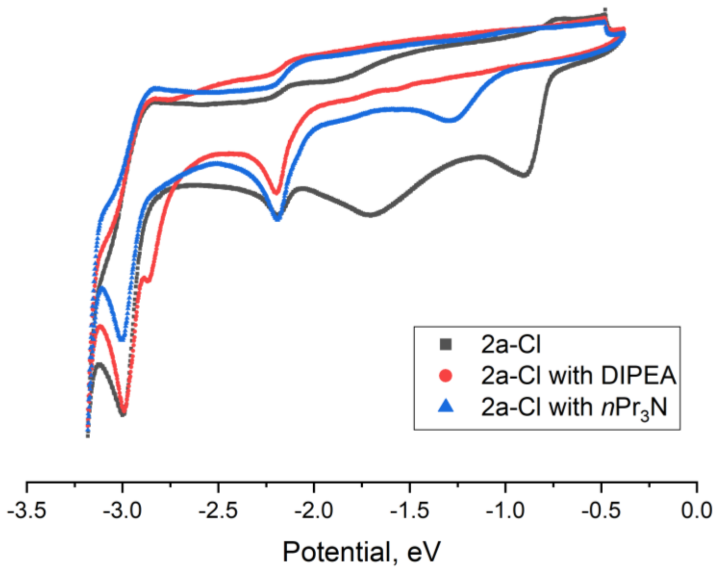

Fig. S57. *CV traces of* ***2a-Cl*** *(1mM in 0.1M* Bu_4NPF_6 *in* CH_3CN*) as well as its EDA complexes with DIPEA (5 mM) and* nPr_3N *(5 mM). IUPAC convention was used for the plot, i.e. positive potential to the right and positive anodic (oxidation) current upwards. The CVs were recorded in the cathodic direction first, starting at -0.5V; the scans were reversed at -3.2V. All potentials are relative to the* $Fc^{+/0}$ *couple which was added as internal standard after each experiment.*

As expected, none of the CVs are electrochemically reversible, prohibiting the determination of thermodynamic data that could be used for the calculation of driving forces for electron transfer. In fact, even peak assignments in the CVs are not trivial. Despite the lack of quantitative data, certain trend can be observed. The CVs of **2a-Cl**, as well as its EDA complexes with DIPEA and *n*Pr_3N show a cathodic peak around -2.2V. As this feature is observed in all three CVs, it is assigned to **2a-Cl**. The EDA complexes are expected to be reduced at more cathodic potential due to the increased electron density caused by the electron rich, coordinating amines. The

observation of **2a-Cl** in the CVs of the EDA complexes can be expected due to the following equilibrium, which, upon electrochemical reduction, is pulled towards **2a-Cl** on the CV timescale, following Le Chatelier's principle.

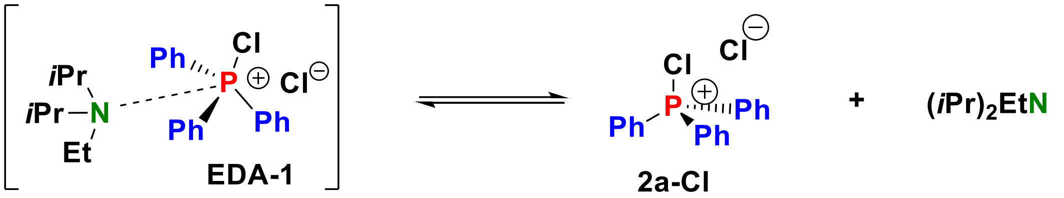

The features more anodic to the **2a-Cl** wave are assigned to species that are formed between amine donors and residual oxalyl chloride/bromide.

9. EPR spectroscopy

Various efforts have been made to detect *P*-based radical intermediates by electron paramagnetic resonance (EPR) spectroscopy. EPR measurements were conducted at room temperature on a Bruker EMX Micro CW-EPR spectrometer (Bruker, USA) equipped with an ER 4119HS resonator. The microwave frequency was 9.844 GHz and the modulation frequency 100 kHz. Microwave power was set to 2mW. Samples for measurements were prepared according to standard protocol described in section 3 of this document. As shown below, a radical species that is barely beyond the noise level is observed, but we do not feel confident to assign this signal to any specific species. The experiment demonstrates that the lifetime of any radical intermediate species is short, precluding the accumulation of appreciable concentrations that would allow for a meaningful characterization. The experiments indirectly support the proposed mechanism in that no radical species are proposed at later stages of the sequence, and radicals are only proposed directly following the light-driven charge separation. It is likely that the subsequent chemistry occurs directly after radical formation, potentially even in the same solvent cage.

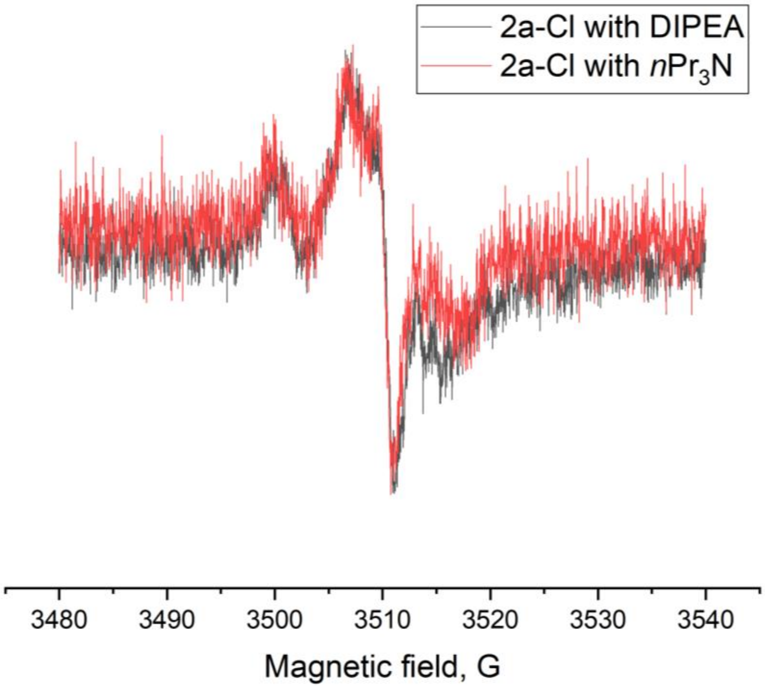

Fig. S58*. EPR traces of reaction mixtures under illumination.*

10. References

[1] R. M. Denton, J. An, B. Adeniran, A. J. Blake, W. Lewis, A. M. Poulton, *The Journal of Organic Chemistry* **2011**, *76*, 6749-6767.

[2] A. J. Stepen, M. Bursch, S. Grimme, D. W. Stephan, J. Paradies, *Angewandte Chemie International Edition* **2018**, *57*, 15253-15256.

[3] A. Ding, S. Li, Y. Chen, R. Jin, C. Ye, J. Hu, H. Guo, *Tetrahedron Letters* **2018**, *59*, 3880-3883.

[4] J. Xue, Y.-S. Zhang, Z. Huan, J.-D. Yang, J.-P. Cheng, *Journal of the American Chemical Society* **2023**, *145*, 15589-15599.

[5] J. Yang, J. Xiao, T. Chen, L.-B. Han, *The Journal of Organic Chemistry* **2016**, *81*, 3911-3916.

[6] A. Bowden, S. J. Coles, M. B. Pitak, A. W. G. Platt, *Inorganic Chemistry* **2012**, *51*, 4379-4389.

[7] Ł. Kapuśniak, P. N. Plessow, D. Trzybiński, K. Woźniak, P. Hofmann, P. I. Jolly, *Organometallics* **2021**, *40*, 693-701.

[8] Q. Yin, Y. Ye, G. Tang, Y.-F. Zhao, *Spectrochimica Acta Part A: Molecular and Biomolecular Spectroscopy* **2006**, *63*, 192-195.

[9] K. Nikitin, H. Müller-Bunz, D. Gilheany, *Chemical Communications* **2013**, *49*, 1434.

[10] M. Kuroboshi, T. Yano, S. Kamenoue, H. Kawakubo, H. Tanaka, *Tetrahedron* **2011**, *67*, 5825-5831.

[11] P. Li, R. Wischert, P. Métivier, *Angewandte Chemie International Edition* **2017**, *56*, 15989-15992.

[12] H. Vorbrüggen, K. Krolikiewicz, *Helvetica Chimica Acta* **1993**, *76*, 819-825.

[13] K. Nikitin, E. V. Jennings, S. Al Sulaimi, Y. Ortin, D. G. Gilheany, *Angewandte Chemie International Edition* **2018**, *57*, 1480-1484.

[14] J. Dupré, A.-C. Gaumont, S. Lakhdar, *Organic Letters* **2017**, *19*, 694-697.

[15] Z. Xu, P. Wang, Q. Chen, M. Cai, *Journal of Organometallic Chemistry* **2018**, *866*, 50-58.

[16] J. Wang, X. Xu, Y. Tian, C. Yao, R. Liu, L. Li, *Journal of Materials Chemistry C* **2015**, *3*, 2856-2864.

[17] X. Wei, Z. Lu, X. Zhao, Z. Duan, F. Mathey, *Angewandte Chemie International Edition* **2015**, *54*, 1583-1586.

[18] A. Kermagoret, P. Braunstein, *Dalton Trans.* **2008**, 822-831.

[19] L. Miao, A. Yeganeh-Salman, J. Yeung, D. W. Stephan, *Dalton Transactions* **2022**, *51*, 14049-14053.
